# Machine learning reveals temperature as a key predictor of dengue risk across Thailand’s provinces: A 20-year analysis

**DOI:** 10.1371/journal.pntd.0014590

**Published:** 2026-07-28

**Authors:** Pikkanet Suttirat, Sudarat Chadsuthi, Supassorn Aekthong, Joacim Rocklöv, Dominique J. Bicout, Peter Haddawy, Myat Su Yin, Saranath Lawpoolsri, Charin Modchang

**Affiliations:** 1 Medical Biophysics & Disease Modeling Group, Department of Physics, Faculty of Science, Mahidol University, Bangkok, Thailand; 2 Center for Disease Modeling, Faculty of Science, Mahidol University, Bangkok, Thailand; 3 Department of Physics, Faculty of Science, Naresuan University, Phitsanulok, Thailand; 4 Department of Public Health and Clinical Medicine, Section of Sustainable Health, Umeå University, Umeå, Sweden; 5 Heidelberg Institute of Global Health, Heidelberg University, Heidelberg, Germany; 6 Interdisciplinary Center of Scientiﬁc Computing, Heidelberg University, Heidelberg, Germany; 7 Univ. Grenoble Alpes, CNRS, UMR 5525, VetAgro Sup, Grenoble INP, TIMC, Grenoble, France; 8 Faculty of ICT, Mahidol University, Nakhon Pathom, Thailand; 9 Bremen Spatial Cognition Center, University of Bremen, Bremen, Germany; 10 Department of Tropical Hygiene, Faculty of Tropical Medicine, Mahidol University, Bangkok, Thailand; 11 Centre of Excellence in Mathematics, MHESI, Bangkok, Thailand; Colorado State University, UNITED STATES OF AMERICA

## Abstract

**Background:**

Dengue fever remains a critical public health challenge in Thailand, with transmission dynamics driven by complex interactions between environmental and socioeconomic factors. Understanding these predictive factors is essential for developing robust forecasting systems.

**Methods:**

We developed a machine learning framework to classify spatiotemporal dengue risk and identify key predictive factors across Thailand. We analyzed 20 years of monthly dengue hemorrhagic fever surveillance data (2003–2022) from 77 provinces, integrating 54 environmental, climatic, and socioeconomic features. We benchmarked four candidate classifiers — logistic regression, support vector machines, random forests, and eXtreme Gradient Boosting (XGBoost) — and selected XGBoost on the basis of performance across six metrics. SHapley Additive exPlanations (SHAP) were used to interpret feature contributions. The dataset was stratified into training (2003–2016) and testing periods, with the latter subdivided into pre-COVID-19 (2017–2019), COVID-19 (2020–2021), and post-COVID-19 (2022) phases.

**Results:**

The XGBoost model achieved an AUC of 0.80 in pre-pandemic testing and 0.74 across the combined during- and post-pandemic period. Temperature dominated the feature-importance ranking, comprising seven of the top ten features, with non-linear thresholds near 21°C for 1-month lagged minimum temperature and near 32°C for 3-month lagged maximum temperature — values that align with established biological constraints on *Aedes aegypti*–mediated transmission. Precipitation features contributed minimally to model predictions, while a higher Gross Provincial Product was associated with increased dengue risk, consistent with predominantly urban transmission patterns. Model performance deteriorated significantly during the COVID-19 pandemic (AUC = 0.62 in 2021), with systematic overprediction indicating that non-environmental factors operating outside the model dominated dengue dynamics during this period.

**Conclusions:**

Temperature is the dominant predictor of dengue risk in Thailand, and the thresholds we recover correspond closely to known biological constraints on vector competence. Environmentally driven prediction is reliable under stationary conditions but degrades substantially during periods of major societal disruption, underscoring the need to integrate behavioral and surveillance-coverage indicators alongside environmental predictors when applying such models in real time.

## Author summary

This study used machine learning to predict the risk of dengue fever outbreaks across Thailand’s provinces from 2003 to 2022. By analyzing climate data, economic indicators, and satellite imagery, our machine learning model could accurately identify high-risk areas about 80% of the time. We found that temperature is the most important factor determining where and when dengue will spread. Additionally, we identified a critical temperature threshold where dengue transmission essentially stops when minimum temperatures drop below approximately 21°C. Interestingly, rainfall patterns, which are often emphasized in dengue predictions, played a much smaller role than expected. Higher economic development was also found to be associated with an increased risk of dengue, probably due to urbanization creating ideal conditions for mosquitoes to breed all year round and higher human population density that facilitates virus transmission. However, the COVID-19 pandemic disrupted the model’s accuracy, causing it to predict higher dengue risk than actually occurred from 2020 to 2022, likely due to the COVID-19 control measures that climate data alone could not capture. This research demonstrates both the power and limitations of using environmental and socioeconomic data to predict dengue risks in Thailand.

## Introduction

Dengue fever represents one of the most rapidly expanding vector-borne diseases globally, with an estimated 390 million infections occurring annually across more than 100 endemic countries in tropical and subtropical regions [1–3]. Over the past five decades, the global disease burden has increased dramatically, with a 30-fold rise in incidence [4]. This escalation is particularly pronounced in Southeast Asia, which bears approximately 70% of the global dengue burden [1]. Among Southeast Asian nations, Thailand consistently reports some of the highest incidence rates in the region, making it a critical focal point for understanding dengue transmission dynamics [5].

The dengue virus comprises four distinct serotypes and is transmitted to humans primarily through the bites of infected *Aedes* mosquitoes in tropical and subtropical environments [2,3,6]. Clinical manifestations range considerably in severity, from asymptomatic infections or mild undifferentiated fever to classical dengue fever (DF), and in severe cases, to life-threatening conditions such as dengue hemorrhagic fever (DHF) or dengue shock syndrome (DSS) [2,3]. The most severe forms can lead to plasma leakage, hemorrhage, and organ impairment, resulting in substantial morbidity and mortality [7].Thailand’s experience with dengue dates back to 1958, when the country recorded its first epidemic of dengue hemorrhagic fever (DHF) [8,9]. Since then, the disease has evolved into a persistent public health challenge, exhibiting complex spatiotemporal patterns driven by the interplay of climatic conditions, socioeconomic factors, and vector ecology [10,11]. Understanding these patterns is crucial for developing effective prediction and control strategies.

The epidemiology of dengue is shaped by a complex interplay of socioeconomic, demographic, and infrastructural factors [12–14]. While several studies have documented associations between low socioeconomic status and increased local dengue risk [13,15], the relationship is more nuanced than initially understood. Rapid urbanization, high population density, and inadequate waste management systems create favorable conditions for disease emergence and spread [16,17]. However, recent evidence challenges the traditional view of dengue as primarily an urban disease of poverty. Indeed, the virus now affects both rural and urban populations across diverse socioeconomic strata, indicating that transmission patterns have evolved beyond simple associations with urbanization or economic disadvantage [18,19].

Environmental factors play a pivotal role in dengue transmission dynamics. Climate-related variables, particularly temperature and rainfall patterns, along with the degree of urban development, fundamentally shape local dengue risk profiles [1,20,21]. Temperature exerts particularly strong effects on transmission by influencing critical mosquito biological parameters, including biting frequency, fecundity, developmental rates, adult survival, and the efficiency of egg-to-adult development [22,23]. These temperature-dependent processes create distinct geographic and seasonal patterns of transmission risk.

Traditional approaches to dengue forecasting have relied predominantly on epidemiological surveillance data combined with basic statistical models that incorporate rainfall and temperature variables [24–26]. However, these conventional methods often fail to capture the complex, non-linear relationships that characterize the interactions between multiple environmental and socioeconomic transmission drivers [27]. This limitation has prompted researchers to explore more sophisticated analytical approaches.

Recent advances in machine learning offer promising alternatives for disease prediction, with ensemble methods such as gradient boosting demonstrating remarkable performance in capturing intricate feature interactions that traditional models miss [27–29]. These methods can process high-dimensional data and identify complex patterns that would be difficult to detect using conventional statistical approaches.

Despite growing interest in applying machine learning to dengue prediction, significant knowledge gaps still persist [28,30]. Most existing models suffer from a limited geographic scope or analyze only short time periods [28,30,31]. Many rely disproportionately on precipitation as a primary predictor, despite mounting evidence of inconsistent associations between rainfall and dengue incidence [32]. Additionally, few models provide interpretable insights into which features drive their predictions, limiting their utility for public health decision-making [27].

Additionally, the impact of major societal disruptions on the performance of environmentally driven prediction models remains poorly understood, with few studies systematically evaluating how events like the COVID-19 pandemic affect the performance of these models [33]. Pandemic interventions, including mobility restrictions and lockdown measures, likely altered dengue transmission patterns through mechanisms independent of environmental conditions [33–36]. The COVID-19 pandemic offers a unique opportunity to assess the model’s resilience and determine the relative contributions of environmental versus behavioral factors in disease transmission. Understanding how prediction systems perform during such extraordinary circumstances is crucial for developing robust forecasting tools that can maintain accuracy in the face of future societal disruptions.

To address these critical gaps, this study develops and evaluates a machine learning framework for spatiotemporal dengue risk classification across Thailand. We employ eXtreme Gradient Boosting (XGBoost) combined with SHapley Additive exPlanations (SHAP) to analyze two decades of dengue surveillance data (2003–2022) integrated with comprehensive environmental and socioeconomic variables. We focused on DHF cases as a representative measure of dengue risk because they balance clinical severity with reliable reporting. Milder DF cases are systematically under-reported [37–39], whereas dengue shock syndrome (DSS), although the most severe manifestation, is too infrequent to support province-level monthly classification. Across the 18,240 province-months of the 2003–2022 study period, 59.0% recorded no DSS cases, and the median DSS incidence is zero; applying the same median-incidence rule used to define our binary target therefore labels every province-month as high-risk for DSS, in contrast to DHF, whose median incidence (2.78 per 100,000) yields the balanced high- and low-risk split used here (see Risk classification section for more details). Our specific objectives are threefold: first, to develop a robust classification model capable of quantifying the relative importance and interaction effects of diverse environmental and socioeconomic drivers; second, to identify critical thresholds in key predictors that govern transitions in dengue transmission risk; and third, to evaluate model performance across different epidemiological periods, with particular emphasis on assessing how the COVID-19 pandemic affected prediction accuracy. Through this comprehensive approach, we aim to advance both the theoretical understanding of dengue transmission dynamics and the practical application of machine learning for disease forecasting in complex real-world settings.

## Materials and methods

### Ethics statement

This study received an exemption from ethical review by the Ethics Committee of the Faculty of Tropical Medicine, Mahidol University, Thailand (EC Submission No. TMEC 24–068; Documentary Proof of Exemption: MUTM-EXMPT 2024–005, dated 8 July 2024). The exemption was granted as this research involved retrospective analysis of anonymized, aggregated surveillance data with no direct human subject involvement. The Ethics Committee certified that the study complies with the Declaration of Helsinki, ICH Guidelines for Good Clinical Practice, and other international guidelines for human research protection.

### Methodology workflow

This study established a framework to identify predictors of dengue risk, illustrated in S1 Fig. The framework applies machine learning to rank the most informative predictors among 54 input features spanning climatic, bioclimatic, socioeconomic, and remote-sensing data. These data were obtained from three main sources: the TerraClimate dataset, the MODIS dataset, and Thai national authorities. Gridded climatic and remote-sensing data were spatially aggregated to the provincial level; lagged variables and bioclimatic indices were then derived from monthly provincial temperature and precipitation series. Socioeconomic data were obtained directly at the provincial level: the biennially reported household income series was linearly interpolated to fill missing years, and all socioeconomic series were then upsampled to monthly resolution by assigning each year’s reported value (typically released at year-end) to every month of that year. Once the monthly feature set was constructed, four candidate machine learning models were compared in a preliminary screening step (S1 Table). XGBoost was selected based on this comparison; hyperparameters were optimized via Bayesian search with 5-fold cross-validation, and early stopping during boosting was applied using the final two years of the training period as the validation set. The optimized model was then used for performance evaluation and SHAP-based feature-importance analysis.

### Study design and data sources

#### Dengue case data.

Data used in this study are monthly dengue hemorrhagic fever (DHF) case reports obtained from the Bureau of Epidemiology, Department of Disease Control, Ministry of Public Health, Thailand, spanning January 2003 to December 2022. Case definitions followed national surveillance standards, where positive cases were identified through either clinical assessment at the point of care or laboratory confirmation at reporting hospitals. Provincial-level data were aggregated monthly, with cases from Buengkarn province incorporated into Nong Khai province after it was administratively separated from Nong Khai in 2011 for consistency. The epidemiological unit is the province, and the time step is the month.

#### Climate variables.

Climate data were extracted from the TerraClimate dataset [40], which provides high-resolution (4 km^2^) gridded climate information. We obtained six primary variables: precipitation, minimum temperature, maximum temperature, soil moisture, vapor pressure, and wind speed. These gridded data were spatially averaged to the provincial level to match our epidemiological units. Additionally, we derived 19 bioclimatic indicators from monthly temperature and precipitation data using the R package ‘dismo’ [41,42]. These bioclimatic variables capture annual trends, seasonality, and environmental variability patterns relevant to disease transmission.

#### Socioeconomic indicators.

We incorporated three socioeconomic factors to account for urbanization and economic development effects on dengue transmission. Gross Provincial Product (GPP), adjusted for inflation using the Laspeyres Index, was obtained from the National Economic and Social Development Council (https://www.nesdc.go.th). Demographic data, including provincial population statistics and mean household size, were acquired from the Bureau of Registration Administration (https://stat.bora.dopa.go.th). Mean provincial household income data were obtained from the National Statistical Office of Thailand (https://www.nso.go.th), where values are reported biennially with missing years interpolated linearly. The household income series, reported biennially by the Thai authorities, was linearly interpolated using the pandas *interpolate* method to fill missing years. All socioeconomic series were then upsampled from annual to monthly resolution by assigning each year’s reported value to every month of that year, on the assumption that annual values represent conditions across the full year.

#### Remote sensing data.

Environmental conditions were assessed using Moderate Resolution Imaging Spectroradiometer (MODIS) data from the Terra satellite (MOD09A1: Surface Reflectance 8-Day L3 Global 500m SIN Grid V005). We calculated two ecological indices (MNDWI and NDVI) following our established methodology [43]. Briefly, the water index was derived using the Modified Normalized Difference Water Index (MNDWI) calculated from green and infrared band 7, expressed as the percentage of provincial pixels with MNDWI > 0. The vegetation index was calculated using the Normalized Difference Vegetation Index (NDVI) from red and near-infrared bands, expressed as the percentage of provincial pixels with NDVI values between 0.5 and 1.0. The full description and source of the variables are summarized in Table 1.

**Table 1:** Variables used in the XGBoost model for dengue risk classification in Thailand.

Index	Variable/ Feature	Temporal scale	Data Format	Feature description (Unit)	Sources
	**Climate**				
1	ppt	Monthly	Float	Monthly average of precipitation (mm)	TerraClimate (https://www.climatologylab.org/terraclimate.html)
2	tmin	Monthly	Float	Monthly average of minimum temperature (°C)	
3	tmax	Monthly	Float	Monthly average of maximum temperature (°C)	
4	vap	Monthly	Float	Monthly average of vapor pressure (kPa)	
5	soil	Monthly	Float	Monthly average of soil moisture (mm)	
6	ws	Monthly	Float	Monthly average of wind speed (m/s)	
7-9	ppt-n; n = 1,2,3	Monthly	Float	ppt from n month/s prior (mm)	Calculated using above
10-12	tmin-n; n = 1,2,3	Monthly	Float	tmin from n month/s prior (°C)	
13-15	tmax-n; n = 1,2,3	Monthly	Float	tmax from n month/s prior (°C)	
16-18	vap-n; n = 1,2,3	Monthly	Float	vap from n month/s prior (kPa)	
19-21	soil-n; n = 1,2,3	Monthly	Float	soil from n month/s prior (mm)	
22-24	ws-n; n = 1,2,3	Monthly	Float	ws from n month/s prior (m/s)	
	**Bioclimatic**			**Temperature-related bio1-bio11**	
25	bio1	Yearly	Float	Mean annual temperature	https://rdrr.io/cran/dismo/man/biovars.html [42]
26	bio2	Yearly	Float	Mean diurnal range (mean of max temp - min temp)	
27	bio3	Yearly	Float	Isothermality (bio2/bio7) (* 100)	
28	bio4	Yearly	Float	Temperature seasonality (standard deviation *100)	
29	bio5	Yearly	Float	Max temperature of the warmest month	
30	bio6	Yearly	Float	Min temperature of the coldest month	
31	bio7	Yearly	Float	Temperature annual range (bio5-bio6)	
32	bio8	Yearly	Float	Mean temperature of the wettest quarter	
33	bio9	Yearly	Float	Mean temperature of driest quarter	
34	bio10	Yearly	Float	Mean temperature of warmest quarter	
35	bio11	Yearly	Float	Mean temperature of coldest quarter	
				**Precipitation-related bio12-bio19**	
36	bio12	Yearly	Float	Total (annual) precipitation	
37	bio13	Yearly	Float	Precipitation of wettest month	
38	bio14	Yearly	Float	Precipitation of driest month	
39	bio15	Yearly	Float	Precipitation seasonality (coefficient of variation)	
40	bio16	Yearly	Float	Precipitation of wettest quarter	
41	bio17	Yearly	Float	Precipitation of driest quarter	
42	bio18	Yearly	Float	Precipitation of warmest quarter	
43	bio19	Yearly	Float	Precipitation of coldest quarter	
	**Socioeconomic**				
44	GPP	Yearly	Float	Gross provincial product (million baht)	https://www.nesdc.go.th/nesdb_en/more_news.php?cid=156
45	HouseholdSize	Yearly	Float	Mean household size (person)	https://stat.bora.dopa.go.th/new_stat/webPage/statByYear.php
46	income	Yearly	Float	Mean household income (baht)	https://www.nso.go.th/nsoweb/nso/statistics_and_indicators?impt_branch=309
	**Remote sensing**				
47	per.mndwi	Monthly	Float	Water index: Percentage of area with an MNDWI value greater than or equal to zero for each month	Terra Surface Reflectance 8-Day L3 Global 500 m SIN Grid V005 (MOD09A1)
48	per.ndvi	Monthly	Float	Vegetation index: Percentage of NDVI pixels that are within 0.5 to 1 for each month	
49-51	per.mndwi-n; n = 1,2,3	Monthly	Float	Water index from n month/s prior	
52-54	per.ndvi-n; n = 1,2,3	Monthly	Float	Vegetation index from n month/s prior	
	**Target Class**				
–	Dengue Risk (binary)	Monthly	Integer	Province with higher or equal (1) and lower (0) than median DHF incidence rate (reported cases per 100,000 population)	Bureau of Epidemiology, Thailand

### Data processing and feature engineering

#### Temporal partitioning.

We divided the dataset into training (2003–2016, 14 years) and testing (2017–2022, 6 years) periods. This split provided sufficient training data to capture inter-annual variability, including multiple dengue epidemic cycles, while reserving recent years for independent validation. Within the training set, we used the final two years (2015–2016) as a validation set for early stopping to prevent overfitting during model optimization. The testing period was further stratified into three distinct epidemiological phases: pre-COVID-19 (2017–2019), representing baseline model performance under typical transmission conditions; COVID-19 (2020–2021), capturing the period of active pandemic restrictions and interventions; and post-COVID-19 (2022), assessing whether model performance recovered following the relaxation of pandemic measures.

#### Risk classification.

Our objective was to develop an operationally relevant early warning system that flags province-months as high versus low dengue risk, rather than predicting exact case counts. Binary classification offers several methodological advantages in this epidemiological context, including providing actionable decision boundaries for public health surveillance and resource allocation. Additionally, binary classification is more robust to systematic reporting biases and temporal non-stationarity than count-based regression models.

We calculated monthly DHF incidence rates per 100,000 population for each province. Using the national median incidence from the training period (2.78 per 100,000) as our classification threshold, we labeled each province-month observation as high-risk (≥ median) or low-risk (<median). This median-based approach ensures balanced target classes (approximately 50% in each category), thereby optimizing machine learning performance and preventing bias toward the majority class. We employed stratified 5-fold cross-validation to maintain this class balance across all training folds, further stabilizing the learning process.

#### Lagged variables.

To capture delayed environmental effects on dengue transmission, we generated 1-, 2-, and 3-month lagged values for all climate and remote sensing variables, expanding our feature set to account for temporal dependencies in disease dynamics.

### Machine learning approach

#### XGBoost model implementation.

We employed a supervised machine learning approach to capture intricate feature interactions and classify each province-month as high- or low-risk based on the input features. To identify a suitable algorithm, we first conducted a preliminary comparison in which all 54 features were supplied to four classification models — logistic regression, support vector machine (SVM), random forest, and XGBoost (S1 Table). Performance was assessed across six metrics on the training set and both testing periods; XGBoost outperformed the other algorithms across all settings and was therefore selected for downstream analysis. XGBoost constructs an ensemble of decision trees through gradient boosting, combined with an approximate tree learning technique that scales efficiently to large feature sets [44]. The XGBoost algorithms work based on additive training, where a new tree is constructed to correct the errors made by the previous trees. The new tree was iteratively created to minimize the errors until the errors could not be reduced further, or a specific stop condition was met (see S1 Text for more details on the mathematical description of XGBoost).

Unlike linear or distance-based classifiers, decision-tree ensembles such as XGBoost are invariant to monotonic transformations of individual features, since splits depend on the relative ordering of feature values rather than their magnitudes. We therefore did not standardize or rescale inputs prior to training XGBoost. For comparability, the logistic regression and support vector machine baselines were trained on Z-score-transformed features, as both are sensitive to feature scale. We also did not detrend or deseasonalize the input features: XGBoost does not require the strict stationarity assumed by traditional time-series models such as ARIMA or SARIMA. The autocorrelation analysis of model residuals did reveal significant short-lag autocorrelation, indicating that the model leaves some month-to-month temporal dependence unexplained. However, because the goal of this study is to identify environmental and socioeconomic predictors through feature-importance analysis rather than to produce point forecasts, this residual dependence is unlikely to materially affect our main conclusions; a sensitivity analysis that adds a single autoregressive predictor removed the residual autocorrelation while leaving temperature as the leading environmental driver (see Results and Discussion).

#### Model interpretability.

To address the inherent complexity of XGBoost feature interactions, we implemented SHapley Additive exPlanations (SHAP) [45]. SHAP quantifies each feature’s contribution to individual predictions using cooperative game theory principles. This approach decomposes model predictions into additive feature contributions, enabling both global feature importance ranking and local prediction explanations.

#### Hyperparameter optimization.

We optimized model hyperparameters using Bayesian optimization with the Tree-Structured Parzen Estimator (TPE) algorithm implemented in the Python package ‘Optuna’ [46]. TPE employs performance-aware sampling, concentrating the search in promising hyperparameter regions. The optimization process targeted the number of trees, learning rate, maximum tree depth, subsampling ratio, column sampling by tree, minimum child weight, and regularization parameters (gamma, alpha, lambda). We used 5-fold stratified cross-validation on the training set, preserving class balance in each fold. The optimization objective was to minimize log-loss, with the optimal hyperparameter set selected based on average cross-validation performance. We additionally introduced the early-stopping mechanism to further prevent overfitting the boosted tree to the training set. In this work, we set this early stopping round to 30, using the last two years of the training set as the validation set for the early stopping process.

#### Performance metric.

To measure XGBoost models’ performance, an evaluation metric for classification was used. The Area Under the receiver operating characteristic Curve (AUC) was selected to measure the model performance. AUC is designed to measure the total predictive power of the model regardless of the classification threshold. By adjusting the classification threshold, one can observe the tradeoff between two key metrics: true positive rate (sensitivity) and false positive rate (1-specificity). The threshold-dependent metrics were also utilized to additionally measure model performance on XGBoost prediction. The prediction probabilities of XGBoost are transformed to high-risk (class 1) and low-risk (class 0) predictions based on whether the probabilities are higher or lower than the selected threshold, respectively. Four metrics were chosen as follows:


$$\begin{array}{c} \text{Accuracy }=\;\frac{TP\;+TN}{TP+TN+FP+FN}, \end{array}$$ (1)

$$\begin{array}{c} \text{Precision }=\;\frac{TP\;}{TP+FP}, \end{array}$$ (2)

$$\begin{array}{c} \text{Sensitivity}=\;\frac{TP}{TP+FN}, \end{array}$$ (3)

$$\begin{array}{c} \text{F1}-\text{score }=\frac{\text{2}}{{Sensitivity}^{-\text{1}}+Precision^{-\text{1}}}=\;\frac{\text{2}\;TP}{\text{2}\;TP+FP+FN}, \end{array}$$ (4)

where *TP* is the number of true positive predictions, *TN* is the number of true negative predictions, *FP* is the number of false positive predictions, and *FN* is the number of false negative predictions.

#### Implementation details.

All analyses were conducted using Python 3.10 with the following key packages: pandas for data manipulation, numpy for numerical operations, scikit-learn for preprocessing and evaluation, XGBoost for model training, and SHAP for interpretability analysis.

## Results

### Spatiotemporal patterns of dengue hemorrhagic fever in Thailand

**Fig 1:**
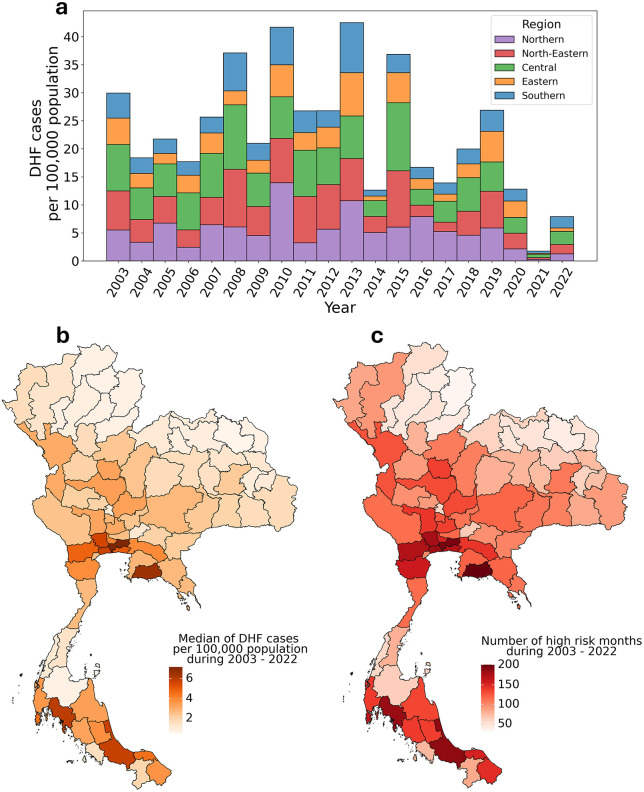
Spatiotemporal distribution of dengue hemorrhagic fever (DHF) in Thailand, 2003–2022. **(a)** Annual DHF incidence rates by region, showing temporal trends across Thailand’s five major administrative regions. **(b)** Geographic distribution of median provincial DHF incidence rates over the study period, with color intensity corresponding to disease burden (darker shades indicate higher incidence per 100,000 population). **(c)** Cumulative number of high-risk months by province during 2003 - 2022, where high risk is defined as monthly incidence exceeding the national median threshold of 2.78 per 100,000 population (derived from 2003–2016 training data). Provinces with higher values indicate persistent dengue hotspots. The map plots were created using the plotnine package in Python with a shapefile from The Humanitarian Data Exchange (HDX), *Thailand – Subnational Administrative Boundaries* (source: Royal Thai Survey Department, provided by OCHA; https://data.humdata.org/dataset/cod-ab-tha), licensed under the Creative Commons Attribution for Intergovernmental Organisations license (CC BY-IGO 3.0, http://creativecommons.org/licenses/by/3.0/igo/legalcode).

Throughout the 20-year study period (2003–2022), Thailand documented 681,430 dengue hemorrhagic fever (DHF) cases, exhibiting substantial spatiotemporal heterogeneity (Fig 1a). Monthly case counts demonstrated dramatic variation, ranging from a maximum of 13,601 cases in July 2013 to a minimum of 105 cases in February 2022. The seasonal distribution revealed pronounced periodicity, with peak transmission occurring during the early wet season, specifically in June (13.16%), July (14.96%), and August (13.98%), totaling 42.12%. In contrast, February consistently showed the lowest disease burden (3.71%) (S2 Fig). Among Thailand’s administrative regions, the southern provinces experienced the highest regional incidence rate, reaching 13.93 per 100,000 population in 2010. Notably, the years 2014 and 2016 exhibited markedly reduced DHF incidence compared to other years within the training period, suggesting the potential influence of climatic anomalies or intensified control measures.

Geographic analysis identified persistent high-risk zones predominantly concentrated in central and southern Thailand (Fig 1b). Utilizing the national median incidence rate of 2.78 cases per 100,000 population as a binary classification threshold, we delineated provinces with sustained elevated dengue burden. The cumulative frequency analysis of high-risk months revealed that certain provinces maintained endemic transmission for extended periods (Fig 1c). This epidemiological threshold subsequently served to categorize each province-month observation as either high-risk or low-risk, establishing the target classes for our machine learning classification framework.

### Model performance and validation

**Fig 2:**
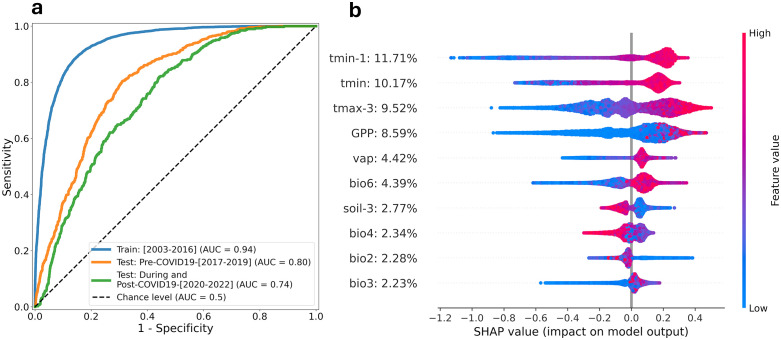
XGBoost model performance and feature importance analysis. **(a)** Receiver Operating Characteristic (ROC) curves comparing model discrimination across training (2003–2016) and test datasets (2017–2022), with Area Under the Curve (AUC) values indicating predictive performance. The diagonal reference line represents random classification (AUC = 0.5). Model hyperparameters were optimized using 5-fold cross-validation. **(b)** SHAP feature importance analysis showing the top 10 predictive variables ranked by mean absolute SHAP values, based on 2003–2019 data. Percentages indicate each feature’s relative contribution among all 54 input features. Each dot represents an individual observation, colored by feature value (red = high, blue = low). Horizontal position indicates the feature’s impact on predicted dengue risk, with positive SHAP values increasing and negative values decreasing the probability of high-risk classification.

The XGBoost model achieved an AUC of 0.94 on the training dataset (2003–2016), confirming that the optimized model fit the in-sample data well. On out-of-sample data, the model maintained strong discriminative performance during the pre-COVID-19 testing period (2017–2019), with an AUC of 0.80. However, performance deteriorated markedly during and following the COVID-19 pandemic (2020–2022), declining to an AUC of 0.74 (Fig 2a). Complementary threshold-dependent metrics, including accuracy, precision, sensitivity, and F1-score, corroborated the model’s ability to effectively discriminate between high and low-risk provinces throughout the pre-pandemic period (2003–2019) (S3 Fig). Furthermore, we calculated brier score of each dataset as follows: training: 0.116, pre-COVID-19 testing: 0.198 and during and post-COVID-19: 0.282. This shows that the model predicted probability is still acceptable in non-COVID-19 testing period while the during and post COVID-19 period model probability shows poor predictive performance.

**Fig 3:**
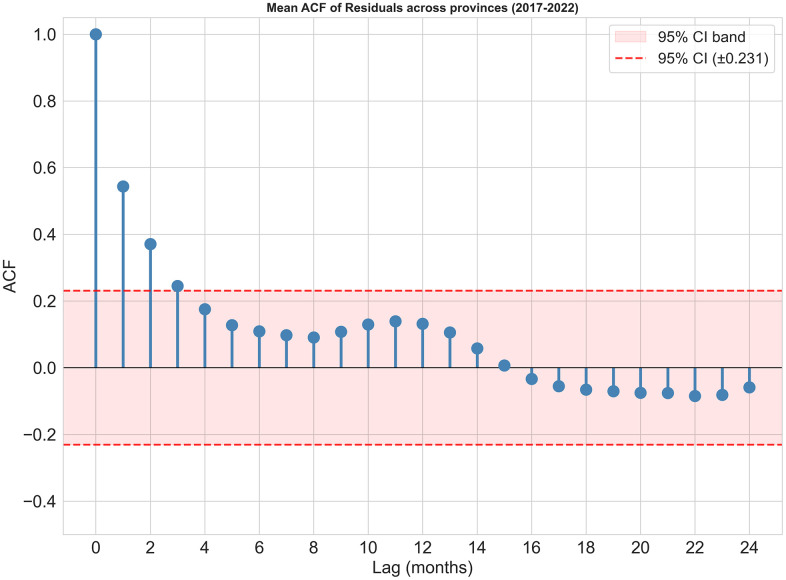
Mean autocorrelation function (ACF) of model residuals across Thai provinces, testing period (2017–2022). Residuals were computed as the difference between predicted and observed probabilities of high-risk classification for each province-month. The ACF was calculated separately for each province at each lag and then averaged across the 77 provinces to summarize the country-level temporal structure. The red shaded region denotes the 95% confidence band under the null hypothesis of no autocorrelation ($\pm \frac{\text{1}.\text{9}\text{6}}{\sqrt{T}}$, with *T* = 72 months per province). Mean residual ACF values exceed the band at lags 1–3, indicating significant short-range positive autocorrelation in the residuals.

We further examined whether the model captured the temporal structure of the data by computing the autocorrelation function (ACF) of the residuals (the difference between predicted and observed probability of high-risk classification). Residuals were calculated separately for each province over the testing period, and the per-province ACFs were averaged across provinces at each lag to summarize the country-level temporal structure (Fig 3). The mean residual ACF showed significant positive autocorrelation at short lags (lag-1 = 0.54, lag-2 = 0.37, lag-3 = 0.24), exceeding the 95% confidence band (±0.23) through lag 3 and falling within the band at longer lags. This indicates that the residuals retain short-range (month-to-month) temporal structure that the contemporaneous and lagged environmental and socioeconomic features do not fully capture. To identify the source of this autocorrelation and assess whether it affects our conclusions, we performed a sensitivity analysis (S2 Text). This analysis showed that including a single autoregressive predictor (the previous month’s observed high-risk status) removed the residual autocorrelation (no lag exceeded the 95% confidence band) and improved out-of-sample discrimination, while temperature remained the leading environmental driver.

**Fig 4:**
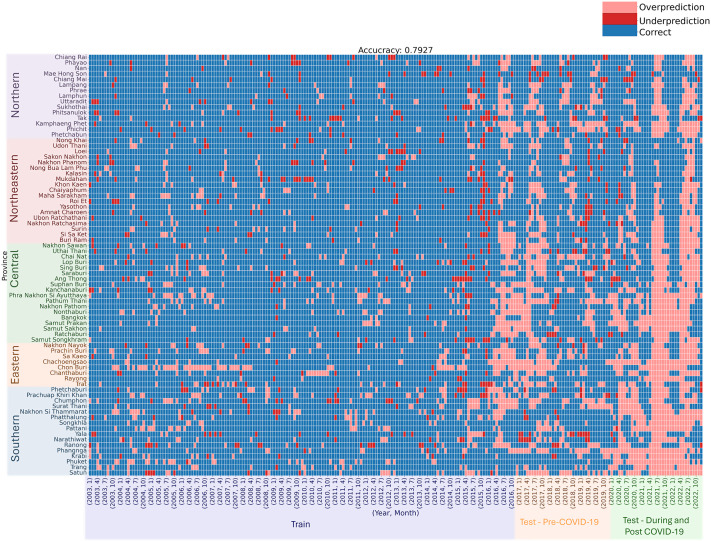
Spatiotemporal evaluation of XGBoost model predictions across Thai provinces, 2003–2022. The heatmap displays monthly prediction accuracy for each province throughout training (2003–2016) and testing (2017–2022) periods. Colors indicate prediction outcomes: correct predictions (blue), false positives/overprediction (pink; high risk predicted when actual risk was low), and false negatives/underprediction (red; low risk predicted when actual risk was high); the ground truth is shown in S4 Fig. Provinces are grouped by region and ordered from north to south by latitude. The overall model accuracy is 79.27%.

Temporal stratification of model predictions revealed nuanced performance patterns. While the model could accurately capture dengue risk dynamics prior to 2017, a systematic tendency toward overprediction emerged during 2017–2019 (Fig 4). This overprediction pattern suggests the presence of factors influencing dengue transmission that were not captured by our environmental and socioeconomic predictors, such as human mobility patterns [47–50]. The overprediction bias intensified dramatically following 2020, coinciding with the onset of the COVID-19 pandemic restrictions [36,47,35]. However, despite these temporal variations in performance, the model maintained an overall accuracy of 79.27% across the entire 20-year study period.

### Feature importance and interaction analysis

SHAP analysis revealed temperature variables as the dominant predictive features, with remarkable consistency across multiple metrics. Among the 54 input features, temperature-related variables occupied seven of the ten highest-ranked positions based on mean absolute SHAP values (Fig 2b). Despite high correlations among temperature-related features (S5 Fig), XGBoost’s tree-based algorithm effectively handled this multicollinearity by selecting the most informative predictors at each split [51,52]. The two most influential predictors were 1-month lagged minimum temperature (tmin-1) and current-month minimum temperature (tmin), underscoring the critical importance of minimum thermal conditions — at both immediate and one-month-lagged timescales — in dengue transmission dynamics. Three-month lagged maximum temperature (tmax-3) ranked third, reinforcing the combined influence of antecedent and contemporaneous temperature effects on disease risk.

All temperature variables exhibited positive associations with dengue risk across all temporal scales, reinforcing the established biological relationship between elevated temperatures and enhanced dengue transmission potential. Gross provincial product (GPP) emerged as the fourth most influential predictor, with higher economic development associated with increased dengue risk, which reflects the predominantly urban nature of dengue transmission in Thailand. The top ten features collectively accounted for 58.42% of the model’s total predictive capacity. Among these leading predictors, four bioclimatic indices—namely, minimum temperature of the coldest month (bio6), temperature seasonality (bio4), mean diurnal range (bio2), and isothermality (bio3)—were all temperature-derived metrics. In marked contrast, precipitation variables, precipitation-related bioclimatic indices, and remote sensing indicators contributed minimally to model predictions.

**Fig 5:**
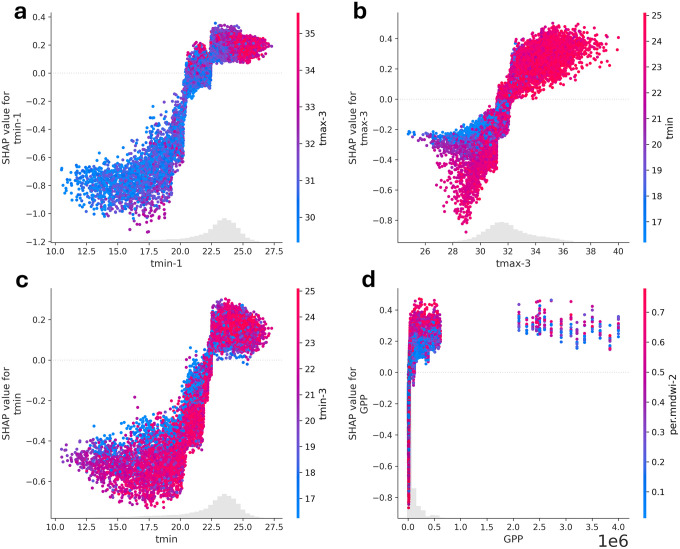
SHAP dependence plots for the four most important predictive features. Each dot represents a single observation (province-month), with the x-axis showing the feature value and the y-axis showing its SHAP value (contribution to predicted log odds). Gray shading indicates the feature distribution across the dataset (2003–2019). Dot color represents the value of the most strongly interacting feature, as determined by SHAP’s interaction analysis, revealing how feature combinations influence dengue risk predictions. Positive SHAP values indicate increased predicted risk, while negative values indicate decreased risk.

SHAP dependence plots illuminated complex nonlinear relationships and significant feature interactions governing dengue risk (Fig 5). The relationship between 1-month lagged minimum temperature (tmin-1) and dengue risk exhibited a distinct threshold effect: temperatures below approximately 21°C were negatively associated with transmission risk, while the positive effect reached a plateau at approximately 24°C. This relationship was substantially modulated by 3-month lagged maximum temperature, with elevated tmax-3 values amplifying the effect of tmin-1 on predicted risk (Fig 5a). Similarly, tmax-3 demonstrated a nonlinear response with a critical threshold at approximately 32°C, beyond which dengue risk increased markedly. This temperature effect was modified by concurrent minimum temperature, with lower tmin values likely attenuating the positive influence of tmax-3, particularly within the 28–31°C range (Fig 5b).

The interaction between current and 3-month lagged minimum temperatures revealed a biologically significant threshold at approximately 22°C, potentially representing a fundamental constraint for sustained dengue virus transmission (Fig 5c). The 2-month lagged water index (per.mndwi-2) likely exhibited synergistic interactions with GPP, particularly pronounced in provinces where GPP values remained below 10^6^ million baht. Conversely, this amplifying effect diminished substantially in Bangkok, where GPP values (> 2 x 10^6^ million baht) exceeded those of all other provinces (Fig 5d).

### Impact of the COVID-19 pandemic on dengue dynamics

**Fig 6:**
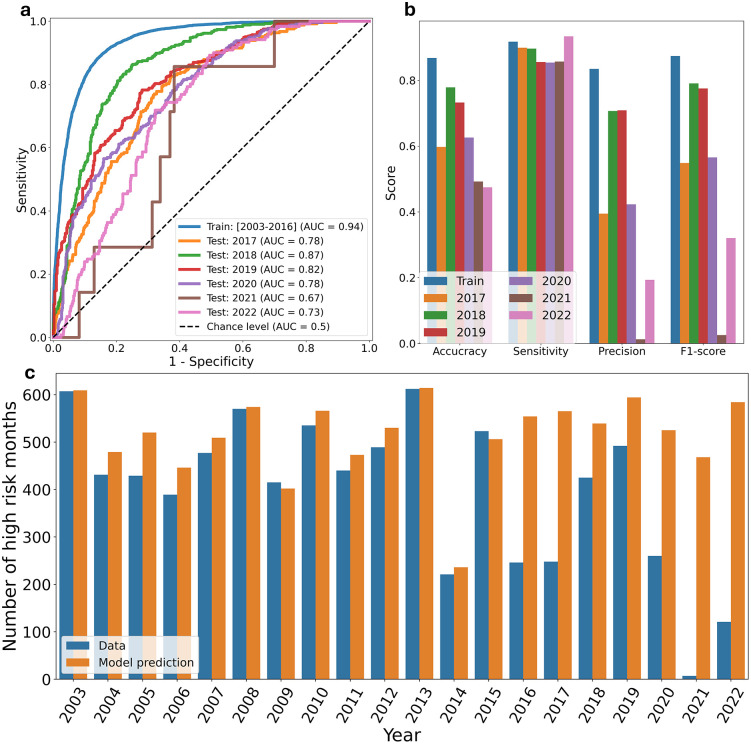
Impact of COVID-19 on model performance across three periods: pre-pandemic (2003–2019), pandemic (2020–2021), and post-pandemic (2022). **(a)** Receiver Operating Characteristic (ROC) curves showing declining model discrimination ability across periods, with AUC values dropping from pre-pandemic baseline. The diagonal line indicates random classification (AUC = 0.5). **(b)** Annual performance metrics (accuracy, precision, sensitivity, and F1-score) calculated using a 0.5 classification threshold, revealing substantial performance degradation during and after the pandemic. **(c)** Comparison of predicted versus observed high-risk province-months by period, demonstrating systematic model overprediction during the pandemic when dengue transmission was suppressed.

The COVID-19 pandemic fundamentally disrupted dengue transmission patterns [35], resulting in precipitous declines in model performance. Annual AUC reached its lowest at 0.62 in 2021, representing the poorest discriminative capacity observed throughout the entire study period (Fig 6a). This performance degradation persisted into the post-pandemic period, suggesting enduring alterations to dengue epidemiological dynamics. All performance metrics exhibited consistent deterioration during this period, confirming the comprehensive nature of the disruption (Fig 6b).

The model’s overprediction during the pandemic was substantial. Employing a default classification threshold of 0.5, the model predicted 545, 464, and 581 high-risk province-months for 2020, 2021, and 2022, respectively. In contrast, observed data revealed only 260, 7, and 121 actual high-risk instances during these corresponding periods (Fig 6c). This systematic overestimation indicates that pandemic-related factors, including mobility restrictions, behavioral modifications, and altered healthcare-seeking patterns, suppressed dengue transmission through mechanisms operating independently of climatic drivers [35,53]. The persistence of this suppression effect into 2022 suggests that dengue transmission dynamics might have undergone fundamental restructuring, necessitating comprehensive model recalibration to restore predictive accuracy.

## Discussion

Our study demonstrates the potential of machine learning, particularly eXtreme Gradient Boosting (XGBoost), for classifying dengue hemorrhagic fever risk across Thailand by integrating climatic, environmental, and socioeconomic variables. The model achieved an AUC of 0.80 in pre-pandemic testing (2017–2019) and 0.74 in the combined during- and post-pandemic period (2020–2022), and identified temperature-related variables as the predominant predictors of dengue transmission risk. The marked disruption of model performance during the COVID-19 pandemic suggests the complex interplay between environmental drivers and other factors not captured in our model — including human mobility, control measures, and healthcare-seeking behavior — that govern dengue epidemiology in Thailand.

Brier scores corroborate the AUC pattern: 0.116 on training, 0.198 in the pre-COVID-19 testing period, and 0.282 during and after the pandemic. The pre-pandemic value indicates well-calibrated probabilities, consistent with the AUC of 0.80 in the same period. The pandemic-era Brier, while only modestly higher in absolute terms, reflects a substantive failure of calibration: the empirical positive rate collapsed (only 7 high-risk province-months in 2021, S4 Fig), yet the model’s probability outputs remained anchored to the pre-pandemic base rate, producing the systematic overprediction. The model’s predicted probabilities should accordingly not be used for public-health decision-making during periods of major epidemiological disruption without first recalibrating to the prevailing base rate.

### Temperature as the primary predictor of dengue risk

Our analysis indicates that temperature is the most influential environmental determinant of dengue risk throughout Thailand, with temperature-related variables comprising seven of the ten most important predictive features. This finding aligns with well-established biological mechanisms governing dengue virus transmission dynamics [22,54]. Temperature exerts multifaceted effects on the transmission cycle, modulating the extrinsic incubation period of the virus within mosquito vectors, accelerating mosquito developmental rates, and increasing biting frequency within specific thermal ranges [54,55].

The threshold effect we identified at approximately 21°C for minimum temperature corresponds closely with established biological constraints on *Aedes aegypti*–mediated dengue transmission. Several parameters governing mosquito behavior — including biting rate, adult lifespan, and developmental rate — drop sharply below approximately 21°C compared with their optima in the 25–30°C range [22,54]. Below this threshold, viral replication within *Ae. aegypti* is also substantially reduced [22,54]. Complementary experimental work on *Ae. albopictus* (a secondary dengue vector) places the lower dissemination limit at approximately 18°C, at which DENV-2 establishes midgut infection but does not reach the salivary glands [56].

Direct experimental evidence for a thermal threshold on dengue transmission comes from Watts et al. [57], who fed *Ae. aegypti* on DEN-2-viremic rhesus monkeys and then incubated the engorged mosquitoes at constant temperatures ranging from 20 to 35°C before allowing them to transmit to susceptible recipients. At 20°C, virus established in the mosquito midgut but failed to reach the salivary glands within a 25-day observation window. At 24°C and 26°C, salivary-gland infection eventually developed but transmission to recipient monkeys still did not occur, even when the infecting viral dose was increased — implying that salivary-gland viral titres at these temperatures remained below the threshold required to infect a host via the bite. Transmission occurred only at 30°C and above, and the extrinsic incubation period (EIP) shortened steeply with temperature, from 25 days at 30°C with a low infecting dose, to 12 days at 30°C with a higher dose, and to 7 days at 32°C and 35°C. Together these findings indicate that low ambient temperatures act as a biological barrier to dengue transmission through two related mechanisms — failure of viral dissemination from the midgut at the coolest temperatures, and insufficient salivary-gland viral titres at intermediate temperatures — and that warmer conditions accelerate the EIP, expanding the temporal window in which infected mosquitoes can transmit before dying.

The analysis revealed that the 3-month lagged maximum temperature (tmax-3) exhibited a distinct critical threshold near 32°C. Below this threshold, the effect on dengue risk remained minimal; however, risk increased substantially once monthly maximum temperatures exceeded this point. This threshold likely represents the optimal temperature range where both mosquito development rates and virus replication achieve maximum efficiency [22,54,58]. Interestingly, the plateau effect observed for maximum temperature was less pronounced compared to that of minimum temperature. This asymmetry suggests that while extremely high maximum temperatures may limit transmission through increased mosquito mortality or reduced virus transmission probability, these constraining effects are less significant than those imposed by lower temperature thresholds [59]. Such asymmetric temperature effects carry important implications for understanding climate change impacts on dengue transmission, as warming trends may expand suitable transmission zones primarily through elevated minimum temperatures rather than through alterations in maximum temperature patterns [60].

The temporal dynamics of dengue transmission are effectively captured by the prominence of lagged temperature variables, particularly 1-month lagged minimum temperature (tmin-1) and 3-month lagged maximum temperature (tmax-3). The 1-month lag for minimum temperature likely reflects immediate effects on adult mosquito survival and viral replication efficiency, whereas the 3-month lag for maximum temperature may represent cumulative impacts on mosquito population dynamics and the temporal requirements for epidemic establishment following favorable environmental conditions [22,61,62]. These observations align with previous research demonstrating that temperature effects on dengue transmission operate across multiple temporal scales, influencing both immediate vector competence and longer-term population dynamics [22,61–63].

Our results indicate that precipitation variables played a much smaller role in model predictions than expected, with the 1-month lagged precipitation variable ranking 11^th^ out of 54 considered factors. While this finding contradicts the traditional emphasis on rainfall in dengue prediction models, it aligns with emerging evidence suggesting that precipitation-dengue relationships are highly context-dependent and often non-monotonic and non-linear [64]. In Thailand’s urban environments, artificial water containers might provide year-round breeding sites that would remain independent of natural rainfall patterns [65]. In rural areas, the relationship is equally complex: once precipitation reaches a certain threshold, additional rainfall might only slightly contribute to dengue risk. In fact, Thailand experiences higher average rainfall in August and September than in July, yet has fewer dengue cases (S2 and S6 Figs). This might be due to excessive rainfall that can flush larvae from breeding containers, thereby reducing vector populations. In contrast, drought conditions may lead to increased household water storage practices, paradoxically creating more breeding opportunities within households [55,66,67]. This complexity underscores the limitations of simplistic rainfall-based prediction approaches and highlights the need for more nuanced environmental indicators in dengue forecasting models in Thailand.

### Socioeconomic factors and urbanization

The emergence of Gross Provincial Product (GPP) as the fourth most influential predictor reveals a seemingly paradoxical relationship wherein higher economic development correlates with increased dengue risk. This relationship illuminates the fundamentally urban nature of dengue transmission within Thailand. The urbanization process might create optimal conditions for *Aedes aegypti* proliferation through multiple mechanisms: the proliferation of artificial water containers that serve as all-year-round breeding sites, increased human population density and mobility that facilitates virus transmission, and urban heat island effects that maintain temperatures within favorable ranges for vector survival and development [64,68–70].

The detected interaction between GPP and environmental variables, particularly the water index, indicates that urbanization fundamentally modifies the relationship between environmental conditions and dengue risk. This modification likely occurs through alterations in mosquito breeding site availability and human behavioral patterns that influence vector-human contact rates [55,64]. These findings underscore the importance of considering socioeconomic development patterns when designing dengue control strategies in rapidly urbanizing regions.

### Impact of the COVID-19 pandemic

The decline in model performance during the pandemic period — with AUC falling to 0.62 in 2021 — points to the important role of behavioral and surveillance factors in shaping observed dengue dynamics, none of which were represented among our environmental and socioeconomic predictors. Human mobility in Bangkok changed substantially during this period in response to government interventions and shifts in everyday behavior. For a vector-borne pathogen, a central driver of secondary transmission (captured by the basic reproductive number R_0_) is the rate of vector–human contact, principally mosquito biting frequency, which depends on overlap between human activity and vector habitat [71]. Pandemic restrictions plausibly disrupted this overlap by shifting human activity patterns indoors and reducing the spatial range of host movement, thereby lowering effective biting exposure even where vector populations were unchanged.

Beyond mobility, healthcare-seeking behavior was also altered during the pandemic. Concerns about nosocomial COVID-19 transmission, hospital access restrictions, and the diversion of clinical and laboratory capacity reduced the probability that febrile dengue patients were assessed, confirmed, and reported [35, 47 -50,72]. Observed reductions in DHF therefore reflect both declines in transmission and reductions in case ascertainment. Both mechanisms, mobility-driven changes in exposure and surveillance disruption, operate on dimensions of transmission orthogonal to our climatic and socioeconomic predictors, and each would be expected to produce the systematic overprediction observed during 2020–2022.

The persistence of reduced model performance extending into 2022 suggests potential long-term alterations in dengue epidemiology. These changes might encompass shifts in population immunity patterns and modifications in vector population dynamics. More broadly, purely environmental models, however well-calibrated under stationary conditions, can fail substantially during periods of major societal disruption [35,72]. Future modeling efforts should consider incorporating real-time mobility data, healthcare utilization patterns, and other behavioral indicators to maintain predictive accuracy during unprecedented circumstances.

### Limitations

Several limitations warrant careful consideration when interpreting our findings. First, our dependence on passive surveillance data likely results in systematic underestimation of true dengue incidence, particularly in regions with limited healthcare access or reduced healthcare-seeking behavior [39]. The exclusive use of reported DHF cases rather than comprehensive dengue case data may introduce bias toward more severe disease patterns, potentially limiting the generalizability of our predictions to mild or asymptomatic infections. Second, the absence of direct measures of vector abundance, human mobility, and immunity patterns limits our understanding of transmission dynamics. Third, the effects of dengue control measures were not considered in this study, which might interfere with the dynamics of dengue transmission. Fourth, our binary classification may achieve high accuracy partly by identifying provinces with consistent endemic levels rather than temporal variations. However, since our primary objective was identifying environmental and socioeconomic drivers rather than spatiotemporal outbreak prediction, this limitation does not undermine our key findings. Fifth, our binary risk classification, while effective, may oversimplify the complex risk landscape. A multiclass approach could provide more nuanced risk stratification and deeper insights into how climatic and socioeconomic factors influence disease patterns. To confirm that these findings are not specific to the median cut-point, we repeated the analysis using a more stringent 75th-percentile incidence threshold (6.08 per 100,000); test-period discrimination (ROC-AUC 0.79 pre-COVID-19; 0.72 during/post-COVID-19) and the SHAP-based dominance of temperature (six of the ten leading features) were mainly preserved (S3 Text, S7 Fig, S2 Table). Sixth, the model’s reduced performance during the COVID-19 pandemic highlights its vulnerability to unprecedented societal changes not captured in the training data. Finally, the residual autocorrelation analysis revealed significant short-lag (1–3 month) positive autocorrelation, reflecting our deliberate exclusion of autoregressive terms so that feature importance would capture environmental and socioeconomic drivers rather than the serial dependence of the outcome itself (see Results and S2 Text). Consequently, the model’s residuals should not be treated as temporally independent, and operational month-ahead forecasting would benefit from an autoregressive structure or an explicit temporal error model.

## Conclusions

Using XGBoost with SHAP interpretability, we identified environmental and socioeconomic predictors of provincial-level dengue risk across Thailand over a 20-year period (pre-pandemic testing AUC = 0.80; during- and post-pandemic testing AUC = 0.74). Temperature emerged as the dominant signal, comprising seven of the ten most influential features, with non-linear threshold effects near 21°C for 1-month lagged minimum temperature and 32°C for 3-month lagged maximum temperature. The prominence of 1- and 3-month temperature lags suggests that thermal history rather than current conditions shapes dengue risk in Thailand, consistent with the temperature-dependence of the extrinsic incubation period and vector demography described in the experimental literature. Higher Gross Provincial Product was associated with elevated risk, consistent with the predominantly urban character of dengue transmission, though we caution that GPP also tracks population density, healthcare infrastructure, and reporting coverage and should not be interpreted as a pure urbanization signal.

The COVID-19 period exposed important limitations of environmentally driven prediction. Discrimination dropped to AUC = 0.62 in 2021 and the model systematically overpredicted risk. We accordingly recommend that the model’s predicted probabilities not be used for public-health decision-making during periods of major epidemiological disruption without first recalibrating the model. More broadly, our findings suggest that the next generation of dengue prediction systems will need to combine environmental signals with real-time indicators of mobility, vector populations, and surveillance coverage to remain reliable through periods of societal change.

## Supporting information

S1 Text Mathematical description of XGBoost. (PDF)

S2 Text Residual autocorrelation: autoregressive sensitivity analysis. (PDF)

S3 Text Sensitivity of results to the risk-classification threshold. (PDF)

S1 Fig Methodology workflow for the dengue risk classification framework. Solid line shows the flow between stages, and dotted arrow shows results from each analysis.
(TIF)

S2 Fig Seasonal distribution of dengue hemorrhagic fever cases in Thailand, 2003–2022. Monthly case counts are shown as percentages of total reported cases, revealing peak transmission during the early rainy season (July) and lowest transmission in the dry season (February). Descriptive statistics for the monthly case counts across the study period are as follows: mean: 37.36 (SD = 87.90), minimum = 0, and maximum = 4595.
(TIF)

S3 Fig Model performance sensitivity to classification threshold, 2003–2019. Four performance metrics (accuracy, precision, sensitivity, and F1-score) are evaluated across probability thresholds from 0 to 1. The threshold determines the predicted probability cutoff for classifying provinces as high-risk versus low-risk.
(TIF)

S4 Fig Ground truth classification of dengue risk across Thai provinces, 2003–2022. Heatmap showing the actual monthly risk classification for each province based on whether DHF incidence exceeded the national median threshold (2.78 per 100,000 population). Red cells indicate high-risk months (≥ median incidence) and blue cells indicate low-risk months (<median incidence). Provinces are arranged by region and ordered from north to south. This ground truth data serves as the target variable for model training (2003–2016) and validation (2017–2022). Notable patterns include persistent high-risk status in central provinces (notably Bangkok and surrounding areas), while northern provinces show more sporadic high-risk periods. The dramatic reduction in high-risk observations during 2021–2022 reflects the impact of the COVID-19 pandemic on dengue transmission.
(TIF)

S5 Fig Correlation structure and multicollinearity among predictive features. Hierarchically clustered heatmap showing Pearson correlation coefficients between all 54 model features, with color intensity indicating correlation strength (red = negative, blue = positive).
(TIF)

S6 Fig Monthly average rainfall patterns across Thailand, 2003–2022. Bars represent mean monthly precipitation (mm) averaged across all 77 provinces over the 20-year study period. Data derived from the TerraClimate dataset. Note the peak rainfall in August-September contrasts with peak dengue transmission in July (see S2 Fig), supporting a role of precipitation in a complicated non-monotonic fashion in dengue risk prediction. Descriptive statistics for the monthly precipitation across the study period are as follows: mean: 127.43 (SD = 118.63), minimum = 0, and maximum = 1513.68.
(TIF)

S7 Fig Sensitivity of model performance and feature importance to the risk-classification threshold. Results from re-running the full pipeline with the high-risk class defined by the 75th percentile of the training-period DHF incidence (25% positive observations; *scale_pos_weight* = 3) instead of the median. **(a)** ROC curves for the training (2003–2016), pre-COVID-19 test (2017–2019), and during/post-COVID-19 test (2020–2022) periods. **(b)** SHAP summary plot of the ten most important features. **(c, d)** Precision–recall curves under the median **(c)** and 75th-percentile **(d)** thresholds. In **(c)** and **(d)**, the dashed lines denote the no-skill baseline (positive-class prevalence) for each period, color-matched to the corresponding curve; under the 75th-percentile threshold the training baseline decreases from 0.50 to 0.25, consistent with the reduced positive-class prevalence, whereas the pre-COVID-19 (0.43) and during/post-COVID-19 (0.14) baselines are essentially unchanged.
(TIF)

S1 Table Performance comparison of four machine learning models (logistic regression, support vector machine, random forest, and XGBoost,) using six metrics on training set, pre-COVID-19 (2018–2019) test set, and post-COVID-19 (2022) test set. For each metric, ranking score calculated by summation of assigned rank from best (score = 4) to worst (score = 1) for each metric. The average performance of each model was obtained from average over 10 random seeds. All models were trained on the same training set to ensure fair comparison. Hyperparameters were optimized using 5-fold cross-validation.
(PDF)

S2 Table Comparison of model performance and key feature-importance findings under the median and 75th-percentile risk-classification thresholds. AUC, area under the ROC curve.
(PDF)
