# Supplementary material for: Machine learning reveals temperature as a key predictor of dengue risk across Thailand’s provinces: A 20-year analysis
Source: PLoS Negl Trop Dis. 2026 Jul 28;20(7):e0014590. doi: 10.1371/journal.pntd.0014590 (PMC13440873; doi:10.1371/journal.pntd.0014590)
Supplement: Supplementary file 1 [file pntd.0014590.s001.pdf]

Supplementary information:

Machine learning reveals temperature as a key predictor of dengue risk across Thailand's provinces: A 20-year analysis

S1 Text: Mathematical description of XGBoost

XGBoost is a high-performance implementation of gradient-boosted decision trees, applicable to both regression and classification tasks. It uses an additive training strategy in which trees are constructed sequentially, with each new tree fit to correct the errors of the current ensemble.

The algorithm proceeds as follows:

1. Initialize with a simple prediction such as average of all targets value
2. Compute the errors caused by current tree
3. Train a small tree to predict the error
4. Add this tree's prediction to the ensemble (scaled with learning rate η)
5. Repeat 2-4 until end condition is met

The final prediction follows this equation:

$$p = F_0 + \eta F_1 + \eta F_2 + \cdots + \eta F_K$$

Where p is final prediction, η is learning rate, F_0 is the initial prediction, F_k is the prediction from k-th tree, and K is number of trees (n_estimators).

For binary classification, the following equations were optimized to find a tree structure that minimized the objective function:

$$L(y_i, p_i) = -(y_i \log p_i + (1 - y_i) \log (1 - p_i))$$

$$\Omega(f) = \gamma T + \frac{1}{2}\lambda \sum_{j=1}^{T} w_j^2 + \alpha \sum_{j=1}^{T} |w_j|$$

$$Obj = \sum_{i=1}^{n} L(y_i, \hat{y}_i) + \sum_{k=1}^{K} \Omega\,(F_k)$$

where, L is a logistic loss function, y_i is true observed label for each i-training sample, p_i is the model predictive value for each i-training sample, T is number of leaves, w_j is leaf weight (represents the output value), α is L1 regularization hyperparameter, λ is L2 regularization hyperparameter and γ is penalty hyperparameter for an additional leaf.

To find the best split, XGBoost calculates the optimal prediction for each leaf as (assuming α = 0 for simplicity):

$$w_j^* = -\frac{G_j}{H_j + \lambda}$$

$$G_j = \sum_{i \in I_j} g_j = \sum_{i \in I_j} \frac{\partial L(y_i, p_i)}{\partial p_i}$$

$$H_j = \sum_{i \in I_j} h_j = \sum_{i \in I_j} \frac{\partial^2 L(y_i, p_i)}{\partial p_i^2}$$

where G_j and H_j is sum of first and second derivative of loss function for the sample in leaf j (I_j).

For a group of samples in a node, the gain from splitting that node to left and right children is:

$$Gain = \frac{1}{2}\left[\frac{G_L^2}{H_L + \lambda} + \frac{G_R^2}{H_R + \lambda} - \frac{(G_L + G_R)^2}{H_L + H_R + \lambda}\right] - \gamma$$

where G_L , H_L are the sums for the left child and G_R , H_R are the sums for the right child. A split only occurs if the gain larger than zero.

Role of the tuned hyperparameters

The hyperparameters optimized via Bayesian search (see "Hyperparameter optimization" in the main text) govern four aspects of XGBoost behavior:

Ensemble size and learning rate. The number of boosting rounds (n_estimators, denoted *K* above) sets how many sequential trees are added to the

ensemble, and the learning rate $\eta \in (0, 1]$ scales each tree's contribution. Smaller η values require more rounds but typically generalize better; we additionally applied early stopping on a held-out portion of the training set (the last two years) to terminate boosting once validation loss stopped improving.

Tree complexity. Maximum tree depth (max_depth) and the minimum sum of instance weight required to form a child node (min_child_weight) jointly limit the depth and granularity of each tree, controlling the model's capacity to fit fine-grained patterns in the training data.

Stochastic regularization. The row-subsampling ratio (subsample) and per-tree column-subsampling ratio (colsample_bytree) introduce randomness into tree construction, reducing variance across the ensemble in a manner analogous to bagging.

Explicit regularization. The penalty hyperparameters γ, α, and λ — corresponding to the minimum loss reduction required to make a split, the L1 penalty on leaf weights, and the L2 penalty on leaf weights respectively — are the regularization terms defined in $\Omega(f)$ above. Together with the stochastic controls, they discourage overly complex trees and improve out-of-sample performance.

Bayesian optimization with the Tree-Structured Parzen Estimator was used to search this joint hyperparameter space, with 5-fold stratified cross-validated log-loss as the objective.
