# Supplementary material for: Machine learning reveals temperature as a key predictor of dengue risk across Thailand’s provinces: A 20-year analysis
Source: PLoS Negl Trop Dis. 2026 Jul 28;20(7):e0014590. doi: 10.1371/journal.pntd.0014590 (PMC13440873; doi:10.1371/journal.pntd.0014590)
Supplement: Supplementary file 2 [file pntd.0014590.s002.pdf]

Supplementary information:

Machine learning reveals temperature as a key predictor of dengue risk across Thailand's provinces: A 20-year analysis

S2 Text. Residual autocorrelation: autoregressive sensitivity analysis

The residual autocorrelation function (Fig. 3) showed significant positive autocorrelation at short lags (1–3 months), indicating that the model leaves some month-to-month temporal dependence unexplained. To determine whether this pattern reflects mis-specification of the estimated environmental relationships, and to confirm that it does not distort our feature-importance findings, we performed a sensitivity analysis that adds a single autoregressive predictor.

We augmented the 54-feature model with one additional predictor, the previous month's observed high-risk status for the same province (prior_risk), constructed by a within-province, chronological one-month shift of the binary target; the first month of each province series is undefined and was handled natively by XGBoost's missing-value mechanism. All other settings — the median-based target definition, the temporal train/validation/test split, and early stopping — were left unchanged. We then recomputed the per-province residual ACF (ordered chronologically and averaged across provinces, as in Fig. 3), the test-period ROC-AUC, and the mean absolute SHAP feature ranking.

Adding the single autoregressive predictor removed the residual autocorrelation: the mean residual ACF fell from 0.54 to 0.14 at lag 1, and no lag exceeded the 95% confidence band (**Figure R1**). Discrimination improved correspondingly (test ROC-AUC from 0.80 to 0.91 pre-COVID; and from 0.74 to 0.89 during/post-COVID). This confirms that the short-lag autocorrelation in the main model arises from a deliberately excluded autoregressive (persistence) component of dengue risk, not from mis-estimated environmental relationships.

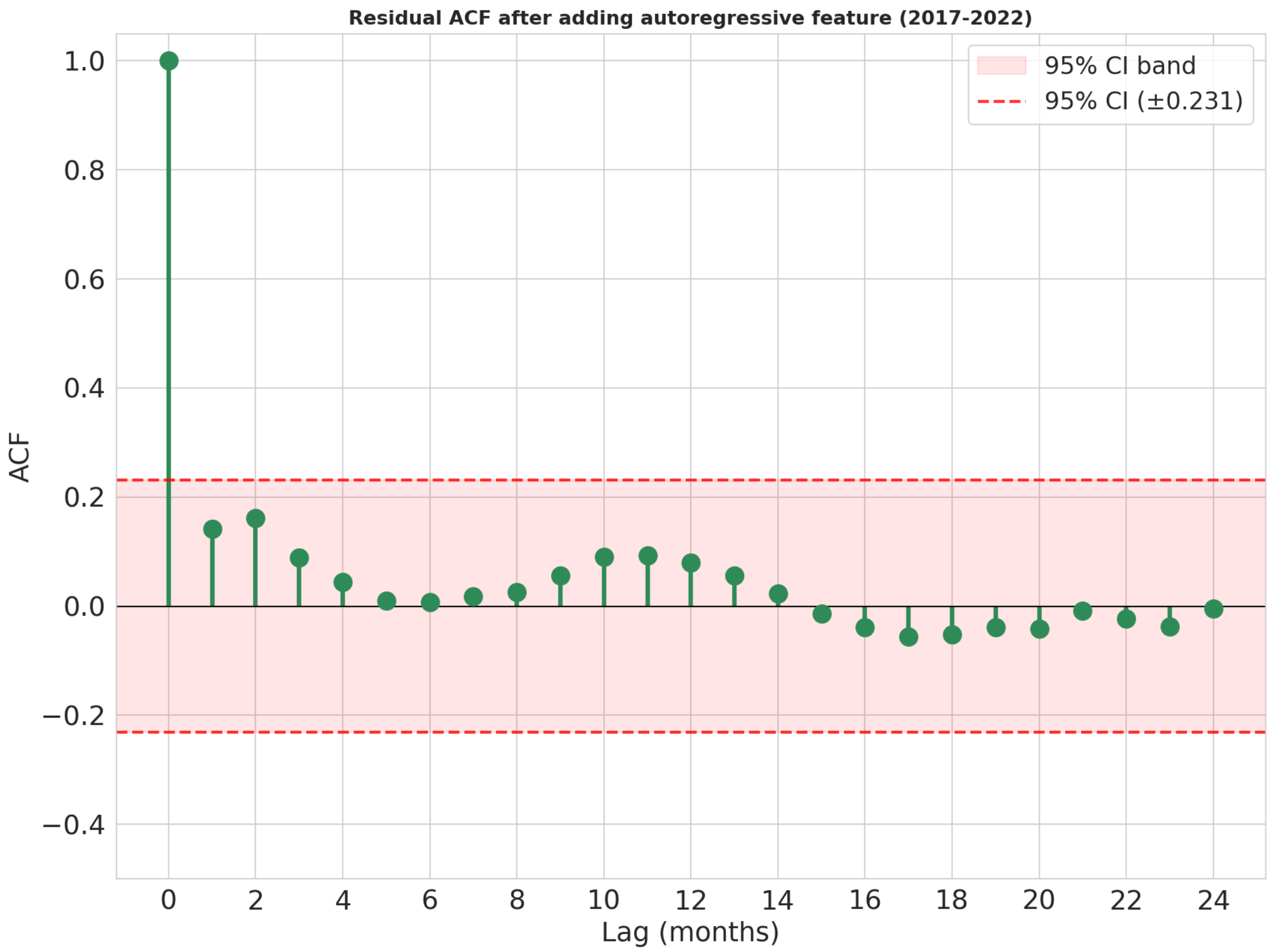

Figure R1. Residual autocorrelation after adding an autoregressive feature. Mean residual ACF across provinces, computed as in Fig. 3, for the model augmented with the previous month's observed high-risk status. The red shaded region is the 95% confidence band $\pm 1.96/\sqrt{T}$, T = 72 months per province). All lags fall within the band, indicating that the short-range residual autocorrelation seen in Fig. 3 is removed.

The added autoregressive term ranked as the most important predictor (**Figure R2**). Comparing the top ten environmental and socioeconomic predictors like-for-like — that is, setting aside the autoregressive term — the number of temperature variables changed from seven to five, with current minimum temperature as the leading predictor (**Table R1**). The dominance of temperature among the environmental and socioeconomic drivers is preserved; we therefore retain the parsimonious environmental-socioeconomic

model in the main analysis, consistent with our objective of identifying transmission drivers rather than producing operational forecasts.

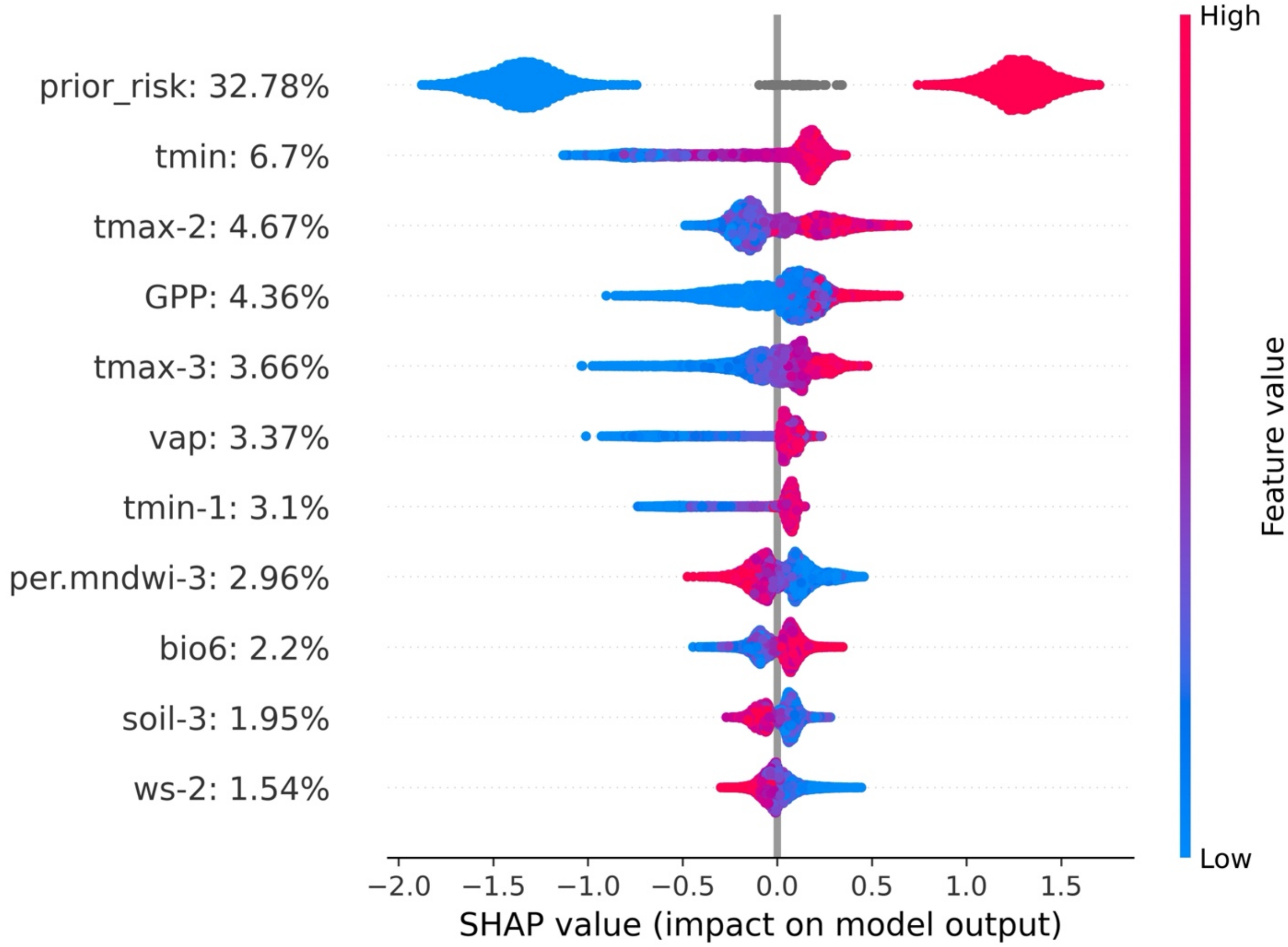

Figure R2. SHAP feature importance for the model including the autoregressive predictor. Beeswarm summary of SHapley Additive exPlanations (SHAP) values for the sensitivity model that augments the 54 environmental and socioeconomic features with a single autoregressive predictor, prior_risk (the previous month's observed high-risk status for the same province), based on 2003–2019 data. The eleven highest-ranked features are shown — prior_risk together with the ten leading environmental and socioeconomic predictors — ordered by mean absolute SHAP value. Percentages indicate each feature's relative contribution among all 55 input features. The autoregressive term is the single most important predictor (32.78% of total importance), yet temperature variables remain the leading environmental drivers.

Table R1. Top-10 environmental and socioeconomic predictors by SHAP importance: main model vs autoregressive sensitivity model. Ranking by mean absolute SHAP value on 2003–2019. The autoregressive predictor (prior_risk) ranks first overall in the +prior_risk model and is omitted here so that the environmental and socioeconomic predictors can be compared like-for-like.

Rank	Main model	+ prior_risk (env./socioecon. only)
1	tmin-1	tmin
2	tmin	tmax-2
3	tmax-3	GPP
4	GPP	tmax-3
5	vap	vap
6	bio6	tmin-1
7	soil-3	per.mndwi-3
8	bio4	bio6
9	bio2	soil-3
10	bio3	ws-2
