# Supplementary material for: Machine learning reveals temperature as a key predictor of dengue risk across Thailand’s provinces: A 20-year analysis
Source: PLoS Negl Trop Dis. 2026 Jul 28;20(7):e0014590. doi: 10.1371/journal.pntd.0014590 (PMC13440873; doi:10.1371/journal.pntd.0014590)
Supplement: Supplementary file 3 [file pntd.0014590.s003.pdf]

Supplementary information:

Machine learning reveals temperature as a key predictor of dengue risk across Thailand's provinces: A 20-year analysis

S3 Text: Sensitivity of results to the risk-classification threshold

Our primary analysis defines the high-risk class using the national median of the training-period DHF incidence, which balances the two classes (50% positive) and limits majority-class bias. To assess whether our conclusions depend on this choice, we repeated the complete modelling pipeline with the high-risk class defined instead by the 75th percentile of the training-period incidence distribution (6.08 cases per 100,000), yielding about 25% positive observations. To accommodate the resulting 3:1 class imbalance we set *scale_pos_weight* = 3 and re-optimized all XGBoost hyperparameters for the re-labelled target using the same Bayesian search, stratified 5-fold cross-validation, and early-stopping procedure described in the main text; the temporal train/test split and the 54-feature set were unchanged.

Out-of-sample discrimination was essentially unchanged relative to the median-based model: the test ROC-AUC was 0.79 for the pre-COVID-19 period (vs. 0.80) and 0.72 for the during- and post-COVID-19 period (vs. 0.74) (**Fig. S7a**). The SHAP feature-importance ranking remained temperature-dominated, with six of the ten leading features temperature-related, 3-month-lagged maximum temperature and 1-month-lagged minimum temperature the two most important predictors, and Gross Provincial Product again ranked fourth (**Fig. S7b**). Precision–recall performance was likewise stable: average precision was 0.68 (vs. 0.70) for the pre-COVID-19 period and 0.27 (vs. 0.26) for the during- and post-COVID-19 period (**Fig. S7c,d**), remaining above the corresponding no-skill baseline in each period. A side-by-side summary is provided in **Table S2**.
