# Supplementary material for: Machine learning reveals temperature as a key predictor of dengue risk across Thailand’s provinces: A 20-year analysis
Source: PLoS Negl Trop Dis. 2026 Jul 28;20(7):e0014590. doi: 10.1371/journal.pntd.0014590 (PMC13440873; doi:10.1371/journal.pntd.0014590)
Supplement: Supplementary file 4 [file pntd.0014590.s011.pdf]

S1 Table: Performance comparison of four machine learning models (logistic regression, support vector machine, random forest, and XGBoost,) using six metrics on training set, pre-COVID-19 (2018–2019) test set, and post-COVID-19 (2022) test set. For each metric, ranking score calculated by summation of assigned rank from best (score = 4) to worst (score = 1) for each metric. The average performance of each model was obtained from average over 10 random seeds. All models were trained on the same training set to ensure fair comparison. Hyperparameters were optimized using 5-fold cross-validation.

**Classification Model**	**Sets**	**Average Performance Metrics**						**Ranking Score**
		**AUC**	**Logloss (lower better)**	**F1**	**Precision**	**Recall**	**Accuracy**	
Logistic regression	Training	0.811	0.526	0.757	0.722	0.795	0.738	12
Support vector machine	Training	0.800	0.534	0.746	0.715	0.780	0.728	6
Random forest	Training	0.930	0.387	0.863	0.813	0.919	0.850	18
XGBoost	Training	**0.966**	**0.345**	**0.906**	**0.880**	**0.932**	**0.900**	**24**
Logistic regression	Pre-COVID-19	0.797	0.589	0.712	0.596	0.882	0.696	15
Support vector machine	Pre-COVID-19	0.792	0.594	0.708	0.596	0.872	0.694	9
Random forest	Pre-COVID-19	**0.812**	**0.583**	0.707	0.578	**0.911**	0.679	15
XGBoost	Pre-COVID-19	0.799	0.584	**0.712**	**0.597**	0.883	**0.696**	**21**
Logistic regression	post-COVID-19	0.688	**0.862**	0.293	0.177	0.851	0.455	14
Support vector machine	post-COVID-19	0.680	0.910	0.290	0.176	0.826	**0.464**	9
Random forest	post-COVID-19	0.711	0.886	0.307	0.184	0.921	0.449	15
XGBoost	post-COVID-19	**0.725**	0.877	**0.314**	**0.189**	**0.931**	0.461	**22**
