# Supplementary material for: Machine learning reveals temperature as a key predictor of dengue risk across Thailand’s provinces: A 20-year analysis
Source: PLoS Negl Trop Dis. 2026 Jul 28;20(7):e0014590. doi: 10.1371/journal.pntd.0014590 (PMC13440873; doi:10.1371/journal.pntd.0014590)
Supplement: Supplementary file 5 [file pntd.0014590.s012.pdf]

Table S2. Comparison of model performance and key feature-importance findings under the median and 75th-percentile risk-classification thresholds.

**Measure**	**Median threshold (~50% positive)**	**75th-percentile threshold (~25% positive)**
Test-set discrimination and precision–recall		
ROC-AUC, pre-COVID-19 (2017–2019)	0.80	0.79
ROC-AUC, during/post-COVID-19 (2020–2022)	0.74	0.72
Average precision, pre-COVID-19 (2017–2019)	0.70	0.68
Average precision, during/post-COVID-19 (2020–2022)	0.26	0.27
Feature importance (SHAP)		
Temperature-related features in top 10	7	6
Two most important features	tmin-1, tmin	tmax-3, tmin-1
Gross Provincial Product (GPP) rank	4	4
