# Supplementary material for: Machine learning reveals temperature as a key predictor of dengue risk across Thailand’s provinces: A 20-year analysis
Source: PLoS Negl Trop Dis. 2026 Jul 28;20(7):e0014590. doi: 10.1371/journal.pntd.0014590 (PMC13440873; doi:10.1371/journal.pntd.0014590)
Supplement: Supplementary file 6 [file pntd.0014590.s013.pdf]

Response to the reviewers’ comments

We would like to submit a revised version of our manuscript entitled “Machine learning reveals temperature as a key predictor of dengue risk across Thailand’s provinces: A 20-year analysis.” We are grateful for the comments and suggestions from the reviewers. We have carefully addressed all comments and made substantial revisions to improve the quality of our manuscript. Please find below our point-by-point response to the comments.

Reviewers' Comments:

Reviewer #1:

Methods:

- Clear Objectives/Hypothesis: Yes.

- Appropriate Study Design: Partially. The workflow is standard, the study has significant "blind spots" regarding clinical scope.

- Population/Sample Size: Appropriate, though limited to specific manifestations.

- Statistical Analysis: Correct.

- Ethical Requirements: Yes.

Comment 1: The manuscript would benefit from a methodological flowchart to help readers visualise the machine learning framework and interpretability steps. For an example of a workflow figure, the authors may refer to Figure 1 in Mobin, M. Multivariate forecasting of dengue infection in Bangladesh: evaluating the influence of data downscaling on machine learning predictive accuracy. BMC Infect Dis 25, 761 (2025). https://doi.org/10.1186/s12879-025-11159-z

Response: We thank the reviewer for this constructive suggestion and for pointing us to a helpful exemplar. We have now added a methodological flowchart (Fig. S1) summarizing the major analytical stages, from raw data acquisition through feature engineering, model selection, hyperparameter tuning, and final evaluation, including the SHAP-based interpretability step. We have also added a new "Methodology workflow" paragraph at the beginning of the Materials and methods section to describe this workflow. The new paragraph reads:

- This study established a framework to identify predictors of dengue risk, illustrated in Fig. S1. The framework applies machine learning to rank the most informative predictors among 54 input features spanning climatic, bioclimatic, socioeconomic, and remote-sensing data. These data were obtained from three main sources: the TerraClimate dataset, the MODIS dataset, and Thai national authorities. Gridded climatic and remote-sensing data were spatially aggregated to the provincial level; lagged variables and bioclimatic indices were then derived from monthly provincial temperature and precipitation series. Socioeconomic data were obtained directly at the provincial level: the biennially reported household income series was linearly interpolated to fill missing years, and all socioeconomic series were then upsampled to monthly resolution by assigning each year's reported value (typically released at year-end) to every month of that year. Once the monthly feature set was constructed, four candidate machine learning models were compared in a preliminary screening step (Table S1). XGBoost was selected based on this comparison; hyperparameters were optimized via Bayesian search with 5-fold cross-validation, and early stopping during boosting was applied using the final two years of the training period as the validation set. The optimized model was then used for performance evaluation and SHAP-based feature-importance analysis.

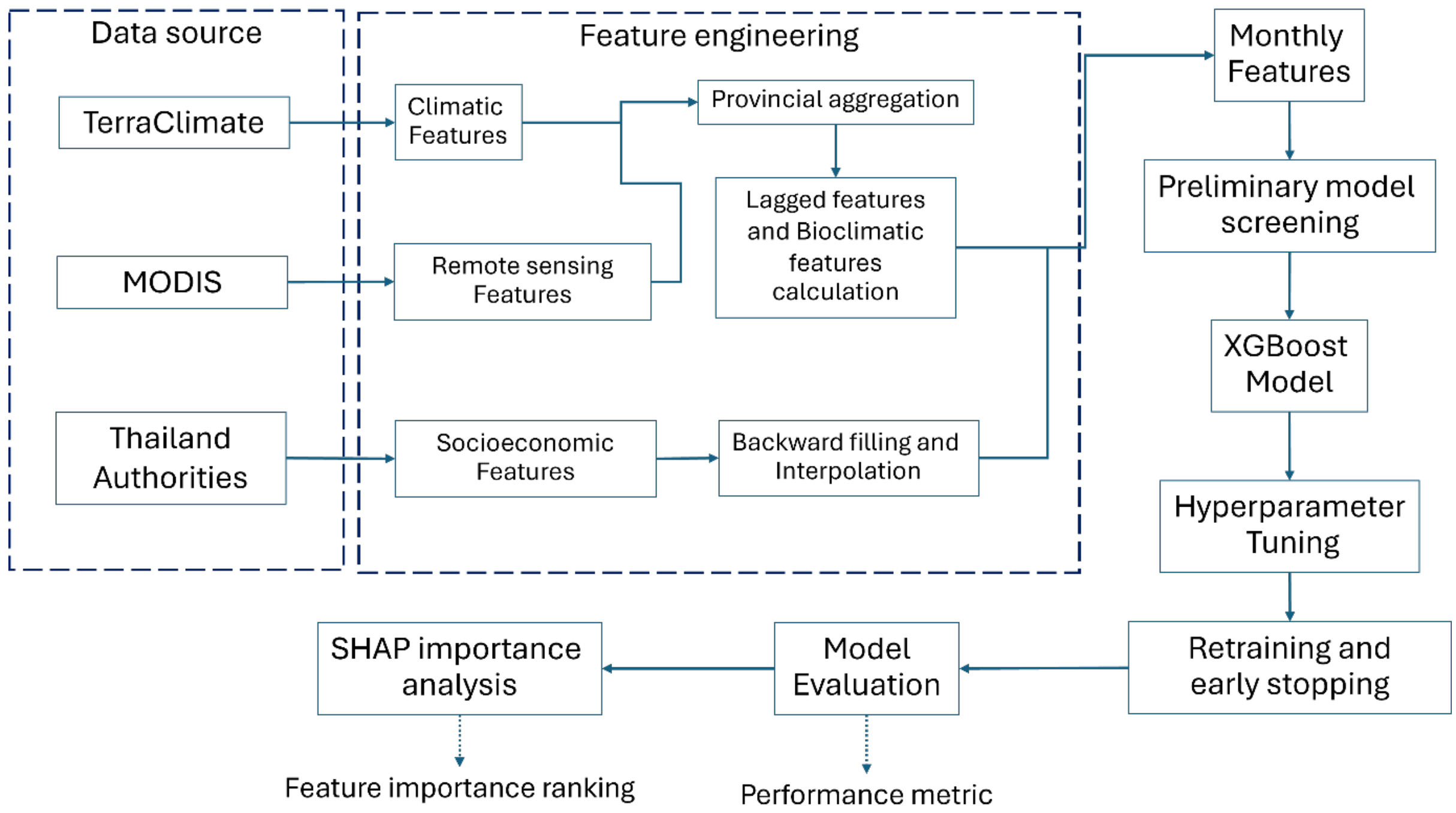

Figure S1. Methodology workflow for the dengue risk classification framework. Solid line shows the flow between stages, and dotted arrow shows results from each analysis.

Results

- Match Analysis Plan: Yes.

- Clear Presentation: Needs improvement regarding data visualisation.

- Figure/Table Quality: Requires revisions for clarity.

Main Manuscript

Comment 2: Table 1: The current format of the table is hard to understand. To improve the overall clarity and reproducibility of the work, the table should be reorganised into a more detailed format with the following columns:

- Index Number

- Variable/Feature Name

- Temporal Granularity

- Data Format

- Description (Unit)

- Source

Response: We thank the reviewer for this concrete suggestion, which has improved both the clarity and the reproducibility of Table 1. We have reorganized the table to include all six requested columns (Index, Variable/Feature, Temporal scale, Data format, Feature description with units, and Source). The continuous indexing across categories makes the total of 54 input features. Lagged variants of the same base feature have been grouped on shared rows for compactness, while retaining unique indices for each lag (e.g., ppt is indexed 1; ppt-1, ppt-2, and ppt-3 are indexed 7–9). The revised Table 1 reads as follows:

Table 1. Variables used in the XGBoost model for dengue risk classification in Thailand.

Index	Variable/ Feature	Temporal scale	Data Format	Feature description (Unit)	Sources
	**Climate**				
1	ppt	Monthly	Grided	Monthly average of precipitation (mm)	TerraClimate (https://www.climatologylab.org/terraclimate.html)
2	tmin	Monthly	Grided	Monthly average of minimum temperature (°C)	
3	tmax	Monthly	Grided	Monthly average of maximum temperature (°C)	
4	vap	Monthly	Grided	Monthly average of vapor pressure (kPa)	
5	soil	Monthly	Grided	Monthly average of soil moisture (mm)	
6	ws	Monthly	Grided	Monthly average of wind speed (m/s)	
7-9	ppt-n; n = 1,2,3	Monthly	Grided	ppt from n month/s prior (mm)	Calculated using above
10-12	tmin-n; n = 1,2,3	Monthly	Grided	tmin from n month/s prior (°C)	
13-15	tmax-n; n = 1,2,3	Monthly	Grided	tmax from n month/s prior (°C)	
16-18	vap-n; n = 1,2,3	Monthly	Grided	vap from n month/s prior (kPa)	
19-21	soil-n; n = 1,2,3	Monthly	Grided	soil from n month/s prior (mm)	
22-24	ws-n; n = 1,2,3	Monthly	Grided	ws from n month/s prior (m/s)	
	**Bioclimatic**			**Temperature-related bio1-bio11**	
25	bio1	Yearly	Per province	Mean annual temperature	

26	bio2	Yearly	Per province	Mean diurnal range (mean of max temp - min temp)	https://rdrr.io/cran/dismo/man/biovars.html [1]
27	bio3	Yearly	Per province	Isothermality (bio2/bio7) (* 100)	
28	bio4	Yearly	Per province	Temperature seasonality (standard deviation *100)	
29	bio5	Yearly	Per province	Max temperature of the warmest month	
30	bio6	Yearly	Per province	Min temperature of the coldest month	
31	bio7	Yearly	Per province	Temperature annual range (bio5-bio6)	
32	bio8	Yearly	Per province	Mean temperature of the wettest quarter	
33	bio9	Yearly	Per province	Mean temperature of driest quarter	
34	bio10	Yearly	Per province	Mean temperature of warmest quarter	
35	bio11	Yearly	Per province	Mean temperature of coldest quarter	
				**Precipitation-related bio12-bio19**	
36	bio12	Yearly	Per province	Total (annual) precipitation	
37	bio13	Yearly	Per province	Precipitation of wettest month	
38	bio14	Yearly	Per province	Precipitation of driest month	
39	bio15	Yearly	Per province	Precipitation seasonality (coefficient of variation)	
40	bio16	Yearly	Per province	Precipitation of wettest quarter	
41	bio17	Yearly	Per province	Precipitation of driest quarter	
42	bio18	Yearly	Per province	Precipitation of warmest quarter	
43	bio19	Yearly	Per province	Precipitation of coldest quarter	
	**Socioeconomic**				
44	GPP	Yearly	Per province	Gross provincial product (million baht)	https://www.nesdc.go.th/nesdb_en/more_news.php?cid=156
45	HouseholdSize	Yearly	Per province	Mean household size (person)	https://stat.bora.dopa.go.th/new_stat/webPage/statByYear.php
46	income	Yearly	Per province	Mean household income (baht)	https://www.nso.go.th/nsoweb/nso/statistics_and_indicators?impt_branch=309
	**Remote sensing**				
47	per.mndwi	Monthly	Grided	Water index: Percentage of area with an MNDWI value	Terra Surface Reflectance 8-Day

				greater than or equal to zero for each month	L3 Global 500 m SIN Grid V005 (MOD09A1)
48	per.ndvi	Monthly	Grided	Vegetation index: Percentage of NDVI pixels that are within 0.5 to 1 for each month	
49-51	per.mndwi-n; n = 1,2,3	Monthly	Grided	Water index from n month/s prior	
52-54	per.ndvi-n; n = 1,2,3	Monthly	Grided	Vegetation index from n month/s prior	
	**Target Class**				
-	Dengue Risk (binary)	Monthly	Per province	Province with higher or equal (1) and lower (0) than median DHF incidence rate (reported cases per 100,000 population)	Bureau of Epidemiology, Thailand

Comment 3: Figure 3: The provinces under the y-axis are unsorted (or sorted based on a specific parameter). To improve the overall readability of the figure, the list under the y-axis should be sorted alphabetically from top to bottom. If the authors have specific reasons for using another sorting criterion, the basis for the sorting should be clearly stated in the figure's legend.

Response: We thank the reviewer for raising this. The provinces in this figure (Fig. 4 in the revised manuscript, owing to renumbering of earlier figures) are arranged by Thailand's administrative regions, and within each region ordered from north to south by latitude. We chose this grouping over alphabetical ordering because dengue transmission in Thailand shows clear regional structure — provinces within the same region share climatic, ecological, and socioeconomic characteristics — and a regional grouping allows the reader to perceive these spatial patterns directly in the heatmap. Alphabetical ordering, by contrast, would interleave provinces from different regions and obscure these patterns. We agree that the original figure did not make this organizing principle clear. In the revised figure, regions are now visually demarcated (with shaded bands and region labels), and the figure caption has been updated to explain the arrangement explicitly. The revised figure now reads:

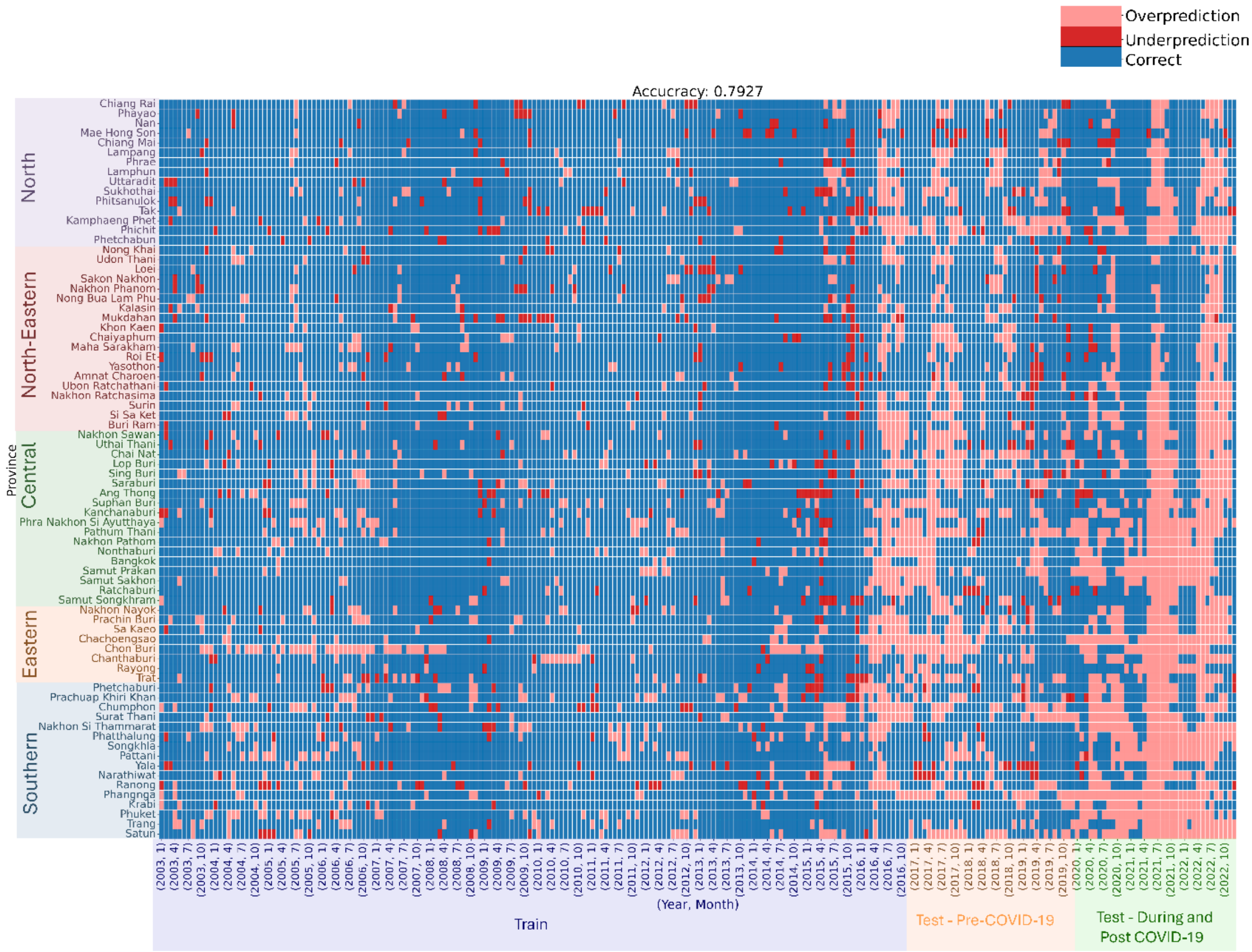

Figure 4. Spatiotemporal evaluation of XGBoost model predictions across Thai provinces, 2003–2022. The heatmap displays monthly prediction accuracy for each province throughout training (2003–2016) and testing (2017–2022) periods. Provinces are grouped by region and ordered from north to south by latitude. Colors indicate prediction outcomes: correct predictions (blue), false positives/overprediction (pink; high risk predicted when actual risk was low), and false negatives/underprediction (red; low risk predicted when actual risk was high); the ground truth is shown in **Fig. S3**. The overall model accuracy is 79.39%.

Comment 4: Figure 5a: The legend/label box currently overlaps with the plot lines. To address this, the label should be positioned in such a way that the AUC curves, as well as the diagonal reference line, are visible.

Response: We thank the reviewer for catching this. We have repositioned the legend in the revised Fig. 6a (formerly Fig. 5a, owing to renumbering of earlier figures) so that it no

longer overlaps with the ROC curves or the diagonal reference line, and all curves and AUC values are now fully visible.

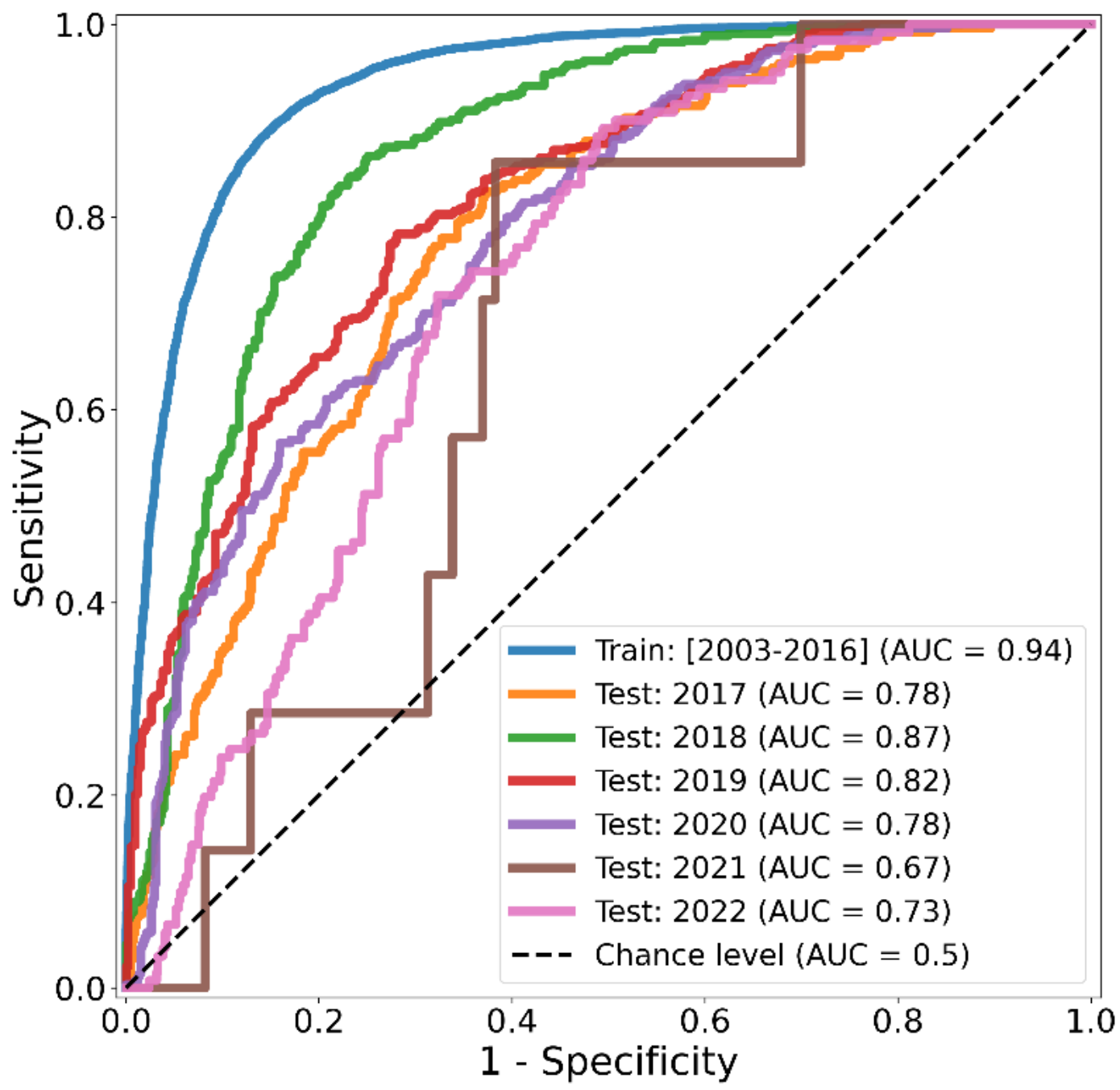

Figure 6a. Receiver Operating Characteristic (ROC) curves showing declining model discrimination ability across periods, with AUC values dropping from pre-pandemic baseline. The diagonal line indicates random classification (AUC = 0.5).

Comment 5: Figure S1 & Figure S5: To improve the overall comparison between the data distribution in Figure S1 and Figure S5, the x-axis (Month) in Figure S1 should be formatted similarly to Figure S5. Additionally, the inclusion of statistical values (e.g., mean, standard deviation, maximum, and minimum) would be beneficial in addition to the raw numerical values/percentages included in the figures.

Response: Revised as suggested. The four statistical values (e.g., mean, standard deviation, maximum, and minimum) calculated from the whole study period were added to each figure's caption.

Comment 6: Figure S3: Similar to Figure 3, the list under the y-axis should be sorted alphabetically from top to bottom to ensure consistency throughout the research paper, including the supplementary materials.

Response: We thank the reviewer for ensuring consistency between the main and supplementary figures. The supplementary heatmap (now Fig. S4 in the revised supplement) presents the ground-truth dengue-risk classification corresponding to the prediction heatmap discussed under Comment 3. We have applied the same organizing principle to both figures: provinces are grouped by Thailand's administrative region and, within each region, ordered from north to south by latitude. This ensures that Figs. 4 and S4 can be read directly against each other on a province-by-province basis.

Comment 7: Figure S4: The current format of the variables' visualisation is cluttered, with the variable/feature name overlapping with the values, which makes interpretation more complicated. To address this, the spacing should be increased or the font size modified to ensure the text is not overlapping. It is highly recommended to group the variables based on categories (e.g., Temperature, Precipitation, Socioeconomic) using the variable name instead of the abbreviation, thereby making the heatmap self-explanatory.

Response: We thank the reviewer for these recommendations, both of which we have engaged with carefully. We note that this figure is now Fig. S5 in the revised supplement.

Regarding the first point: the font size and inter-cell spacing have been adjusted so that variable labels and correlation values no longer overlap, and we agree this materially improves readability. The revised figure is included in the updated supplementary information.

Regarding the second point — grouping variables by category (Temperature, Precipitation, Socioeconomic, etc.) — we have retained the original hierarchical-clustering arrangement, generated using the clustermap function in seaborn, which groups features by their pairwise correlation structure rather than by predefined category. Our reasoning is that the primary purpose of this figure is to expose the multicollinearity structure among the 54 input features and material to interpreting the SHAP feature-

importance rankings. Hierarchical clustering directly visualises which features carry redundant information, regardless of category: for example, several lagged temperature variables cluster tightly with their unlagged counterparts, and certain bioclimatic indices cluster with raw climate features across category boundaries.

Editorial and Data Presentation Modifications

Comment 8: *Introduction*

Structural Logic: To improve the logical flow, consider moving the discussion of virus characteristics (lines 89–95) to follow the first paragraph.

Literature Update

Reference [11] (Verma et al., 2014), reference to neurological manifestations in lines 91–93 appears irrelevant to the discussion on clinical manifestations and should be deleted or replaced with a sentence about the spectrum of Severity.

Reference [30] (Suttirat et al.) is currently a preprint. Please update this with the peer-reviewed version.

Response: Revised as suggested. We moved the virus characteristic paragraph to follow the first paragraph. The reference 11 has been removed and the reference 30 has been updated.

Summary and General Comments

Comment 9: The manuscript is a well-written study. Although the machine learning workflow for classification is standard, the study has significant "blind spots" regarding the clinical scope of the disease and the lack of analysis of different models. These are areas that will be significant for improvement, besides improvements to data visualisation, to ensure that the study reflects the data accurately.

Response: We thank the reviewer for this overall assessment and for identifying the two areas requiring substantive strengthening. We have addressed both in the revised manuscript.

On the clinical-scope "blind spot" — the use of DHF data alone rather than the full DF/DHF/DSS spectrum — we have added an explicit rationale to the Introduction explaining the trade-off between clinical severity and reporting reliability that motivates this choice (responding to Comment 10 below).

On the lack of analysis of different models, we have benchmarked XGBoost against three additional classifiers — logistic regression, support vector machines, and random forest — using six performance metrics on the training set and both testing periods. The results, reported in the new Table S1, show that XGBoost outperforms the other models across all settings and motivates its selection for the main analysis. This addresses both the reviewer's "different models" concern here and Reviewer 2's parallel request (Comment 2 below), and we are grateful that the two reviewers' suggestions converged on a useful strengthening of the manuscript.

Please note the following additional limitations or suggestions for future research in your Discussion section:

Comment 10: The authors exclusively use Dengue Hemorrhagic Fever (DHF) data and do not include the full clinical spectrum of Dengue Fever (DF) and Dengue Shock Syndrome (DSS). At a minimum, the authors must provide a clear rationale in the Introduction explaining why only DHF was selected and why other manifestations were excluded.

Response: We thank the reviewer for raising this point, which has prompted us to make the rationale for our case definition explicit in the Introduction. Our choice to use DHF as the target class reflects a balance between two competing properties of dengue surveillance data: clinical severity and reporting reliability.

DHF is severe enough to bring patients into the formal healthcare system reliably, and it is reported in sufficient numbers across provinces and months to support province-level monthly classification across a 20-year time series. DF, while more common in absolute

terms, is systematically under-reported — particularly by smaller healthcare providers in rural areas — because its milder symptoms often do not trigger hospital presentation or laboratory confirmation. DSS, the most severe manifestation, is reliably reported when it occurs but is too infrequent to support province-level monthly modelling: across the 20-year study period.

DHF therefore represents the operationally tractable point on the severity–reporting trade-off, and the case definition that allows the relationships between environmental, socioeconomic, and disease-risk variables to be identified across the full geographic and temporal scope of our analysis.

The following passage has been added to the Introduction:

- "We focused on DHF cases as a representative measure of dengue risk because they balance clinical severity with reliable reporting. Milder DF cases are systematically under-reported, particularly by smaller healthcare providers, whereas DSS — although the most severe manifestation — occurs at frequencies too low to support province-level monthly classification."

Comment 11: While the classification workflow is standard, the study lacks a comparison between different prediction models. Including such a comparison would strengthen the validity of the chosen framework.

Response: We thank the reviewer for this suggestion, which has materially strengthened the methodological foundation of the study. We have implemented a four-model benchmark comparing XGBoost against three additional classifiers: logistic regression, support vector machines (SVM), and random forest. Each model was trained on the same training period (2003–2016) using the same 54 input features, with hyperparameters optimised by Bayesian search and 5-fold stratified cross-validation. Logistic regression and SVM were trained on Z-score-standardised features, since both are sensitive to feature scaling; XGBoost and random forest do not require standardisation owing to their tree-based architecture.

Performance was assessed across six metrics — AUC, log-loss, F1 score, precision, recall, and accuracy — on the training set, the pre-COVID-19 testing period (2017–2019), and the post-COVID-19 testing period (2022). For each metric, models were ranked from best (4 points) to worst (1 point), and overall rankings were summed across metrics. The results, reported in the new Table S1 of the revised supplementary information, show that XGBoost achieved the highest aggregate ranking score across all three periods (training: 24; pre-COVID-19: 21; post-COVID-19: 22), confirming its selection as the modelling framework for the main analysis.

A brief description of this benchmarking step has been added to the "XGBoost model implementation" subsection of the Methods, reading:

- We employed a supervised machine learning approach to capture intricate feature interactions and classify each province-month as high- or low-risk based on the input features. To identify a suitable algorithm, we first conducted a preliminary comparison in which all 54 features were supplied to four classification models — logistic regression, support vector machine (SVM), random forest, and XGBoost (Table S1). Performance was assessed across six metrics on the training set and both testing periods; XGBoost outperformed the other algorithms across all settings and was therefore selected for downstream analysis.

Comment 12: The inclusion of analysis code and data is available; the authors would enhance the study's impact and accessibility by hosting these on a public repository such as GitHub. This would facilitate better version control for future updates and streamline the ability for other researchers to reproduce these findings.

Response: We thank the reviewer for this advice. We have deposited the analysis code and processed data to Zenodo, which mints a persistent DOI tied to the published version of the manuscript. We chose Zenodo as the primary citation target because it offers two properties valuable for scientific reproducibility:

Persistent identification. Zenodo issues a DOI backed by long-term archival commitments from CERN, with retention guaranteed for the lifetime of the host laboratory. It is the citation format preferred by most major scientific journals for data and code. GitHub URLs, by contrast, can change or become inaccessible if the repository is renamed, transferred, or made private.

Frozen version-of-record. The Zenodo deposit captures a snapshot of the code and data exactly as used to generate the published results, providing an unambiguous reference for anyone seeking to reproduce our findings.

The materials supporting this manuscript will be publicly accessible at https://doi.org/10.5281/zenodo.17192398 upon acceptance. During peer review, the same materials are available at https://bit.ly/DengueTH_20years to allow the editor and reviewers to inspect the code and data directly.

Reviewer #2:

Methods

Comment 1: Time-Series Diagnostics: Since this is a 20-year monthly dataset, the authors must report the Autocorrelation Function (ACF) of the residuals. Without this, it is unclear if the model has adequately accounted for temporal dependencies or if the residuals contain unresolved patterns.

Response: We thank the reviewer for this methodologically important comment, which has led to a substantive addition to the manuscript. We agree that for a 20-year monthly dataset, the residual ACF is the appropriate diagnostic to check whether the model has captured the temporal structure of the data.

To address this, we computed the ACF of the model residuals (defined as predicted minus observed probability of high-risk classification) on the testing period (2017–2022), separately for each of Thailand's 77 provinces. The per-province ACFs were then averaged across provinces at each lag to summarize the country-level residual temporal structure. The 95% confidence band was constructed under the null hypothesis of no autocorrelation, scaled by the per-province testing-set length. The result is shown as a new Fig. 3 in the revised manuscript: the mean residual ACF remains within the 95% band across all lags considered, indicating no systematic residual autocorrelation across provinces. We interpret this as evidence that the XGBoost model adequately captured the temporal dependencies in the data without leaving systematic structure in the errors.

We have added a paragraph to the "Model performance and validation" subsection of the Results describing this analysis, and the figure caption explains the construction of the ACF and the confidence band:

- We further examined whether the model captured the temporal structure of the data by computing the autocorrelation function (ACF) of the residuals on the predicted probabilities. Residuals were calculated separately for each province, and the per-province ACFs were averaged across provinces to summarize the country-level temporal structure (Fig. 3). The mean ACF in the testing period lay

within the 95% confidence band, indicating no systematic residual autocorrelation across provinces and supporting the conclusion that the model adequately captured temporal dependencies in the data.

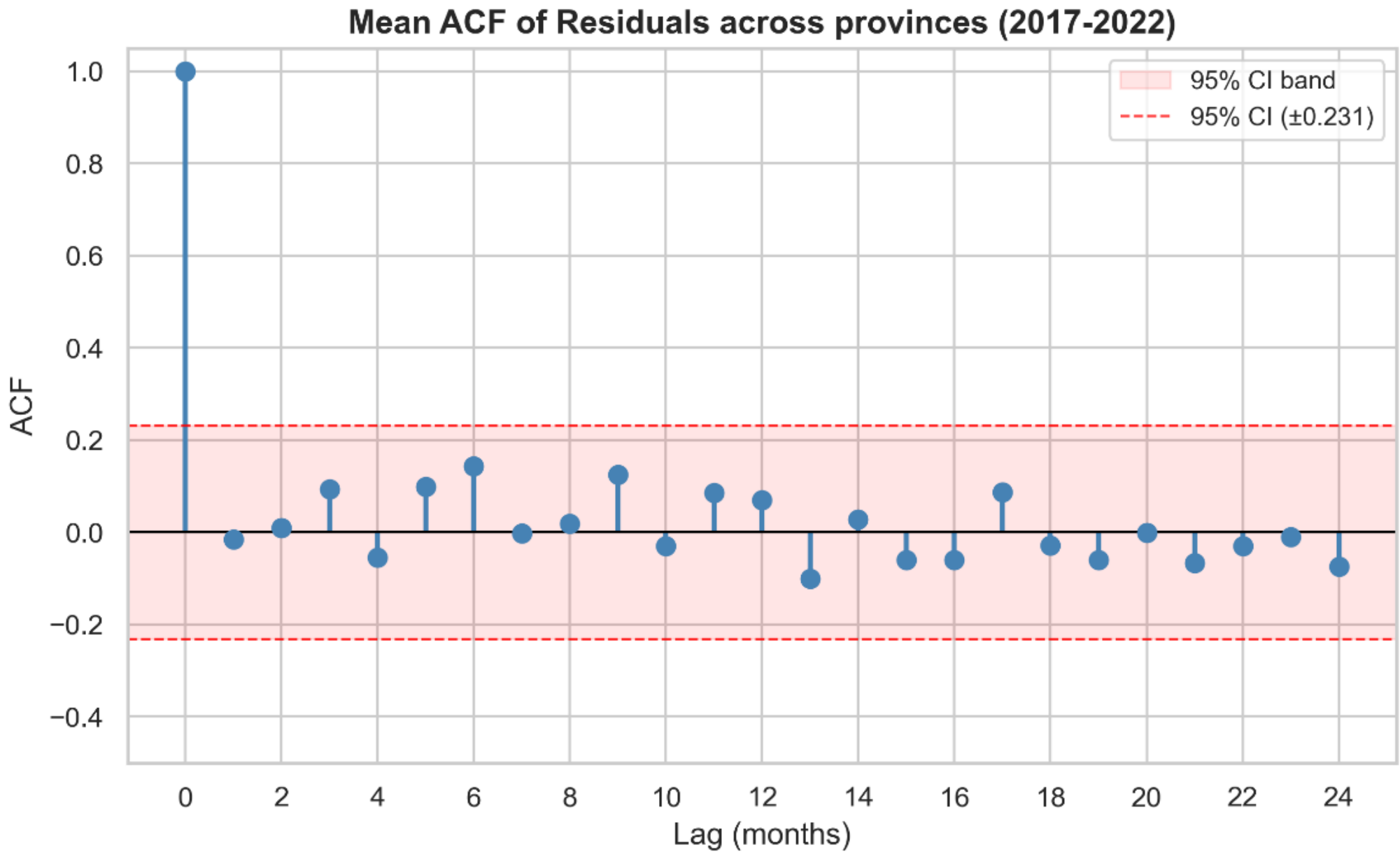

Figure 3. Mean autocorrelation function (ACF) of model residuals across Thai provinces, testing period (2017–2022). Residuals were computed as the difference between predicted and observed probabilities of high-risk classification for each province-month. The ACF was calculated separately for each province at each lag, and the per-lag values were then averaged across all 77 provinces to summarize the country-level temporal structure. Red bands indicate 95% confidence intervals under the null hypothesis of no autocorrelation, derived from the per-province testing-set length. Mean ACF values lying within the bands indicate the absence of systematic residual autocorrelation, supporting the conclusion that the model adequately captured the temporal dependencies in the data.

Comment 2: Benchmarking: The study lacks a baseline comparison (e.g., logistic regression or a GAM). Without a simpler benchmark, the performance gain of the XGBoost model cannot be objectively evaluated.

Response: We thank the reviewer for this important methodological recommendation. We agree that a baseline comparison is essential for objectively evaluating the performance gain attributable to XGBoost specifically, rather than to machine learning approaches in general.

We have benchmarked XGBoost against three alternative classifiers spanning a range of model complexities: logistic regression (a linear baseline), support vector machine (SVM), and random forest (a tree-based ensemble baseline that shares architectural elements with XGBoost). Each model was trained on the same training period (2003–2016) using the same 54 input features. Logistic regression and SVM were trained on Z-score-standardised features, since both are sensitive to feature scaling; XGBoost and random forest do not require standardization owing to their tree-based architecture. Hyperparameters for all four models were optimized using the same Bayesian search procedure with 5-fold stratified cross-validation on the training set, ensuring a fair comparison.

Performance was assessed across six metrics — AUC, log-loss, F1 score, precision, recall, and accuracy — evaluated on the training set, the pre-COVID-19 testing period (2017–2019), and the post-COVID-19 testing period (2022). For each metric within each period, models were ranked from best (4 points) to worst (1 point), and the aggregate ranking score was summed across the six metrics. The results, reported in the new Table S1, are summarised below:

Table S1: Performance comparison of four machine learning models (logistic regression, support vector machine, random forest, and XGBoost,) using six metrics on training set, pre-COVID-19 (2018–2019) test set, and post-COVID-19 (2022) test set. For each metric, ranking score calculated by summation of assigned rank from best (score = 4) to worst (score = 1) for each metric. The average performance of each model was obtained from average over 10 random seeds. All models were trained on the same training set (2007–2015) to ensure fair comparison. Hyperparameters were optimized using 5-fold cross-validation.

**Classification Model**	**Sets**	**Average Performance Metrics**						**Ranking Score**
		**AUC**	**Logloss (lower better)**	**F1**	**Precision**	**Recall**	**Accuracy**	
Logistic regression	Training	0.811	0.526	0.757	0.722	0.795	0.738	12
Support vector machine	Training	0.800	0.534	0.746	0.715	0.780	0.728	6
Random forest	Training	0.930	0.387	0.863	0.813	0.919	0.850	18
XGBoost	Training	**0.966**	**0.345**	**0.906**	**0.880**	**0.932**	**0.900**	**24**
Logistic regression	Pre-COVID-19	0.797	0.589	0.712	0.596	0.882	0.696	15
Support vector machine	Pre-COVID-19	0.792	0.594	0.708	0.596	0.872	0.694	9

Random forest	Pre-COVID-19	**0.812**	**0.583**	0.707	0.578	**0.911**	0.679	15
XGBoost	Pre-COVID-19	0.799	0.584	**0.712**	**0.597**	0.883	**0.696**	**21**
Logistic regression	post-COVID-19	0.688	**0.862**	0.293	0.177	0.851	0.455	14
Support vector machine	post-COVID-19	0.680	0.910	0.290	0.176	0.826	**0.464**	9
Random forest	post-COVID-19	0.711	0.886	0.307	0.184	0.921	0.449	15
XGBoost	post-COVID-19	**0.725**	0.877	**0.314**	**0.189**	**0.931**	0.461	**22**

XGBoost achieved the highest aggregate ranking score across all three periods (training: 24; pre-COVID-19: 21; post-COVID-19: 22), confirming its selection as the modelling framework. To point out this change, these sentences have been added in the "XGBoost model implementation" section

- "We employed a supervised machine learning approach to capture intricate feature interactions and classify each province-month as high- or low-risk based on the input features. To identify a suitable algorithm, we first conducted a preliminary comparison in which all 54 features were supplied to four classification models — logistic regression, support vector machine (SVM), random forest, and XGBoost (Table S1). Performance was assessed across six metrics on the training set and both testing periods; XGBoost outperformed the

other algorithms across all settings and was therefore selected for downstream analysis. XGBoost constructs an ensemble of decision trees through gradient boosting, combined with an approximate tree learning technique that scales efficiently to large feature sets [2]. The XGBoost algorithms work based on additive training, where a new tree is constructed to correct the errors made by the previous trees. The new tree was iteratively created to minimize the errors until the errors could not be reduced further, or a specific stop condition was met."

Comment 3: Calibration: The evaluation relies on AUC/F1 scores (discrimination) but lacks probability calibration (e.g., calibration plots or Brier scores). This is essential for public health tools where the accuracy of the risk probability is as important as the final classification.

Response: We thank the reviewers for the comment. We agree that the probability calibration is a great additon to this work. We calculated the Brier scores of each dataset as follows: Training: 0.116, Pre-COVID-19 Testing: 0.198 and During and post-COVID-19: 0.282 (Random guess: 0.250). This show that the model predicted probability is still adequate in non-COVID-19 testing period while the during and post COVID-19 period model probability shows poor predictive performance. This is expected because the disease outbreak pattern changes in this period. The reported cases were lower than expected**.** To reflect this point these following senstenses have been added to the **Model performance and validation** subsection:

- "Furthermore, we calculated brier score of each dataset as follows: training: 0.116, pre-COVID-19 testing: 0.198 and during and post-COVID-19: 0.282. This shows that the model predicted probability is still acceptable in non-COVID-19 testing period while the during and post COVID-19 period model probability shows poor predictive performance."

Results

Comment 4: The results are well-presented, particularly the SHAP plots, which offer excellent transparency into the model's decision-making process. The identification of Tmin as a primary driver is significant.

Response: We thank the reviewer for this positive assessment. We agree that SHAP-based interpretability is one of the methodological strengths of this work, and we are pleased that the identification of minimum temperature as a primary driver — and the biological plausibility of the associated ~21°C threshold — came through clearly. We have further strengthened the interpretive treatment of this finding in the revised Discussion by connecting the statistical thresholds we recover to the experimental literature on *Aedes aegypti* thermal biology, as part of our response to Comment 6 below.

Comment 5: However, the "Results" section would be strengthened by including a calibration analysis (Brier score or calibration plot) to demonstrate the reliability of the predicted risk probabilities. Furthermore, the performance dip during the 2021 COVID-19 period is a critical result that requires more nuanced reporting to distinguish between model failure and genuine epidemiological decoupling.

Response: We thank the reviewer for these two related recommendations. We have addressed both in the revised manuscript.

On the calibration analysis: we have added a Brier score analysis to the Results section, computed separately for each evaluation period (training, pre-COVID-19, and during- and post-COVID-19). The full reporting and interpretation are provided in our response to Comment 3 above. The following text has been added to the Discussion:

- Brier scores corroborate the AUC pattern: 0.116 on training, 0.198 in the pre-COVID-19 testing period, and 0.282 during and after the pandemic. The pre-pandemic value indicates well-calibrated probabilities, consistent with the AUC of 0.80 in the same period. The pandemic-era Brier, while only modestly higher in absolute terms, reflects a substantive failure of calibration: the empirical positive rate collapsed (only 7 high-risk province-months in 2021, Fig. S4), yet the model's

probability outputs remained anchored to the pre-pandemic base rate, producing the systematic overprediction. The model's predicted probabilities should accordingly not be used for public-health decision-making during periods of major epidemiological disruption without first recalibrating to the prevailing base rate.

On the COVID-19 performance dip: we agree that the original framing did not adequately distinguish *model failure* (in the sense of the modelling framework being inadequate for the underlying epidemiology) from *genuine epidemiological decoupling* (in the sense that dengue dynamics during 2020–2022 were driven by mechanisms outside the model's input space). The reviewer's framing is the right one — the model did not fail in the sense of misrepresenting the relationships it was given; rather, the relationships it learned from pre-pandemic data no longer adequately characterised the system once non-environmental drivers (mobility changes, altered healthcare-seeking behavior, surveillance disruption) became dominant. We have rewritten the relevant passages of the Results and Discussion to make this distinction explicit. The revised Discussion now reads, in part:

- The decline in model performance during the pandemic period — with AUC falling to 0.62 in 2021 — points to the important role of behavioral and surveillance factors in shaping observed dengue dynamics, none of which were represented among our environmental and socioeconomic predictors. Human mobility in Bangkok changed substantially during this period in response to government interventions and shifts in everyday behavior [3]. For a vector-borne pathogen, a central driver of secondary transmission (captured by the basic reproductive number R_0) is the rate of vector–human contact, principally mosquito biting frequency, which depends on overlap between human activity and vector habitat [4]. Pandemic restrictions plausibly disrupted this overlap by shifting human activity patterns indoors and reducing the spatial range of host movement, thereby lowering effective biting exposure even where vector populations were unchanged.

- Beyond mobility, healthcare-seeking behavior was also altered during the pandemic. Concerns about nosocomial COVID-19 transmission, hospital access restrictions, and the diversion of clinical and laboratory capacity reduced the probability that febrile dengue patients were assessed, confirmed, and reported [5-10]. Observed reductions in DHF therefore reflect both declines in transmission and reductions in case ascertainment. Both mechanisms, mobility-driven changes in exposure and surveillance disruption, operate on dimensions of transmission orthogonal to our climatic and socioeconomic predictors, and each would be expected to produce the systematic overprediction observed during 2020–2022.

Conclusions

Comment 6: The conclusions are generally supported by the data, but the link between the statistical findings and biological mechanisms is currently thin. The authors should more explicitly connect the Tmin thresholds to the biological limits of the Aedes mosquito or the viral extrinsic incubation period (EIP). Additionally, the conclusion regarding the model's use for public health officials should be tempered until probability calibration is performed.

Response: We thank the reviewer for this important comment, which has prompted two substantive improvements to the Discussion. To clarify this point, the following text has been added to the "Temperature as the primary predictor of dengue risk" subsection:

- The threshold effect we identified at approximately 21°C for minimum temperature corresponds closely with established biological constraints on *Aedes aegypti*–mediated dengue transmission. Several parameters governing mosquito behavior — including biting rate, adult lifespan, and developmental rate — drop sharply below approximately 21°C compared with their optima in the 25–30°C range [11, 12]. Below this threshold, viral replication within *Ae. aegypti* is also substantially reduced [11, 12]. Complementary experimental work on *Ae. albopictus* (a secondary dengue vector) places the lower dissemination limit at

approximately 18°C, at which DENV-2 establishes midgut infection but does not reach the salivary glands [13].

- Direct experimental evidence for a thermal threshold on dengue transmission comes from Watts et al. [14], who fed *Ae. aegypti* on DEN-2-viremic rhesus monkeys and then incubated the engorged mosquitoes at constant temperatures ranging from 20 to 35°C before allowing them to transmit to susceptible recipients. At 20°C, virus established in the mosquito midgut but failed to reach the salivary glands within a 25-day observation window. At 24°C and 26°C, salivary-gland infection eventually developed but transmission to recipient monkeys still did not occur, even when the infecting viral dose was increased — implying that salivary-gland viral titres at these temperatures remained below the threshold required to infect a host via the bite. Transmission occurred only at 30°C and above, and the extrinsic incubation period (EIP) shortened steeply with temperature, from 25 days at 30°C with a low infecting dose, to 12 days at 30°C with a higher dose, and to 7 days at 32°C and 35°C. Together these findings indicate that low ambient temperatures act as a biological barrier to dengue transmission through two related mechanisms — failure of viral dissemination from the midgut at the coolest temperatures, and insufficient salivary-gland viral titres at intermediate temperatures — and that warmer conditions accelerate the EIP, expanding the temporal window in which infected mosquitoes can transmit before dying.

On the operational tempering: we agree that the original Conclusions overstated the model's readiness for public-health deployment given the calibration limitations now made explicit through the Brier score analysis (see our response to Comment 3 above). The revised Conclusions now states that

- "The COVID-19 period exposed important limitations of environmentally driven prediction. Discrimination dropped to AUC = 0.62 in 2021 and the model systematically overpredicted risk. We accordingly recommend that the model's predicted probabilities not be used for public-health decision-making during

periods of major epidemiological disruption without first recalibrating the model. More broadly, our findings suggest that the next generation of dengue prediction systems will need to combine environmental signals with real-time indicators of mobility, vector populations, and surveillance coverage to remain reliable through periods of societal change."

We have also added a parallel tempering statement in the Discussion:

- Brier scores corroborate the AUC pattern: 0.116 on training, 0.198 in the pre-COVID-19 testing period, and 0.282 during and after the pandemic. The pre-pandemic value indicates well-calibrated probabilities, consistent with the AUC of 0.80 in the same period. The pandemic-era Brier, while only modestly higher in absolute terms, reflects a substantive failure of calibration: the empirical positive rate collapsed (only 7 high-risk province-months in 2021, Fig. S4), yet the model's probability outputs remained anchored to the pre-pandemic base rate, producing the systematic overprediction. The model's predicted probabilities should accordingly not be used for public-health decision-making during periods of major epidemiological disruption without first recalibrating to the prevailing base rate.

Editorial and Data Presentation Modifications

Comment 7: Please provide a more detailed step-by-step clarification on the interpolation methods used to synchronize biennial socioeconomic data with monthly climatic records.

Response: We thank the reviewer for prompting a careful re-examination of this step, which uncovered an error in the original data-processing pipeline that we have now corrected. We are grateful for the opportunity to address this before publication.

The original error. In the original analysis, the biennially reported household-income series was inadvertently interpolated *after* the annual-to-monthly upsampling step rather than *before* it. This produced an inconsistency in which the income values for the years

with reported data were constant across all twelve months (as intended), while the values for the interpolated missing years increased linearly month-by-month (as an artefact of the misordered pipeline). The intended behaviour was for the interpolation to fill in the missing years at annual resolution first, after which all annual values would be propagated to monthly resolution by simple repetition.

The corrected pipeline. We have rerun the analysis with the correct processing order:

1. The biennially reported household-income series was linearly interpolated using the pandas *interpolate* method at annual resolution, filling missing years by fitting a straight line between adjacent observed years.
2. All socioeconomic series were then upsampled from annual to monthly resolution by assigning each year's reported value to every month of that year (since these data are reported by the Thai authorities at year-end, the December value is taken to represent conditions across the full year).

Impact on results. We rechecked all figures and tables against the corrected pipeline. The overall scientific conclusions of the manuscript are unchanged: temperature remains the dominant predictor (seven of the top ten features), the ~21°C minimum-temperature threshold and ~32°C maximum-temperature threshold persist, precipitation features remain minor contributors, and higher GPP remains associated with elevated risk. The model's AUC values in each evaluation period are unchanged at the level of reporting precision (pre-pandemic 0.80; during- and post-pandemic 0.74).

Two minor changes appear in the SHAP-based feature importance ranking:

- The 2nd- and 3rd-ranked features swap positions, though their absolute SHAP contributions differ only marginally.
- tmax-2 (previously 9th-ranked) is replaced at this position by mean diurnal range (bio2).

Figure R1 below (provided to reviewers but not included in the published manuscript) shows side-by-side comparisons of the SHAP rankings and performance metrics before and after the correction, confirming the small magnitude of these effects.

Original results

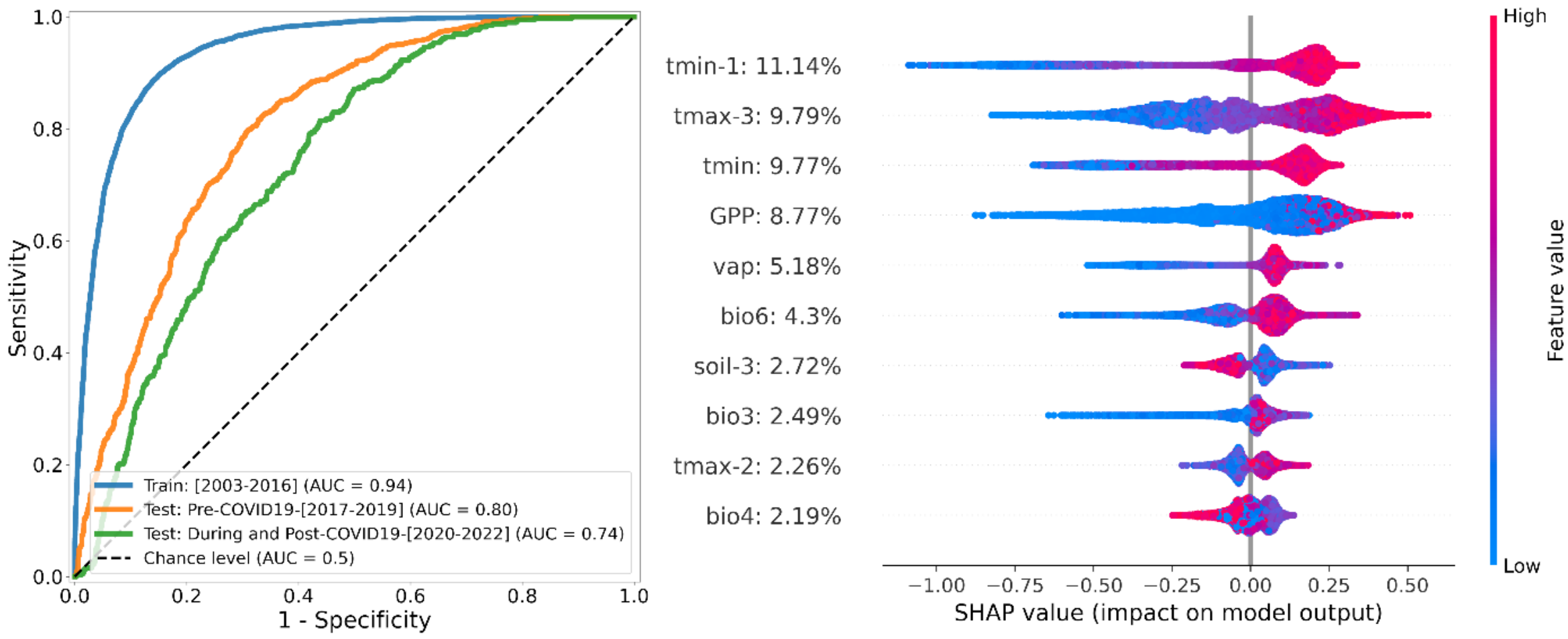

Corrected results

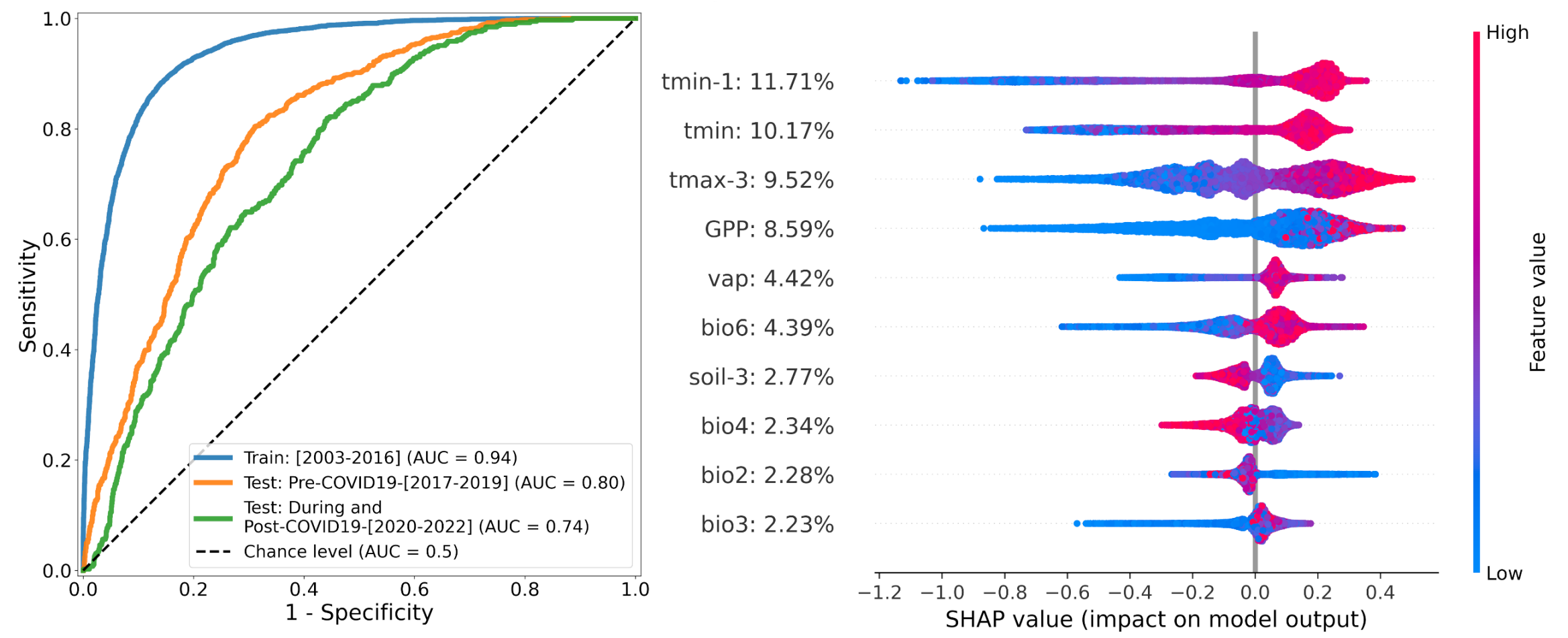

Figure R1. Comparison between model performance and SHAP importance analysis between original and new results.

Manuscript revisions. We have added the following clarification to the "Socioeconomic indicators" subsection of the Methods:

- “The household income series, reported biennially by the Thai authorities, was linearly interpolated using the pandas *interpolate* method to fill missing years. All socioeconomic series were then upsampled from annual to monthly resolution by assigning each year's reported value to every

month of that year, on the assumption that annual values represent conditions across the full year.”

Summary and General Comments

Comment 6: This manuscript provides a valuable 20-year analysis of DHF risk in Thailand using an interpretable machine learning approach. The use of SHAP to move beyond "black-box" predictions is highly commendable and aligns with the needs of public health stakeholders.

Response: We thank the reviewer for this positive summary assessment. We share the view that interpretable approaches such as SHAP are essential for translating machine learning outputs into actionable information for public-health stakeholders, and we have aimed throughout the revision to deepen the substantive value of the interpretability analysis including by connecting the statistical thresholds it recovers to the experimental thermal-biology literature on *Aedes aegypti*.

Comment 7: However, the manuscript currently lacks several standard statistical safeguards necessary for time-series modeling. The most critical omission is the lack of residual autocorrelation (ACF) testing and a baseline model comparison. Addressing these points, along with providing a calibration analysis, will significantly strengthen the paper’s findings and ensure the model is reliable for real-world application. I have recommended a major revision to allow the authors to incorporate these essential diagnostic steps.

Response: We thank the reviewer for clearly identifying the three diagnostic gaps that the original manuscript needed to address, and we have implemented all three in the revised submission.

Residual autocorrelation testing (response to Comment 1). We computed the autocorrelation function of the model residuals separately for each of Thailand's 77 provinces over the testing period (2017–2022), then averaged the per-province ACFs at each lag. The mean residual ACF remains within the 95% confidence band across all lags,

indicating that the model captured the temporal structure of the data without leaving systematic residual autocorrelation. The result is presented as a new Fig. 3.

Baseline model comparison (response to Comment 2). We benchmarked XGBoost against three alternative classifiers spanning the model-complexity axis — logistic regression (linear), support vector machine (kernel-based non-linear), and random forest (tree-ensemble) — using six performance metrics evaluated on the training set and both testing periods. XGBoost achieved the highest aggregate ranking across all three periods. The full comparison is reported in the new Table S1.

Calibration analysis (response to Comment 3). We computed Brier scores for each evaluation period (training: 0.116; pre-COVID-19 testing: 0.198; during- and post-COVID-19 testing: 0.282) and added an interpretive paragraph to the Discussion. We have also tempered the operational claims in the Conclusions accordingly.

Reviewer #3

Summary and General Comments

Comment 1: The paper is clearly written, and presents interesting and somehow counterintuitive results. However the methodology section needs to be improved, in order to properly let the readers understanding how to replicate and assess the limitations of the proposed approach.

Response: We thank the reviewer for this overall assessment and for emphasising that the methodology section must enable independent replication and informed evaluation of the approach's limitations. We have made substantive additions to the Methods to address this, building on the reviewer's specific recommendations in Comments 2 through 7 below. In summary:

- A new **Methodology workflow** paragraph and accompanying flowchart (Fig. S1) now provide a high-level overview of the analytical pipeline before the detailed subsections that follow.
- A new **S1 Text** in the supplementary information presents the full mathematical description of XGBoost, including the loss function, regularization terms, optimal leaf-weight calculation, and split-gain criterion.
- A new **Role of the tuned hyperparameters** section in S1 Text explains the role of each tuned hyperparameter.
- The Methods now includes an explicit **rationale for skipping feature normalization and detrending** for XGBoost.
- The **training-vs-testing AUC framing** has been revised throughout to present the training AUC as a fit diagnostic rather than as a performance result.
- The **analysis code and processed data** have been deposited to Zenodo for independent replication.

Detailed responses to each specific recommendation follow below.

Comment 2: The methodology section lacks some information. A paragraph with a suitable figure that explains the entire software pipeline, with a complete description and schematic representation of each input (predictors)/output sample. A schematic representation of the machine learning model is also needed.

Response: We thank the reviewer for this recommendation, which closely parallels the request raised by Reviewer 1 in their Comment 1. We have addressed both concerns through the same set of additions:

First, we have added a new **Methodology workflow** paragraph at the beginning of the Materials and methods section, describing the analytical pipeline from raw data acquisition through feature engineering, preliminary model screening, hyperparameter optimisation with early stopping, performance evaluation, and SHAP-based feature-importance analysis.

Second, we have added **Fig. S1**, a flowchart summarising the workflow visually. The figure represents the data sources (TerraClimate, MODIS, and Thai national authorities), the predictor categories that feed into the model (climatic, bioclimatic, socioeconomic, and remote-sensing features, totalling 54 inputs), the binary classification target (province-month high/low risk based on the national median DHF incidence), and the analytical outputs at each stage.

Third, regarding the schematic representation of the XGBoost model specifically, we have added a new **S1 Text** to the supplementary information providing the full mathematical specification of the algorithm, including the additive tree-building procedure, the regularized objective function for binary classification, the closed-form leaf-weight optimum, and the split-gain criterion. A separate subsection in S1 Text explains the role of each tuned hyperparameter.

Together, the workflow paragraph, Fig. S1, and S1 Text provide both the high-level pipeline overview and the algorithm-level mathematical detail that the reviewer recommended.

Comment 3: A concise mathematical description of the model is currently missing. It would be useful to properly understand the role and meaning of the hyperparameters, and how they are optimized.

Response: We thank the reviewers for the comment. We have now included the mathematical description of XGBoost model in the supplementary material. To clarify the role and meaning of the hyperparameters, the following text has been added to the supplementary information:

Role of the tuned hyperparameters

The hyperparameters optimized via Bayesian search (see "Hyperparameter optimization" in the main text) govern three aspects of XGBoost behavior:

Ensemble size and learning rate. The number of boosting rounds (n_estimators, denoted K above) sets how many sequential trees are added to the ensemble, and the learning rate $\eta \in (0, 1]$ scales each tree's contribution. Smaller η values require more rounds but typically generalize better; we additionally applied early stopping on a held-out portion of the training set (the last two years) to terminate boosting once validation loss stopped improving.

Tree complexity. Maximum tree depth (max_depth) and the minimum sum of instance weight required to form a child node (min_child_weight) jointly limit the depth and granularity of each tree, controlling the model's capacity to fit fine-grained patterns in the training data.

Stochastic regularization. The row-subsampling ratio (subsample) and per-tree column-subsampling ratio (colsample_bytree) introduce randomness into tree construction, reducing variance across the ensemble in a manner analogous to bagging.

Explicit regularization. The penalty hyperparameters γ, α, and λ — corresponding to the minimum loss reduction required to make a split, the L1 penalty on leaf weights, and the L2 penalty on leaf weights respectively — are the regularization terms defined in $\Omega(f)$

above. Together with the stochastic controls, they discourage overly complex trees and improve out-of-sample performance.

Bayesian optimization with the Tree-Structured Parzen Estimator was used to search this joint hyperparameter space, with 5-fold stratified cross-validated log-loss as the objective.

Comment 4: A key aspect that should be explained in the implementation details is data normalization, that may improve training and testing performances. If no normalization is performed, the authors should explain why). Additionally, they should explain in detail if they filtered out means and trends from the data to capture the seasonal or time invariant dependencies.

Response: We thank the reviewer for raising both points, which we agree warranted explicit treatment in the Methods. We have added a dedicated paragraph to the "XGBoost model implementation" subsection covering both questions, summarized here and quoted in full below.

On feature normalization. Normalization and standardization are essential for linear and distance-based classifiers (such as logistic regression and the support vector machine) because both rely on feature magnitudes — through coefficient-weighted sums and distance computations respectively. Decision-tree ensembles such as XGBoost, by contrast, are invariant to any monotonic transformation of an individual feature, since each split is determined by the *relative ordering* of feature values rather than their magnitudes. Standardizing or rescaling the inputs to XGBoost therefore has no effect on the model's behaviour. To ensure a fair comparison in the baseline benchmark (Comment 2 above), the logistic regression and SVM models were trained on Z-score-standardized features while XGBoost and random forest were trained on the raw features.

On detrending and deseasonalizing. We did not filter out means, trends, or seasonal components from the input features prior to training XGBoost. The strict stationarity assumed by classical time-series models such as ARIMA and SARIMA does not apply to tree-based classifiers: XGBoost can learn lagged and seasonal relationships directly from the raw feature series, provided these patterns are present in the training data and

stable enough to generalize. Two pieces of evidence support this approach in our setting. First, the autocorrelation function of the model residuals on the testing period remains within the 95% confidence band across all lags (new Fig. 3, in response to Reviewer 2 Comment 1), indicating that the model captured the temporal structure of the data without leaving systematic residual autocorrelation. Second, the study's analytical goal is to *identify* the environmental and socioeconomic predictors of dengue risk via SHAP-based feature importance, rather than to *forecast* monthly outbreak probabilities for specific province-months — the latter being the application for which strict stationarity is more critical.

The new paragraph in the manuscript reads:

- Unlike linear or distance-based classifiers, decision-tree ensembles such as XGBoost are invariant to monotonic transformations of individual features, since splits depend on the relative ordering of feature values rather than their magnitudes. We therefore did not standardize or rescale inputs prior to training XGBoost. For comparability, the logistic regression and support vector machine baselines were trained on Z-score-transformed features, as both are sensitive to feature scale. We also did not detrend or deseasonalize the input features: XGBoost does not require the strict stationarity assumed by traditional time-series models such as ARIMA or SARIMA, and the autocorrelation analysis of model residuals (Fig. 3) confirmed that the trained model captured the temporal structure of the data without leaving systematic residual autocorrelation. We note further that the goal of this study is to identify environmental and socioeconomic predictors of dengue risk through feature-importance analysis, rather than to produce point forecasts of monthly outbreak probabilities.

Comment 5: Training performances cannot be compared directly with testing. The authors should contrast the AUC = 0.74 with the AUC = 0.8, both derived by testing data.

Response: We thank the reviewer for catching this. We agree that directly comparing training AUC with testing AUC is misleading, since the training value is a fit diagnostic rather than an out-of-sample performance measure. We have revised the framing

throughout the manuscript so that performance comparisons are made consistently between the two held-out testing periods — pre-COVID-19 (2017–2019, AUC = 0.80) and during- and post-COVID-19 (2020–2022, AUC = 0.74) — rather than between training and testing.

Comment 6: The overprediction may derive from the lack of normalization, and by the choice of training the model only with old data. Additionally, attributing overproduction to (increased) mobility changes is a statement that should be discussed in more detail.

Response: We thank the reviewer for the comment. Normalization is not impacting the model prediction because of tree-based model nature of XGBoost (see comment 4 for more detail). Based on our previous work [3], we found that the mobility in Bangkok during COVID-19 period changes drastically due to government intervention and changing human behaviors (Figure R2). This period of low mobility coincides with the low numbers of reported DHF cases from the national disease surveillance.

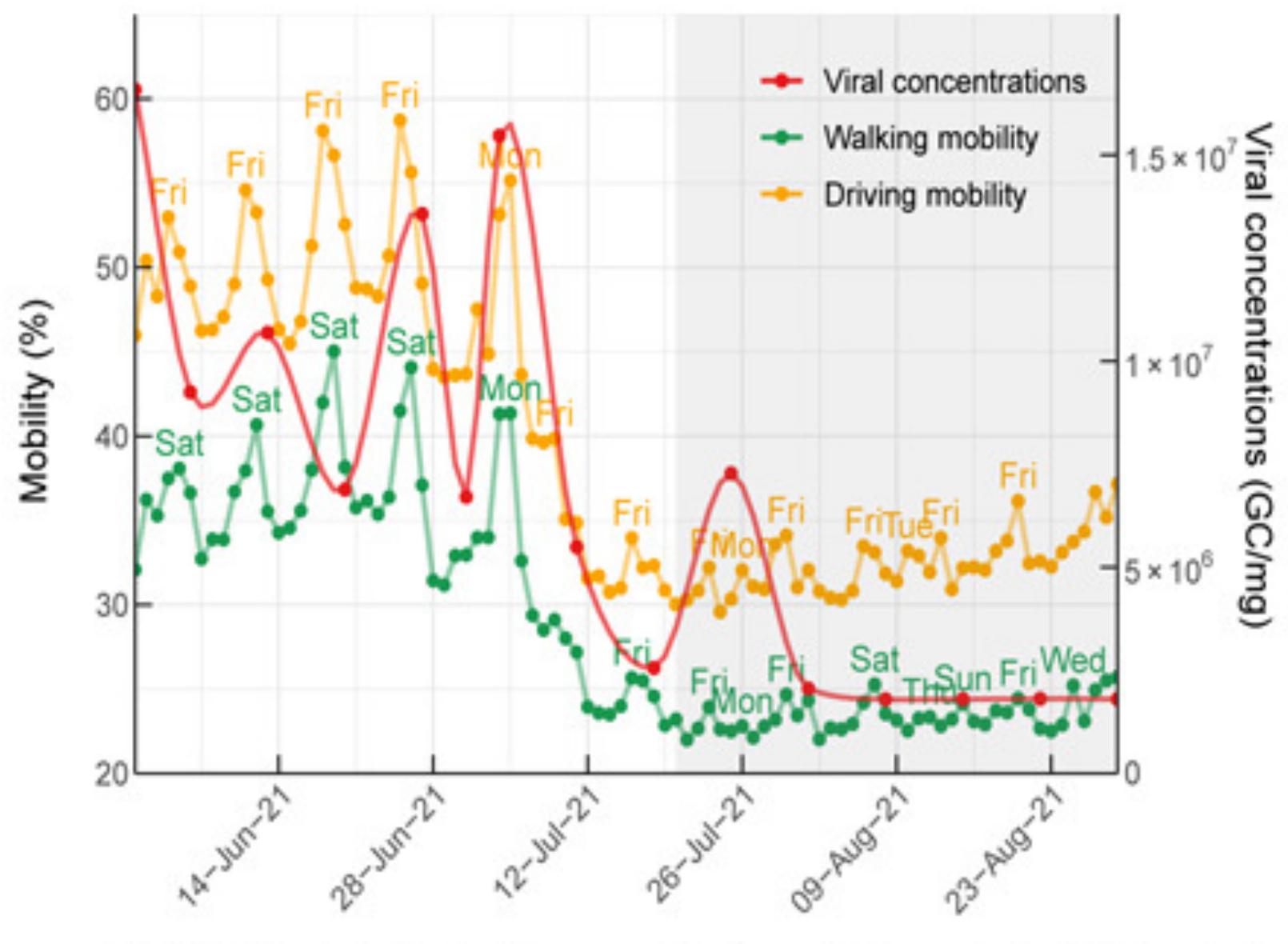

Figure R2. The SARS-CoV-2 viral concentration and changes in mobility (%) as indicated by the ratio of trips from homes to other locations classified by transportation type (driving and walking) compared to before the pandemic (January 13, 2020), which was defined as 100.

To address this comment, the subsection of the “impact of the COVID-19 pandemic” in Discussion has been revised, which now read

- “The decline in model performance during the pandemic period — with AUC falling to 0.62 in 2021 — points to the important role of behavioral and surveillance factors in shaping observed dengue dynamics, none of which were represented among our environmental and socioeconomic predictors. Human mobility in Bangkok changed substantially during this period in response to government interventions and shifts in everyday behavior [3]. For a vector-borne pathogen, a central driver of secondary transmission (captured by the basic reproductive number R_0) is the rate of vector–human contact, principally mosquito biting frequency, which depends on overlap between human activity and vector habitat [4]. Pandemic restrictions plausibly disrupted this overlap by shifting human activity patterns indoors and reducing the spatial range of host movement, thereby lowering effective biting exposure even where vector populations were unchanged.
- Beyond mobility, healthcare-seeking behavior was also altered during the pandemic. Concerns about nosocomial COVID-19 transmission, hospital access restrictions, and the diversion of clinical and laboratory capacity reduced the probability that febrile dengue patients were assessed, confirmed, and reported [5-10]. Observed reductions in DHF therefore reflect both declines in transmission and reductions in case ascertainment. Both mechanisms, mobility-driven changes in exposure and surveillance disruption, operate on dimensions of transmission orthogonal to our climatic and socioeconomic predictors, and each would be expected to produce the systematic overprediction observed during 2020–2022.
- The persistence of reduced model performance extending into 2022 suggests potential long-term alterations in dengue epidemiology. These changes might encompass shifts in population immunity patterns and modifications in vector population dynamics. More broadly, purely environmental models, however well-calibrated under stationary conditions, can fail substantially during periods of

major societal disruption [5, 6]. Future modeling efforts should consider incorporating real-time mobility data, healthcare utilization patterns, and other behavioral indicators to maintain predictive accuracy during unprecedented circumstances."

Comment 7: FIGURE 3. Again, the lack of separation in the figure between training and testing data is misleading. Training set performances are only diagnostics of the training process, and cannot be listed as prediction results.

Response: We thank the reviewer for this consistent methodological position, which reinforces the framing change implemented in response to Comment 5 above. We agree that training-set performance is a fit diagnostic rather than a prediction result, and that figures presenting both should clearly distinguish the two regimes.

We have revised the affected figures accordingly. In the revised Fig. 4 (the spatiotemporal prediction heatmap, formerly Fig. 3) and the revised Fig. S4 (the corresponding ground-truth heatmap, formerly Fig. S3), the x-axis now includes a shaded band demarcating the training period (2003–2016) from the testing period (2017–2022).

We thank the reviewers once again for their valuable feedback and constructive comments, which have significantly improved the quality of our manuscript. We believe that the revised manuscript now addresses all the concerns raised, and hope that it is now suitable for publication in PLOS Neglected Tropical Diseases.

Sincerely,

Charin Modchang

On behalf of all co-authors

References

1. Hijmans RJ, Phillips S, Leathwick J, Elith J, Hijmans MRJ. Package ‘dismo’. Circles. 2017;9(1):1–68.
2. Chen T, Guestrin C. XGBoost: A Scalable Tree Boosting System. Proceedings of the 22nd ACM SIGKDD International Conference on Knowledge Discovery and Data Mining; San Francisco, California, USA: Association for Computing Machinery; 2016. p. 785–94.
3. Anupong S, Chadsuthi S, Hongsing P, Hurst C, Phattharapornjaroen P, Rad S.M AH, et al. Exploring indoor and outdoor dust as a potential tool for detection and monitoring of COVID-19 transmission. iScience. 2024;27(3). doi: 10.1016/j.isci.2024.109043.
4. Delamater PL, Street EJ, Leslie TF, Yang YT, Jacobsen KH. Complexity of the Basic Reproduction Number (R(0)). Emerg Infect Dis. 2019;25(1):1–4. doi: 10.3201/eid2501.171901. PubMed PMID: 30560777; PubMed Central PMCID: PMCPMC6302597.
5. Chen Y, Li N, Lourenço J, Wang L, Cazelles B, Dong L, et al. Measuring the effects of COVID-19-related disruption on dengue transmission in southeast Asia and Latin America: a statistical modelling study. The Lancet infectious diseases. 2022;22(5):657–67.
6. Wu Q, Dong S, Li X, Yi B, Hu H, Guo Z, et al. Effects of COVID-19 Non-Pharmacological Interventions on Dengue Infection: A Systematic Review and Meta-Analysis. Frontiers in Cellular and Infection Microbiology. 2022;Volume 12 - 2022. doi: 10.3389/fcimb.2022.892508.
7. Anupong S, Chadsuthi S, Hongsing P, Hurst C, Phattharapornjaroen P, SM AHR, et al. Exploring indoor and outdoor dust as a potential tool for detection and monitoring of COVID-19 transmission. iScience. 2024;27(3). doi: 10.1016/j.isci.2024.109043.
8. Changruenngam S, Bicout DJ, Modchang C. How the individual human mobility spatio-temporally shapes the disease transmission dynamics. Scientific Reports. 2020;10(1):11325. doi: 10.1038/s41598-020-68230-9.
9. Cummings DA, Irizarry RA, Huang NE, Endy TP, Nisalak A, Ungchusak K, et al. Travelling waves in the occurrence of dengue haemorrhagic fever in Thailand. Nature. 2004;427(6972):344–7.
10. Ramadona AL, Tozan Y, Wallin J, Lazuardi L, Utarini A, Rocklöv J. Predicting the dengue cluster outbreak dynamics in Yogyakarta, Indonesia: a modelling study. The Lancet Regional Health-Southeast Asia. 2023;15.
11. Mordecai EA, Cohen JM, Evans MV, Gudapati P, Johnson LR, Lippi CA, et al. Detecting the impact of temperature on transmission of Zika, dengue, and chikungunya using mechanistic models. PLoS neglected tropical diseases. 2017;11(4):e0005568.
12. Mordecai EA, Caldwell JM, Grossman MK, Lippi CA, Johnson LR, Neira M, et al. Thermal biology of mosquito-borne disease. Ecology letters. 2019;22(10):1690–708.
13. Liu Z, Zhang Z, Lai Z, Zhou T, Jia Z, Gu J, et al. Temperature increase enhances Aedes albopictus competence to transmit dengue virus. Frontiers in microbiology. 2017;8:2337.

14. Watts DM, Burke DS, Harrison BA, Whitmire RE, Nisalak A. Effect of temperature on the vector efficiency of Aedes aegypti for dengue 2 virus. 1986.
