# Supplementary material for: Machine learning reveals temperature as a key predictor of dengue risk across Thailand’s provinces: A 20-year analysis
Source: PLoS Negl Trop Dis. 2026 Jul 28;20(7):e0014590. doi: 10.1371/journal.pntd.0014590 (PMC13440873; doi:10.1371/journal.pntd.0014590)
Supplement: Supplementary file 7 [file pntd.0014590.s014.pdf]

Reviewer Comments on Revised Manuscript

Manuscript ID: PNTD-D-25-01878_R1

Title: Machine learning reveals temperature as a key predictor of dengue risk across Thailand's provinces: A 20-year

Dear Editor,

I have reviewed the revised manuscript and the authors' responses to the comments from the previous review round.

The authors have addressed the major concerns raised in my initial review and have substantially improved the manuscript. The revisions strengthen both the methodological rigor and the interpretation of the findings.

In particular, the inclusion of residual autocorrelation diagnostics provides additional evidence that the model adequately captures temporal patterns in dengue transmission. The expanded benchmarking analyses also offer a clearer justification for the selection of the XGBoost framework relative to alternative approaches. Furthermore, the addition of probability calibration metrics improves the evaluation of model performance and complements the discrimination measures already presented.

The revised discussion is notably stronger than in the original submission. The authors have provided a more convincing interpretation of the temperature-related findings by linking the identified thresholds to established biological mechanisms associated with mosquito survival, viral development, and dengue transmission. The discussion of the COVID-19 period has also been expanded and now acknowledges potential effects of mobility restrictions, healthcare-seeking behavior, and surveillance-related disruptions that may have influenced disease dynamics and model performance.

One issue raised in my previous review remains only partially addressed. I suggested a sensitivity analysis using an alternative threshold for risk classification, such as the 75th percentile of incidence, to evaluate the robustness of the binary classification framework. Although such an analysis would provide additional support for the stability of the results, I consider it a secondary issue and do not regard it as essential for the validity of the main conclusions.

Overall, the revised manuscript is scientifically sound and represents a meaningful improvement over the original submission. The authors have satisfactorily addressed the principal concerns raised during review, and the remaining issue is relatively minor.

Recommendation: Accept with Minor Revision.

Sincerely,

Reviewer
