# Supplementary material for: Machine learning reveals temperature as a key predictor of dengue risk across Thailand’s provinces: A 20-year analysis
Source: PLoS Negl Trop Dis. 2026 Jul 28;20(7):e0014590. doi: 10.1371/journal.pntd.0014590 (PMC13440873; doi:10.1371/journal.pntd.0014590)
Supplement: Supplementary file 8 [file pntd.0014590.s015.pdf]

Response to Reviewers

Dear Editor and Reviewers,

We thank the Academic Editor and the reviewers for their positive assessment of our revised manuscript and for the additional comments provided. We are grateful that Reviewer #3 considers the work suitable for publication and that Reviewer #2 recommends acceptance. Although the remaining comments were offered as optional minor revisions, we have addressed each of them in full to further strengthen the manuscript. Below, we reproduce each comment in italics, followed by our point-by-point response. Newly added or revised manuscript text is shown in quotation marks, and all corresponding changes are marked in the tracked-changes version of the manuscript.

Academic Editor

Comment: *Thank you for the thoughtful revision of your manuscript. I consider this acceptable, but two of the reviewers have provided additional comments that you can consider for very minor revisions if you wish.*

Response: We thank the Academic Editor for the positive assessment of our revised manuscript. We have addressed the two additional comments raised by Reviewer #1 and the suggestion raised by Reviewer #2 in full, as detailed below.

Reviewers' Comments:

Reviewer #1:

Comment 1: (Table 1 formatting). *I appreciate the authors' effort to reorganise Table 1 into a more structured format; it is much more readable now. However, the "Data Format" column is conceptually incorrect and contains a recurring typo. (1) Conceptual mismatch: the terms "Grided" and "Per province" do not describe data formats; they describe the spatial scale or spatial resolution of the data. (2) Typographical error: "Gridded" is consistently misspelt as "Grided" throughout this column. Recommendation: please keep the "Data Format" column but remove the spatial terms and instead populate it with the true technical data formats used (e.g., Raster, NetCDF, CSV, Shapefile, Float, or Integer).*

Response: We thank the reviewer for this careful observation and for the positive comment on the table's readability. We agree that the previous entries ("Grided" / "Per province") describe the spatial scale of the data rather than its format, and that "Gridded" had been misspelt. We have revised the "Data Format" column of Table 1 so that it now reports the technical data type of each variable as used in the model: all continuous predictors (the climate variables and their lags, the bioclimatic variables, the socioeconomic indicators, and the remote-sensing indices and their lags) are floating-point values ("Float"), and the binary classification target is an integer ("Integer"). The spatial descriptors and the spelling error have been removed throughout the column. The revised Table 1 now reads:

Table 1. Variables used in the XGBoost model for dengue risk classification in Thailand.

**Index**	**Variable/ Feature**	**Temporal scale**	**Data Format**	**Feature description (Unit)**	**Sources**
	**Climate**				
1	ppt	Monthly	Float	Monthly average of precipitation (mm)	TerraClimate (https://www.climatologylab.org/terraclimate.html)
2	tmin	Monthly	Float	Monthly average of minimum temperature (°C)	
3	tmax	Monthly	Float	Monthly average of maximum temperature (°C)	
4	vap	Monthly	Float	Monthly average of vapor pressure (kPa)	

5	soil	Monthly	Float	Monthly average of soil moisture (mm)	
6	ws	Monthly	Float	Monthly average of wind speed (m/s)	
7-9	ppt-n; n = 1,2,3	Monthly	Float	ppt from n month/s prior (mm)	Calculated using above
10-12	tmin-n; n = 1,2,3	Monthly	Float	tmin from n month/s prior (°C)	
13-15	tmax-n; n = 1,2,3	Monthly	Float	tmax from n month/s prior (°C)	
16-18	vap-n; n = 1,2,3	Monthly	Float	vap from n month/s prior (kPa)	
19-21	soil-n; n = 1,2,3	Monthly	Float	soil from n month/s prior (mm)	
22-24	ws-n; n = 1,2,3	Monthly	Float	ws from n month/s prior (m/s)	
	**Bioclimatic**			**Temperature-related bio1-bio11**	
25	bio1	Yearly	Float	Mean annual temperature	https://rdrr.io/cran/dismo/man/biovars.html [42]
26	bio2	Yearly	Float	Mean diurnal range (mean of max temp - min temp)	
27	bio3	Yearly	Float	Isothermality (bio2/bio7) (* 100)	
28	bio4	Yearly	Float	Temperature seasonality (standard deviation *100)	
29	bio5	Yearly	Float	Max temperature of the warmest month	
30	bio6	Yearly	Float	Min temperature of the coldest month	
31	bio7	Yearly	Float	Temperature annual range (bio5-bio6)	
32	bio8	Yearly	Float	Mean temperature of the wettest quarter	
33	bio9	Yearly	Float	Mean temperature of driest quarter	
34	bio10	Yearly	Float	Mean temperature of warmest quarter	
35	bio11	Yearly	Float	Mean temperature of coldest quarter	
				**Precipitation-related bio12-bio19**	
36	bio12	Yearly	Float	Total (annual) precipitation	
37	bio13	Yearly	Float	Precipitation of wettest month	
38	bio14	Yearly	Float	Precipitation of driest month	
39	bio15	Yearly	Float	Precipitation seasonality (coefficient of variation)	
40	bio16	Yearly	Float	Precipitation of wettest quarter	
41	bio17	Yearly	Float	Precipitation of driest quarter	
42	bio18	Yearly	Float	Precipitation of warmest quarter	

43	bio19	Yearly	Float	Precipitation of coldest quarter	
	**Socioeconomic**				
44	GPP	Yearly	Float	Gross provincial product (million baht)	https://www.nesdc.go.th/nesdb_en/more_news.php?cid=156
45	HouseholdSize	Yearly	Float	Mean household size (person)	https://stat.bora.dopa.go.th/new_stat/webPage/statByYear.php
46	income	Yearly	Float	Mean household income (baht)	https://www.nso.go.th/nsoweb/nso/statistics_and_indicators?impt_branch=309
	**Remote sensing**				
47	per.mndwi	Monthly	Float	Water index: Percentage of area with an MNDWI value greater than or equal to zero for each month	Terra Surface Reflectance 8-Day L3 Global 500 m SIN Grid V005 (MOD09A1)
48	per.ndvi	Monthly	Float	Vegetation index: Percentage of NDVI pixels that are within 0.5 to 1 for each month	
49-51	per.mndwi-n; n = 1,2,3	Monthly	Float	Water index from n month/s prior	
52-54	per.ndvi-n; n = 1,2,3	Monthly	Float	Vegetation index from n month/s prior	
	**Target Class**				
-	Dengue Risk (binary)	Monthly	Integer	Province with higher or equal (1) and lower (0) than median DHF incidence rate (reported cases per 100,000 population)	Bureau of Epidemiology, Thailand

Comment 2: (Introduction — case definition: DHF vs. DF/DSS). *Thank you for the explanation regarding the operational trade-offs of using DHF data. The current revision does not fully satisfy the recommendation. The newly added rationale in the Introduction still requires a minor adjustment: the authors state that DF is "systematically under-reported" and that DSS is "too infrequent to support province-level monthly modelling," but these claims are currently unsupported. The authors must provide citations to show that DF is under-reported in this specific context and that DSS data are too sparse. The manuscript will be fully resolved once appropriate supporting citations are added to the Introduction.*

Response: We thank the reviewer for this point and have added supporting evidence for both claims in the Introduction.

Under-reporting of milder dengue fever (DF). We now support this statement with three references. Wichmann et al. (2011), using prospective cohort data benchmarked against the Thai national surveillance system (the same system that provides our case data) found that total symptomatic dengue is under-recognized approximately 8.7-fold. Undurraga et al. (2013) estimated a regional reporting rate of approximately 13% and noted that under-reporting is greatest for the mildest cases. Thisyakorn et al. (2022) similarly concluded that the dengue burden in Thailand is underreported, owing to reliance on passive surveillance and the self-management of mild cases. The revised sentence reads:

- "We focused on DHF cases as a representative measure of dengue risk because they balance clinical severity with reliable reporting. Milder DF cases are systematically under-reported [37-39], ..."

Infrequency of DSS. We have quantified this directly from our own dataset, using the same incidence-based rule that defines our risk target. Across the 18,240 province-months in the study period, the median DSS incidence is exactly zero: 59.0% of province-months recorded no DSS cases, 78.0% recorded at most one, and only 5.9% recorded five or more. The median training-period DHF incidence used to threshold our target is 2.78 per 100,000 and produces a balanced (50/50) high-/low-risk split. If we apply the

identical rule to DSS, whose median incidence is zero, this would label every province-month as high-risk and yield no usable target. We have added this justification to the Introduction. The revised sentences read:

- “We focused on DHF cases as a representative measure of dengue risk because they balance clinical severity with reliable reporting. Milder DF cases are systematically under-reported [37-39], whereas dengue shock syndrome (DSS), although the most severe manifestation, is too infrequent to support province-level monthly classification. Across the 18,240 province-months of the 2003–2022 study period, 59.0% recorded no DSS cases, and the median DSS incidence is zero; applying the same median-incidence rule used to define our binary target therefore labels every province-month as high-risk for DSS, in contrast to DHF, whose median incidence (2.78 per 100,000) yields the balanced high- and low-risk split used here (see Risk classification section for more details).”

We note that Reviewer #1 indicated “No further comment” on the Methods, Results, Conclusions, and Summary sections, and we are grateful for the thorough and constructive evaluation across both rounds of review.

Reviewer #2:

Comment 1: *One issue raised in my previous review remains only partially addressed. I suggested a sensitivity analysis using an alternative threshold for risk classification, such as the 75th percentile of incidence, to evaluate the robustness of the binary classification framework. Although such an analysis would provide additional support for the stability of the results, I consider it a secondary issue and do not regard it as essential for the validity of the main conclusions. Recommendation: Accept with Minor Revision.*

Response: We thank the reviewer for this suggestion and for recommending acceptance. To assess whether our findings depend on the median risk threshold, we repeated the complete analysis using the 75th percentile of the training-period DHF incidence (6.08 cases per 100,000) as the high-/low-risk cut-point in place of the median. We re-optimized all XGBoost hyperparameters for the re-labelled target and set *scale_pos_weight* = 3 to account for the resulting 3:1 class imbalance; the temporal split, cross-validation scheme, and feature set were otherwise unchanged.

Out-of-sample discrimination was essentially identical to the median-based model, with test ROC-AUCs of 0.79 (pre-COVID-19) and 0.72 (during/post-COVID-19), compared with 0.80 and 0.74. Temperature-related variables continued to dominate the SHAP ranking, occupying six of the ten most important features, with 3-month-lagged maximum temperature and 1-month-lagged minimum temperature the two leading predictors and Gross Provincial Product again ranked fourth. Precision–recall performance was likewise stable (average precision 0.68 vs. 0.70 pre-COVID-19; 0.27 vs. 0.26 during/post-COVID-19). Because all principal findings — the dominance of temperature, the leading lagged-temperature predictors, and the prominence of GPP — persist under a more stringent threshold, we are confident they are not artefacts of the median cut-point. These analyses are now reported in the Supplementary Information (S3 Text, Fig S7, and Table S2) and are referenced in the Discussion.

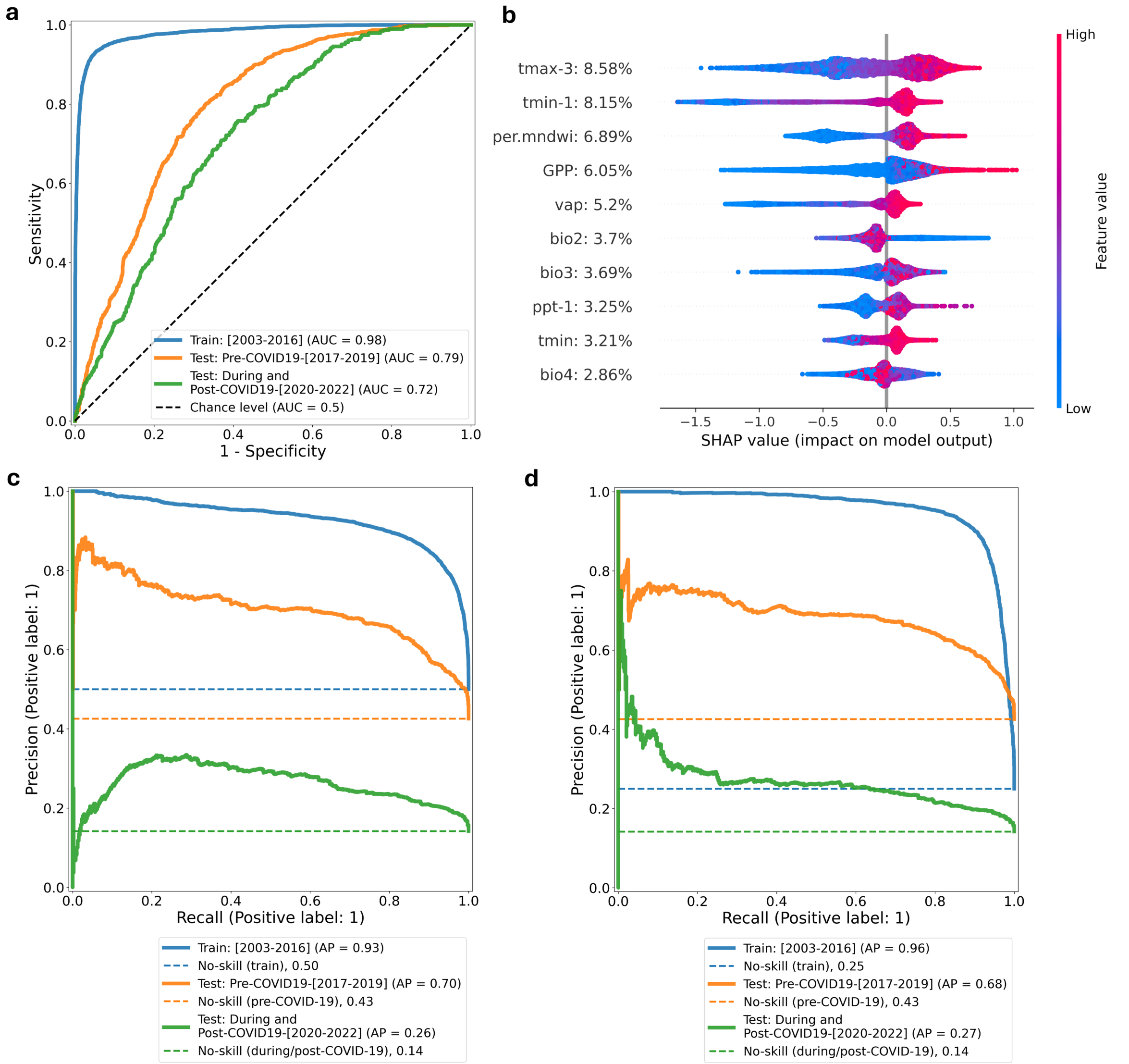

Fig S7. Sensitivity of model performance and feature importance to the risk-classification threshold. Results from re-running the full pipeline with the high-risk class defined by the 75th percentile of the training-period DHF incidence (25% positive observations; *scale_pos_weight* = 3) instead of the median. **(a)** ROC curves for the training (2003–2016), pre-COVID-19 test (2017–2019), and during/post-COVID-19 test (2020–2022) periods. **(b)** SHAP summary plot of the ten most important features. **(c, d)** Precision–recall curves under the median **(c)** and 75th-percentile **(d)** thresholds. In **(c)** and **(d)**, the dashed lines denote the no-skill baseline (positive-class prevalence) for each period, color-matched to the corresponding curve; under the 75th-percentile threshold the training baseline decreases from 0.50 to 0.25, consistent with the reduced positive-

class prevalence, whereas the pre-COVID-19 (0.43) and during/post-COVID-19 (0.14) baselines are essentially unchanged.

The sentences below have beed added to Discussion:

- "To confirm that these findings are not specific to the median cut-point, we repeated the analysis using a more stringent 75th-percentile incidence threshold (6.08 per 100,000); test-period discrimination (ROC-AUC 0.79 pre-COVID-19; 0.72 during/post-COVID-19) and the SHAP-based dominance of temperature (six of the ten leading features) were mainly preserved (**S3 Text**, **Fig S7**, **Table S2**)."

S3 Text and Table S2 read:

S3 Text: Sensitivity of results to the risk-classification threshold

Our primary analysis defines the high-risk class using the national median of the training-period DHF incidence, which balances the two classes (50% positive) and limits majority-class bias. To assess whether our conclusions depend on this choice, we repeated the complete modelling pipeline with the high-risk class defined instead by the 75th percentile of the training-period incidence distribution (6.08 cases per 100,000), yielding about 25% positive observations. To accommodate the resulting 3:1 class imbalance we set *scale_pos_weight* = 3 and re-optimized all XGBoost hyperparameters for the re-labelled target using the same Bayesian search, stratified 5-fold cross-validation, and early-stopping procedure described in the main text; the temporal train/test split and the 54-feature set were unchanged.

Out-of-sample discrimination was essentially unchanged relative to the median-based model: the test ROC-AUC was 0.79 for the pre-COVID-19 period (vs. 0.80) and 0.72 for the during- and post-COVID-19 period (vs. 0.74) (**Fig. S7a**). The SHAP feature-importance ranking remained temperature-dominated, with six of the ten leading features temperature-related, 3-month-lagged maximum temperature and 1-month-lagged minimum temperature the two most important predictors, and Gross Provincial Product again ranked fourth (**Fig. S7b**). Precision–recall performance was likewise

stable: average precision was 0.68 (vs. 0.70) for the pre-COVID-19 period and 0.27 (vs. 0.26) for the during- and post-COVID-19 period (**Fig. S7c,d**), remaining above the corresponding no-skill baseline in each period. A side-by-side summary is provided in **Table S2**.

Table S2. Comparison of model performance and key feature-importance findings under the median and 75th-percentile risk-classification thresholds.

**Measure**	**Median threshold (~50% positive)**	**75th-percentile threshold (~25% positive)**
Test-set discrimination and precision–recall		
ROC-AUC, pre-COVID-19 (2017–2019)	0.80	0.79
ROC-AUC, during/post-COVID-19 (2020–2022)	0.74	0.72
Average precision, pre-COVID-19 (2017–2019)	0.70	0.68
Average precision, during/post-COVID-19 (2020–2022)	0.26	0.27
Feature importance (SHAP)		
Temperature-related features in top 10	7	6
Two most important features	tmin-1, tmin	tmax-3, tmin-1
Gross Provincial Product (GPP) rank	4	4

Please note that while preparing this revision, we identified an error in the analysis underlying the previously submitted Fig. 3 to address the reviewer's Comment 1 (Time-Series Diagnostics) in the previous round of revision. In computing the residual ACF, the per-province residual series had been ordered by calendar month rather than chronologically (by year and month). Because the ACF is defined over a time-ordered sequence, this reordering removed the temporal structure and produced an artifactually flat ACF. We have corrected the ordering and regenerated Fig. 3. The corrected analysis shows significant positive autocorrelation at short lags (lag-1 = 0.54, with lags 1–3 outside the 95% confidence band). We have updated Fig. 3, revised the corresponding paragraph in "Model performance and validation," and added a discussion of this residual autocorrelation as a limitation. This autocorrelation is concentrated at short lags. To

confirm that it does not affect our conclusions, we added a sensitivity analysis (new **S2 Text**): adding a single autoregressive predictor (the previous month's observed risk status) removes the residual autocorrelation and improves discrimination, while the SHAP-based feature importances—temperature foremost—are preserved. We therefore retain the parsimonious environmental and socioeconomic model in the main text, consistent with our objective of identifying transmission drivers. The analysis codes in the shared repository have been updated to fix this error (repository DOI: https://doi.org/10.5281/zenodo.21053822). We thank the reviewer for prompting this diagnostic and apologize for the earlier error.

The updated Fig. 3 reads:

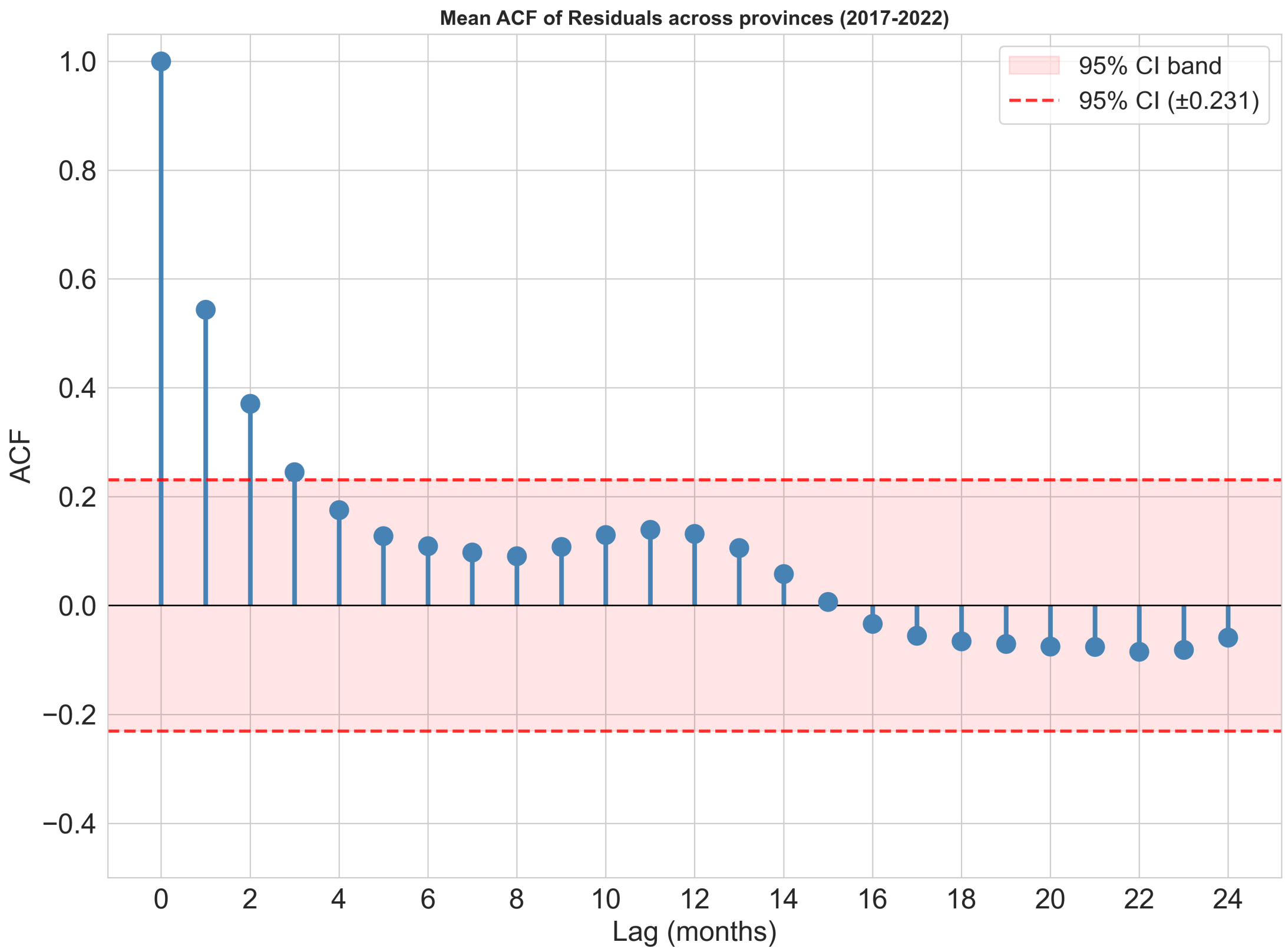

Figure 3. Mean autocorrelation function (ACF) of model residuals across Thai provinces, testing period (2017–2022). Residuals were computed as the difference between predicted and observed probabilities of high-risk classification for each province-month. The ACF was calculated separately for each province at each lag and then averaged across the 77 provinces to summarize the country-level temporal

structure. The red shaded region denotes the 95% confidence band under the null hypothesis of no autocorrelation ($\pm 1.96/\sqrt{T}$, with T = 72 months per province). Mean residual ACF values exceed the band at lags 1–3, indicating significant short-range positive autocorrelation in the residuals.

The second paragraph in "XGBoost model implementation" has been revised, which now reads:

- "...We also did not detrend or deseasonalize the input features: XGBoost does not require the strict stationarity assumed by traditional time-series models such as ARIMA or SARIMA. The autocorrelation analysis of model residuals did reveal significant short-lag autocorrelation, indicating that the model leaves some month-to-month temporal dependence unexplained. However, because the goal of this study is to identify environmental and socioeconomic predictors through feature-importance analysis rather than to produce point forecasts, this residual dependence is unlikely to materially affect our main conclusions; a sensitivity analysis that adds a single autoregressive predictor removed the residual autocorrelation while leaving temperature as the leading environmental driver (see Results and Discussion)."

The second paragraph in "Model performance and validation" has been revised, which now reads:

- "We further examined whether the model captured the temporal structure of the data by computing the autocorrelation function (ACF) of the residuals (the difference between predicted and observed probability of high-risk classification). Residuals were calculated separately for each province over the testing period, and the per-province ACFs were averaged across provinces at each lag to summarize the country-level temporal structure (Fig. 3). The mean residual ACF showed significant positive autocorrelation at short lags (lag-1 = 0.54, lag-2 = 0.37, lag-3 = 0.24), exceeding the 95% confidence band (±0.23) through lag 3 and falling

within the band at longer lags. This indicates that the residuals retain short-range (month-to-month) temporal structure that the contemporaneous and lagged environmental and socioeconomic features do not fully capture. To identify the source of this autocorrelation and assess whether it affects our conclusions, we performed a sensitivity analysis (S2 Text). This analysis showed that including a single autoregressive predictor (the previous month's observed high-risk status) removed the residual autocorrelation (no lag exceeded the 95% confidence band) and improved out-of-sample discrimination, while temperature remained the leading environmental driver."

The senteces below have been added to the "Limitations":

- "Finally, the residual autocorrelation analysis revealed significant short-lag (1–3 month) positive autocorrelation, reflecting our deliberate exclusion of autoregressive terms so that feature importance would capture environmental and socioeconomic drivers rather than the serial dependence of the outcome itself (see Results and S2 Text). Consequently, the model's residuals should not be treated as temporally independent, and operational month-ahead forecasting would benefit from an autoregressive structure or an explicit temporal error model."

Reviewer #3

Comment: *I sincerely appreciate the effort the authors have made to address my comments, and I thank them for their thorough revisions. In my opinion, the work is now suitable for publication.*

Response. We sincerely thank Reviewer #3 for this positive assessment and for the constructive feedback provided throughout the review process, which has substantially improved the manuscript.

We thank the Academic Editor and all reviewers again for their careful reading and constructive input, which have improved the manuscript. We hope that the revised version is now suitable for publication in PLOS Neglected Tropical Diseases.

Sincerely,

Charin Modchang, on behalf of all authors

Medical Biophysics & Disease Modeling Group, Department of Physics, Faculty of Science, Mahidol University

Email: charin.mod@mahidol.edu
