# Supplementary material for: Machine learning reveals temperature as a key predictor of dengue risk across Thailand’s provinces: A 20-year analysis
Source: PLoS Negl Trop Dis. 2026 Jul 28;20(7):e0014590. doi: 10.1371/journal.pntd.0014590 (PMC13440873; doi:10.1371/journal.pntd.0014590)
Supplement: Peer review history

# Peer review: Decision Letter 0

**DOI:** 10.1371/journal.pntd.0014590.r001

**Authors:** Richard A. Bowen, Mabel Carabali

11 Mar 2026

PNTD-D-25-01878Machine learning reveals temperature as a key predictor of dengue risk across Thailand’s provinces: A 20-year analysisPLOS Neglected Tropical DiseasesDear Dr. Modchang,Thank you for submitting your manuscript to PLOS Neglected Tropical Diseases. After careful consideration, we feel that it has merit but does not fully meet PLOS Neglected Tropical Diseases's publication criteria as it currently stands. Therefore, we invite you to submit a revised version of the manuscript that addresses the points raised during the review process. Please address all reviewer concerns, particularly Reviewer 2's.Please submit your revised manuscript by May 10 2026 11:59PM. If you will need more time than this to complete your revisions, please reply to this message or contact the journal office at plosntds@plos.org. When you're ready to submit your revision, log on to https://www.editorialmanager.com/pntd/ and select the 'Submissions Needing Revision' folder to locate your manuscript file.Please include the following items when submitting your revised manuscript:* A letter that responds to each point raised by the editor and reviewer(s). You should upload this letter as a separate file labeled 'Response to Reviewers'. This file does not need to include responses to any formatting updates and technical items listed in the 'Journal Requirements' section below.* A marked-up copy of your manuscript that highlights changes made to the original version. You should upload this as a separate file labeled 'Revised Manuscript with Track Changes'.* An unmarked version of your revised paper without tracked changes. You should upload this as a separate file labeled 'Manuscript'.If you would like to make changes to your financial disclosure, competing interests statement, or data availability statement, please make these updates within the submission form at the time of resubmission. Guidelines for resubmitting your figure files are available below the reviewer comments at the end of this letter.We look forward to receiving your revised manuscript. Kind regards,

Richard A. Bowen, DVM PhD

Academic Editor

PLOS Neglected Tropical Diseases

Mabel CarabaliSection EditorPLOS Neglected Tropical Diseases

Shaden Kamhawi

co-Editor-in-Chief

PLOS Neglected Tropical Diseases

orcid.org/0000-0003-4304-636XX

Paul Brindley

co-Editor-in-Chief

PLOS Neglected Tropical Diseases

orcid.org/0000-0003-1765-0002

**Additional Editor Comments:** Your manuscript has been reviewed by three experts, each of whom judged it to be a valuable contribution, but each had comments for your to consider than may increase the clarity and value of your work. Please evaluate these comments, edit your manuscript as you think best and re-submit with a response to reviewers document that describes your comments on the reviewer suggestions.**Journal Requirements:**

1) We ask that a manuscript source file is provided at Revision. Please upload your manuscript file as a .doc, .docx, .rtf or .tex. If you are providing a .tex file, please upload it under the item type u2018LaTeX Source Fileu2019 and leave your .pdf version as the item type u2018Manuscriptu2019.

2) Please upload all main figures as separate Figure files in .tif or .eps format. For more information about how to convert and format your figure files please see our guidelines:

https://journals.plos.org/plosntds/s/figures

3) We have noticed that you have uploaded Supporting Information files, but you have not included a list of legends. Please add a full list of legends for your Supporting Information files after the references list.

4) Some material included in your submission may be copyrighted. According to PLOSu2019s copyright policy, authors who use figures or other material (e.g., graphics, clipart, maps) from another author or copyright holder must demonstrate or obtain permission to publish this material under the Creative Commons Attribution 4.0 International (CC BY 4.0) License used by PLOS journals. Please closely review the details of PLOSu2019s copyright requirements here: PLOS Licenses and Copyright. If you need to request permissions from a copyright holder, you may use PLOS's Copyright Content Permission form.

Please respond directly to this email and provide any known details concerning your material's license terms and permissions required for reuse, even if you have not yet obtained copyright permissions or are unsure of your material's copyright compatibility. Once you have responded and addressed all other outstanding technical requirements, you may resubmit your manuscript within Editorial Manager.

Potential Copyright Issues:

- Figure 1:  Please (a) provide a direct link to the base layer of the map (i.e., the country or region border shape) and ensure this is also included in the figure legend; and (b) provide a link to the terms of use / license information for the base layer image or shapefile. We cannot publish proprietary or copyrighted maps (e.g. Google Maps, Mapquest) and the terms of use for your map base layer must be compatible with our CC BY 4.0 license.

Note: if you created the map in a software program like R or ArcGIS, please locate and indicate the source of the basemap shapefile onto which data has been plotted.

If your map was obtained from a copyrighted source please amend the figure so that the base map used is from an openly available source. Alternatively, please provide explicit written permission from the copyright holder granting you the right to publish the material under our CC BY 4.0 license.

If you are unsure whether you can use a map or not, please do reach out and we will be able to help you. The following websites are good examples of where you can source open access or public domain maps:

* U.S. Geological Survey (USGS) - All maps are in the public domain. (http://www.usgs.gov)

* PlaniGlobe - All maps are published under a Creative Commons license so please cite u201cPlaniGlobe, http://www.planiglobe.com, CC BY 2.0u201d in the image credit after the caption. (http://www.planiglobe.com/?lang=enl)

* Natural Earth - All maps are public domain. (http://www.naturalearthdata.com/about/terms-of-use/).

5) Please amend your detailed Financial Disclosure statement. This is published with the article. It must therefore be completed in full sentences and contain the exact wording you wish to be published.

State the initials, alongside each funding source, of each author to receive each grant. For example: "This work was supported by the National Institutes of Health (####### to AM; ###### to CJ) and the National Science Foundation (###### to AM).".

If you did not receive any funding for this study, please simply state: u201cThe authors received no specific funding for this work.u201d

**Reviewers' comments:** Reviewer's Responses to Questions

**Key Review Criteria Required for Acceptance?**

As you describe the new analyses required for acceptance, please consider the following:

**Methods:**

-Are the objectives of the study clearly articulated with a clear testable hypothesis stated?

-Is the study design appropriate to address the stated objectives?

-Is the population clearly described and appropriate for the hypothesis being tested?

-Is the sample size sufficient to ensure adequate power to address the hypothesis being tested?

-Were correct statistical analysis used to support conclusions?

-Are there concerns about ethical or regulatory requirements being met?

Reviewer #1: - Clear Objectives/Hypothesis: Yes.

- Appropriate Study Design: Partially. The workflow is standard, the study has significant "blind spots" regarding clinical scope.

- Population/Sample Size: Appropriate, though limited to specific manifestations.

- Statistical Analysis: Correct.

- Ethical Requirements: Yes.

*Comments to include*

The manuscript would benefit from a methodological flowchart to help readers visualise the machine learning framework and interpretability steps. For an example of a workflow figure, the authors may refer to Figure 1 in Mobin, M. Multivariate forecasting of dengue infection in Bangladesh: evaluating the influence of data downscaling on machine learning predictive accuracy. BMC Infect Dis 25, 761 (2025). https://doi.org/10.1186/s12879-025-11159-z

Reviewer #2: - Time-Series Diagnostics: Since this is a 20-year monthly dataset, the authors must report the Autocorrelation Function (ACF) of the residuals. Without this, it is unclear if the model has adequately accounted for temporal dependencies or if the residuals contain unresolved patterns. - Benchmarking: The study lacks a baseline comparison (e.g., logistic regression or a GAM). Without a simpler benchmark, the performance gain of the XGBoost model cannot be objectively evaluated. - Calibration: The evaluation relies on AUC/F1 scores (discrimination) but lacks probability calibration (e.g., calibration plots or Brier scores). This is essential for public health tools where the accuracy of the risk probability is as important as the final classification.

Reviewer #3: Yes, but see my general comments.

**********

**Results**

-Does the analysis presented match the analysis plan?

-Are the results clearly and completely presented?

-Are the figures (Tables, Images) of sufficient quality for clarity?

Reviewer #1: - Match Analysis Plan: Yes.

- Clear Presentation: Needs improvement regarding data visualisation.

- Figure/Table Quality: Requires revisions for clarity.

*Main Manuscript*

1. Table 1: The current format of the table is hard to understand. To improve the overall clarity and reproducibility of the work, the table should be reorganised into a more detailed format with the following columns:

- Index Number

- Variable/Feature Name

- Temporal Granularity

- Data Format

- Description (Unit)

- Source

2. Figure 3: The provinces under the y-axis are unsorted (or sorted based on a specific parameter). To improve the overall readability of the figure, the list under the y-axis should be sorted alphabetically from top to bottom. If the authors have specific reasons for using another sorting criterion, the basis for the sorting should be clearly stated in the figure’s legend.

3. Figure 5a: The legend/label box currently overlaps with the plot lines. To address this, the label should be positioned in such a way that the AUC curves, as well as the diagonal reference line, are visible.

*Supplementary Materials*

1. Figure S1 & Figure S5: To improve the overall comparison between the data distribution in Figure S1 and Figure S5, the x-axis (Month) in Figure S1 should be formatted similarly to Figure S5. Additionally, the inclusion of statistical values (e.g., mean, standard deviation, maximum, and minimum) would be beneficial in addition to the raw numerical values/percentages included in the figures.

2. Figure S3: Similar to Figure 3, the list under the y-axis should be sorted alphabetically from top to bottom to ensure consistency throughout the research paper, including the supplementary materials.

Figure S4: The current format of the variables’ visualisation is cluttered, with the variable/feature name overlapping with the values, which makes interpretation more complicated. To address this, the spacing should be increased or the font size modified to ensure the text is not overlapping. It is highly recommended to group the variables based on categories (e.g., Temperature, Precipitation, Socioeconomic) using the variable name instead of the abbreviation, thereby making the heatmap self-explanatory.

Reviewer #2: The results are well-presented, particularly the SHAP plots, which offer excellent transparency into the model's decision-making process. The identification of Tmin as a primary driver is significant. However, the "Results" section would be strengthened by including a calibration analysis (Brier score or calibration plot) to demonstrate the reliability of the predicted risk probabilities. Furthermore, the performance dip during the 2021 COVID-19 period is a critical result that requires more nuanced reporting to distinguish between model failure and genuine epidemiological decoupling.

Reviewer #3: Yes

**********

**Conclusions**

-Are the conclusions supported by the data presented?

-Are the limitations of analysis clearly described?

-Do the authors discuss how these data can be helpful to advance our understanding of the topic under study?

-Is public health relevance addressed?

Reviewer #1: No comments.

Reviewer #2: The conclusions are generally supported by the data, but the link between the statistical findings and biological mechanisms is currently thin. The authors should more explicitly connect the Tmin thresholds to the biological limits of the Aedes mosquito or the viral extrinsic incubation period (EIP). Additionally, the conclusion regarding the model’s use for public health officials should be tempered until probability calibration is performed.

Reviewer #3: Yes, but see my general comments

**********

**Editorial and Data Presentation Modifications?**

Use this section for editorial suggestions as well as relatively minor modifications of existing data that would enhance clarity. If the only modifications needed are minor and/or editorial, you may wish to recommend “Minor Revision” or “Accept”.

Reviewer #1: *Introduction*

Structural Logic: To improve the logical flow, consider moving the discussion of virus characteristics (lines 89–95) to follow the first paragraph.

*Literature Update*

Reference [11] (Verma et al., 2014), reference to neurological manifestations in lines 91–93 appears irrelevant to the discussion on clinical manifestations and should be deleted or replaced with a sentence about the spectrum of Severity.

Reference [30] (Suttirat et al.) is currently a preprint. Please update this with the peer-reviewed version.

Reviewer #2: Please provide a more detailed step-by-step clarification on the interpolation methods used to synchronize biennial socioeconomic data with monthly climatic records.

Reviewer #3: (No Response)

**********

**Summary and General Comments**

Use this section to provide overall comments, discuss strengths/weaknesses of the study, novelty, significance, general execution and scholarship. You may also include additional comments for the author, including concerns about dual publication, research ethics, or publication ethics. If requesting major revision, please articulate the new experiments that are needed.

Reviewer #1: The manuscript is a well-written study. Although the machine learning workflow for classification is standard, the study has significant "blind spots" regarding the clinical scope of the disease and the lack of analysis of different models. These are areas that will be significant for improvement, besides improvements to data visualisation, to ensure that the study reflects the data accurately.

Please note the following additional limitations or suggestions for future research in your Discussion section:

1. The authors exclusively use Dengue Hemorrhagic Fever (DHF) data and do not include the full clinical spectrum of Dengue Fever (DF) and Dengue Shock Syndrome (DSS). At a minimum, the authors must provide a clear rationale in the Introduction explaining why only DHF was selected and why other manifestations were excluded.

2. While the classification workflow is standard, the study lacks a comparison between different prediction models. Including such a comparison would strengthen the validity of the chosen framework.

3. The inclusion of analysis code and data is available; the authors would enhance the study's impact and accessibility by hosting these on a public repository such as GitHub. This would facilitate better version control for future updates and streamline the ability for other researchers to reproduce these findings.

Reviewer #2: This manuscript provides a valuable 20-year analysis of DHF risk in Thailand using an interpretable machine learning approach. The use of SHAP to move beyond "black-box" predictions is highly commendable and aligns with the needs of public health stakeholders.

However, the manuscript currently lacks several standard statistical safeguards necessary for time-series modeling. The most critical omission is the lack of residual autocorrelation (ACF) testing and a baseline model comparison. Addressing these points, along with providing a calibration analysis, will significantly strengthen the paper’s findings and ensure the model is reliable for real-world application. I have recommended a major revision to allow the authors to incorporate these essential diagnostic steps.

Reviewer #3: The paper is clearly written, and presents interesting and somehow counterintuitive results. However the methodology section needs to be improved, in order to properly let the readers understanding how to replicate and assess the limitations of the proposed approach.

The methodology section lacks some information. A paragraph with a suitable figure that explains the entire software pipeline, with a complete description and schematic representation of of each input (predictors)/output sample.

A schematic representation of the machine learning model is also needed.

A concise mathematical description of the model is currently missing. It would be useful to properly understand the role and meaning of the hyperparameters, and how they are optimized.

A key aspect that should be explained in the implementation details is data normalization, that may improve training and testing performances. If no normalization is performed, the authors should explain why. Additionally, they should explain in detail if they filtered out means and trends from the data to capture the seasonal or time invariant dependancies.

MODEL PERFORMANCES AND VALIDATION

Training performances cannot be compared directly with testing. The authors should contrast the AUC = 0.74 with the AUC = 0.8, both derived by testing data. The overprediction may derive from the lack of normalization, and by the choice of training the model only with old data. Additionally, attributing overproduction to (increased) mobility changes is a statement that should be discussed in more detail.

FIGURE 3

Again, the lack of separation in the figure between training and testing data is misleading. Training set performances are only diagnostics of the training process, and cannot be listed as prediction results.

**********

PLOS authors have the option to publish the peer review history of their article (what does this mean?). If published, this will include your full peer review and any attached files.

If you choose “no”, your identity will remain anonymous but your review may still be made public.

**Do you want your identity to be public for this peer review?**  For information about this choice, including consent withdrawal, please see our Privacy Policy.

Reviewer #1: No

Reviewer #2: No

Reviewer #3: No

[NOTE: If reviewer comments were submitted as an attachment file, they will be attached to this email and accessible via the submission site. Please log into your account, locate the manuscript record, and check for the action link "View Attachments". If this link does not appear, there are no attachment files.]**Figure resubmission:** While revising your submission, we strongly recommend that you use PLOS’s NAAS tool (https://ngplosjournals.pagemajik.ai/artanalysis) to test your figure files. NAAS can convert your figure files to the TIFF file type and meet basic requirements (such as print size, resolution), or provide you with a report on issues that do not meet our requirements and that NAAS cannot fix.

After uploading your figures to PLOS’s NAAS tool - https://ngplosjournals.pagemajik.ai/artanalysis, NAAS will process the files provided and display the results in the "Uploaded Files" section of the page as the processing is complete. If the uploaded figures meet our requirements (or NAAS is able to fix the files to meet our requirements), the figure will be marked as "fixed" above. If NAAS is unable to fix the files, a red "failed" label will appear above. When NAAS has confirmed that the figure files meet our requirements, please download the file via the download option, and include these NAAS processed figure files when submitting your revised manuscript.**Reproducibility:** To enhance the reproducibility of your results, we recommend that authors of applicable studies deposit laboratory protocols in protocols.io, where a protocol can be assigned its own identifier (DOI) such that it can be cited independently in the future. Additionally, PLOS ONE offers an option to publish peer-reviewed clinical study protocols. Read more information on sharing protocols at https://plos.org/protocols?utm_medium=editorial-email&utm_source=authorletters&utm_campaign=protocols

# Author comment: Author response to Decision Letter 1

**DOI:** 10.1371/journal.pntd.0014590.r002

26 May 2026

Attachment Submitted filename: Response_Letter_22May26.pdf

# Peer review: Decision Letter 1

**DOI:** 10.1371/journal.pntd.0014590.r003

**Authors:** Richard A. Bowen, Abdallah M. Samy

16 Jun 2026

PNTD-D-25-01878R1Machine learning reveals temperature as a key predictor of dengue risk across Thailand’s provinces: A 20-year analysisPLOS Neglected Tropical Diseases Dear Dr. Modchang, Thank you for submitting your manuscript to PLOS Neglected Tropical Diseases. After careful consideration, we feel that it has merit but does not fully meet PLOS Neglected Tropical Diseases's publication criteria as it currently stands. Therefore, we invite you to submit a revised version of the manuscript that addresses the points raised during the review process. Please submit your revised manuscript by Aug 15 2026 11:59PM. If you will need more time than this to complete your revisions, please reply to this message or contact the journal office at plosntds@plos.org. When you're ready to submit your revision, log on to https://www.editorialmanager.com/pntd/ and select the 'Submissions Needing Revision' folder to locate your manuscript file. Please include the following items when submitting your revised manuscript:* A letter that responds to each point raised by the editor and reviewer(s). You should upload this letter as a separate file labeled 'Response to Reviewers'. This file does not need to include responses to any formatting updates and technical items listed in the 'Journal Requirements' section below.* A marked-up copy of your manuscript that highlights changes made to the original version. You should upload this as a separate file labeled 'Revised Manuscript with Track Changes'.* An unmarked version of your revised paper without tracked changes. You should upload this as a separate file labeled 'Manuscript'. If you would like to make changes to your financial disclosure, competing interests statement, or data availability statement, please make these updates within the submission form at the time of resubmission. Guidelines for resubmitting your figure files are available below the reviewer comments at the end of this letter. As the corresponding author, your ORCID iD is verified in the submission system and will appear in the published article. PLOS supports the use of ORCID, and we encourage all coauthors to register for an ORCID iD and use it as well. Please encourage your coauthors to verify their ORCID iD within the submission system before final acceptance, as unverified ORCID iDs will not appear in the published article. Only the individual author can complete the verification step; PLOS staff cannot verify ORCID iDs on behalf of authors.We look forward to receiving your revised manuscript. Kind regards, Richard A. BowenAcademic Editor Abdallah SamySection EditorPLOS Neglected Tropical Diseases

Shaden Kamhawi

co-Editor-in-Chief

PLOS Neglected Tropical Diseases

orcid.org/0000-0003-4304-636XX

Paul Brindley

co-Editor-in-Chief

PLOS Neglected Tropical Diseases

orcid.org/0000-0003-1765-0002

**Additional Editor Comments (if provided):**  Thank you for the thoughtful revision of your manuscipt. I consider this acceptable, but two of the reviewers have provided additional comments that you can consider for very minor revisions if you wish    **Reviewers' comments:**  Reviewer's Responses to Questions

**Key Review Criteria Required for Acceptance?**

As you describe the new analyses required for acceptance, please consider the following:

**Methods**

-Are the objectives of the study clearly articulated with a clear testable hypothesis stated?

-Is the study design appropriate to address the stated objectives?

-Is the population clearly described and appropriate for the hypothesis being tested?

-Is the sample size sufficient to ensure adequate power to address the hypothesis being tested?

-Were correct statistical analysis used to support conclusions?

-Are there concerns about ethical or regulatory requirements being met?

Reviewer #1: Comment 2 (Table 1 Formatting)

I appreciate the authors' effort to reorganise Table 1 ("Variables used in the XGBoost model for dengue risk classification in Thailand") into a more structured format. It is much more readable now. However, the "Data Format" column is conceptually incorrect and contains a recurring typo:

1.**Conceptual Mismatch**: The terms "Grided" and "Per province" do not actually describe data formats; instead, they describe the Spatial Scale or Spatial Resolution of the data. True "Data Format" descriptors should reflect the actual file types or data structures used in the study.

2.**Typographical Error**: The word "Gridded" is consistently misspelt as "Grided" throughout this column.

**Recommendation**: Please keep the "Data Format" column but get rid of the spatial terms ("Grided" / "Per province") within it. Instead, populate this column with the true technical data formats used (e.g., Raster, NetCDF, CSV, Shapefile, Float, or Integer).

Reviewer #2: (No Response)

Reviewer #3: (No Response)

**********

**Results**

-Does the analysis presented match the analysis plan?

-Are the results clearly and completely presented?

-Are the figures (Tables, Images) of sufficient quality for clarity?

Reviewer #1: No further comment.

Reviewer #2: (No Response)

Reviewer #3: (No Response)

**********

**Conclusions**

-Are the conclusions supported by the data presented?

-Are the limitations of analysis clearly described?

-Do the authors discuss how these data can be helpful to advance our understanding of the topic under study?

-Is public health relevance addressed?

Reviewer #1: No further comment.

Reviewer #2: (No Response)

Reviewer #3: (No Response)

**********

**Editorial and Data Presentation Modifications?**

Use this section for editorial suggestions as well as relatively minor modifications of existing data that would enhance clarity. If the only modifications needed are minor and/or editorial, you may wish to recommend “Minor Revision” or “Accept”.

Reviewer #1: Comment 10

*Introduction*(Case Definition: DHF vs. DF/DSS)

Thank you for the authors' explanation regarding the operational trade-offs of using Dengue Hemorrhagic Fever (DHF) data. The current revision does not fully satisfy the recommendation.

The newly added rationale in the Introduction still requires a minor adjustment to be fully satisfactory:

**Lack of Evidence/Citations**: In the newly added paragraph in the Introduction, the authors state that DF is "systematically under-reported" and DSS is "too infrequent to support province-level monthly modelling." While these claims may be logically sound, they are currently unsupported. The authors must provide citations to prove that DF is under-reported in this specific context and that DSS data is too sparse.

**Recommendation**: The manuscript will be fully resolved once the authors add appropriate supporting citations to back up these claims in the Introduction.

Reviewer #2: (No Response)

Reviewer #3: (No Response)

**********

**Summary and General Comments**

Use this section to provide overall comments, discuss strengths/weaknesses of the study, novelty, significance, general execution and scholarship. You may also include additional comments for the author, including concerns about dual publication, research ethics, or publication ethics. If requesting major revision, please articulate the new experiments that are needed.

Reviewer #1: No further comment.

Reviewer #2: (No Response)

Reviewer #3: I sincerely appreciate the effort the authors have made to address my comments, and I thank them for their thorough revisions. In my opinion, the work is now suitable for publication.

**********

PLOS authors have the option to publish the peer review history of their article (what does this mean?). If published, this will include your full peer review and any attached files.

If you choose “no”, your identity will remain anonymous but your review may still be made public.

**Do you want your identity to be public for this peer review?**  For information about this choice, including consent withdrawal, please see our Privacy Policy.

Reviewer #1: No

Reviewer #2: No

Reviewer #3: No

[NOTE: If reviewer comments were submitted as an attachment file, they will be attached to this email and accessible via the submission site. Please log into your account, locate the manuscript record, and check for the action link "View Attachments". If this link does not appear, there are no attachment files.] **Figure resubmission:**  While revising your submission, we strongly recommend that you use PLOS’s NAAS tool (https://ngplosjournals.pagemajik.ai/artanalysis) to test your figure files. NAAS can convert your figure files to the TIFF file type and meet basic requirements (such as print size, resolution), or provide you with a report on issues that do not meet our requirements and that NAAS cannot fix.

After uploading your figures to PLOS’s NAAS tool - https://ngplosjournals.pagemajik.ai/artanalysis, NAAS will process the files provided and display the results in the "Uploaded Files" section of the page as the processing is complete. If the uploaded figures meet our requirements (or NAAS is able to fix the files to meet our requirements), the figure will be marked as "fixed" above. If NAAS is unable to fix the files, a red "failed" label will appear above. When NAAS has confirmed that the figure files meet our requirements, please download the file via the download option, and include these NAAS processed figure files when submitting your revised manuscript. **Reproducibility:**  To enhance the reproducibility of your results, we recommend that authors of applicable studies deposit laboratory protocols in protocols.io, where a protocol can be assigned its own identifier (DOI) such that it can be cited independently in the future. Additionally, PLOS ONE offers an option to publish peer-reviewed clinical study protocols. Read more information on sharing protocols at https://plos.org/protocols?utm_medium=editorial-email&utm_source=authorletters&utm_campaign=protocols

Attachment Submitted filename: Reviewer Comments on Revised Manuscript_PLoS_0602.pdf

# Author comment: Author response to Decision Letter 2

**DOI:** 10.1371/journal.pntd.0014590.r004

2 Jul 2026

Attachment Submitted filename: Response_Letter_2July26.pdf

# Editor report: Decision Letter 2

**DOI:** 10.1371/journal.pntd.0014590.r005

**Authors:** Richard A. Bowen, Abdallah M. Samy

20 Jul 2026

Dear Dr. Modchang,

We are pleased to inform you that your manuscript 'Machine learning reveals temperature as a key predictor of dengue risk across Thailand’s provinces: A 20-year analysis' has been provisionally accepted for publication in PLOS Neglected Tropical Diseases.

Before your manuscript can be formally accepted you will need to complete some formatting changes, which you will receive in a follow up email. A member of our team will be in touch with a set of requests.

Please note that your manuscript will not be scheduled for publication until you have made the required changes, so a swift response is appreciated.

IMPORTANT: The editorial review process is now complete. PLOS will only permit corrections to spelling, formatting or significant scientific errors from this point onwards. Requests for major changes, or any which affect the scientific understanding of your work, will cause delays to the publication date of your manuscript.

Should you, your institution's press office or the journal office choose to press release your paper, you will automatically be opted out of early publication. We ask that you notify us now if you or your institution is planning to press release the article. All press must be co-ordinated with PLOS.

Thank you again for supporting Open Access publishing; we are looking forward to publishing your work in PLOS Neglected Tropical Diseases.

Best regards,

Richard A. Bowen, DVM PhD

Academic Editor

PLOS Neglected Tropical Diseases

Abdallah Samy

Section Editor

PLOS Neglected Tropical Diseases

Shaden Kamhawi

co-Editor-in-Chief

PLOS Neglected Tropical Diseases

orcid.org/0000-0003-4304-636XX

Paul Brindley

co-Editor-in-Chief

PLOS Neglected Tropical Diseases

orcid.org/0000-0003-1765-0002

***********************************************************

# Editor report: Acceptance letter

**DOI:** 10.1371/journal.pntd.0014590.r006

**Authors:** Richard A. Bowen, Abdallah M. Samy

Dear Dr. Modchang,

We are delighted to inform you that your manuscript, "Machine learning reveals temperature as a key predictor of dengue risk across Thailand’s provinces: A 20-year analysis," has been formally accepted for publication in PLOS Neglected Tropical Diseases.

We have now passed your article onto the PLOS Production Department who will complete the rest of the publication process. All authors will receive a confirmation email upon publication.

The corresponding author will soon be receiving a typeset proof for review, to ensure errors have not been introduced during production. Please review the PDF proof of your manuscript carefully, as this is the last chance to correct any scientific or type-setting errors. Please note that major changes, or those which affect the scientific understanding of the work, will likely cause delays to the publication date of your manuscript. Note: Proofs for Front Matter articles (Editorial, Viewpoint, Symposium, Review, etc...) are generated on a different schedule and may not be made available as quickly.

Soon after your final files are uploaded, the early version of your manuscript will be published online unless you opted out of this process. The date of the early version will be your article's publication date. The final article will be published to the same URL, and all versions of the paper will be accessible to readers.

For Research Articles, you will receive an invoice from PLOS for your publication fee after your manuscript has reached the completed accept phase. If you receive an email requesting payment before acceptance or for any other service, this may be a phishing scheme. Learn how to identify phishing emails and protect your accounts at https://explore.plos.org/phishing.

Thank you again for supporting open-access publishing; we are looking forward to publishing your work in PLOS Neglected Tropical Diseases.

Best regards,

Shaden Kamhawi

co-Editor-in-Chief

PLOS Neglected Tropical Diseases

Paul Brindley

co-Editor-in-Chief

PLOS Neglected Tropical Diseases
